# A cluster-randomized crossover trial of organic diet impact on biomarkers of exposure to pesticides and biomarkers of oxidative stress/inflammation in primary school children

**DOI:** 10.1371/journal.pone.0219420

**Published:** 2019-09-04

**Authors:** Konstantinos C. Makris, Corina Konstantinou, Xanthi D. Andrianou, Pantelis Charisiadis, Alexis Kyriacou, Matthew O. Gribble, Costas A. Christophi

**Affiliations:** 1 Cyprus International Institute for Environmental and Public Health, Cyprus University of Technology, Limassol, Cyprus; 2 Faculty of Health Sciences and Sport, University of Stirling, Stirling, Scotland, United Kingdom; 3 Department of Environmental Health, Emory University, Atlanta, GA, United States of America; 4 Department of Epidemiology, Emory University, Atlanta, GA, United States of America; University of Mississippi Medical Center, UNITED STATES

## Abstract

Despite suggestive observational epidemiology and laboratory studies, there is limited experimental evidence regarding the effect of organic diet on human health. A cluster-randomized 40-day-organic (vs. 40-day-conventional) crossover trial was conducted among children (11–12 years old) from six schools in Cyprus. One restaurant provided all organic meals, and adherence to the organic diet intervention was measured by parent-provided diet questionnaire/diary data. Biomarkers of pyrethroid and neonicotinoid pesticide exposures were measured using tandem mass spectrometry, and oxidative stress/inflammation (OSI) biomarkers using immunoassays or spectrophotometry. Associations were assessed using mixed-effect regression models including interactions of treatment with time. Seventy-two percent of neonicotinoid biomarkers were non-detectable and modeled as binary (whether detectable). In post-hoc analysis, we considered the outcome of age-and-sex-standardized BMI. Multiple comparisons were handled using Benjamini-Hochberg correction for 58 regression parameters. Outcome data were available for 149 children. Children had lower pesticide exposures during the organic period (pyrethroid geometric mean ratio, GMR = 0.297; [95% confidence interval (95% CI): 0.237, 0.373], Q-value<0.05); odds for detection of neonicotinoids (OR = 0.651; [95% CI: 0.463, 0.917), Q-value<0.05); and decreased OSI biomarker 8-OHdG (GMR = 0.888; [95% CI: 0.808, 0.976], Q-value<0.05). An initial increase was followed by a countervailing decrease over time in the organic period for OSI biomarkers 8-iso-PGF2a and MDA. BMI z-scores were lower at the end of the organic period (β = -0.131; [95% CI: 0.179, -0.920], Q-value<0.05). Energy intake during the conventional period was reported to be higher than the recommended reference levels. The organic diet intervention reduced children’s exposure to pyrethroid and neonicotinoid pesticides and, over time lowered biomarkers of oxidative stress/inflammation (8-iso-PGF2a, 8-OHdG and MDA). The several-week organic diet intervention also reduced children’s age-and-sex-standardized BMI z-scores, but causal inferences regarding organic diet’s physiological benefits are limited by the confounding of the organic diet intervention with caloric intake reduction and possible lifestyle changes during the trial.

**Trial registration:** This trial is registered with ClinicalTrials.gov, number: NCT02998203.

## Introduction

Behavioral interventions focused on dietary and other modifiable lifestyle factors are of growing interest for health care management and prevention of chronic disease [1,2]. Prospective observational studies in adults have shown that an organic diet reduces the risk of being overweight or obese, but the evidence is inconclusive due to likely residual confounding, as consumers of organic food tend to have overall healthier lifestyles [3]. Several animal models suggest the implication of pyrethroid and neonicotinoid pesticides with oxidative stress and inflammation phenomena (OSI) [4,5] and adiposity/weight gain [6,7]. Some human studies have found associations between exposure to other pesticides (organophosphates) and oxidative stress [8–10]. However, experimental evidence regarding the health impact of organic diet is limited. Previous organic diet intervention trials focused on reducing the magnitude of OSI biomarkers of effect were characterized by low adherence to CONSORT reporting guidelines, short intervention duration (12–22 days) and modest sample sizes (10–130 adults) [11–15].

Healthy dietary habits during childhood are crucial for optimal growth and cognitive development [16]. Unhealthy diets often lead to obesity, which is one of the most well-known public health challenges, becoming nearly a global epidemic [16]. In Cyprus, an increase in the prevalence of obesity over a decade was reported for children and adolescents 6–17 years old [from 5.9% (95% Confidence Interval (CI): 5.0–6.8) in 1999–2000 to 8.1% (95% CI: 7.2–9.1) in 2009–2010)] [17], while the WHO obesity prevalence estimates for 2015–2017 were 21% and 19% for boys and girls aged 6–9 years old, respectively [18].

The primary objective of this non-pharmacological trial was to determine the effectiveness of an organic diet intervention in reducing the body burden of urinary concentrations of pyrethroid and neonicotinoid pesticide metabolites and, secondarily, to evaluate its effect on biomarkers of OSI in primary school children in Cyprus.

## Methods

### Trial oversight

**Fig 1:**
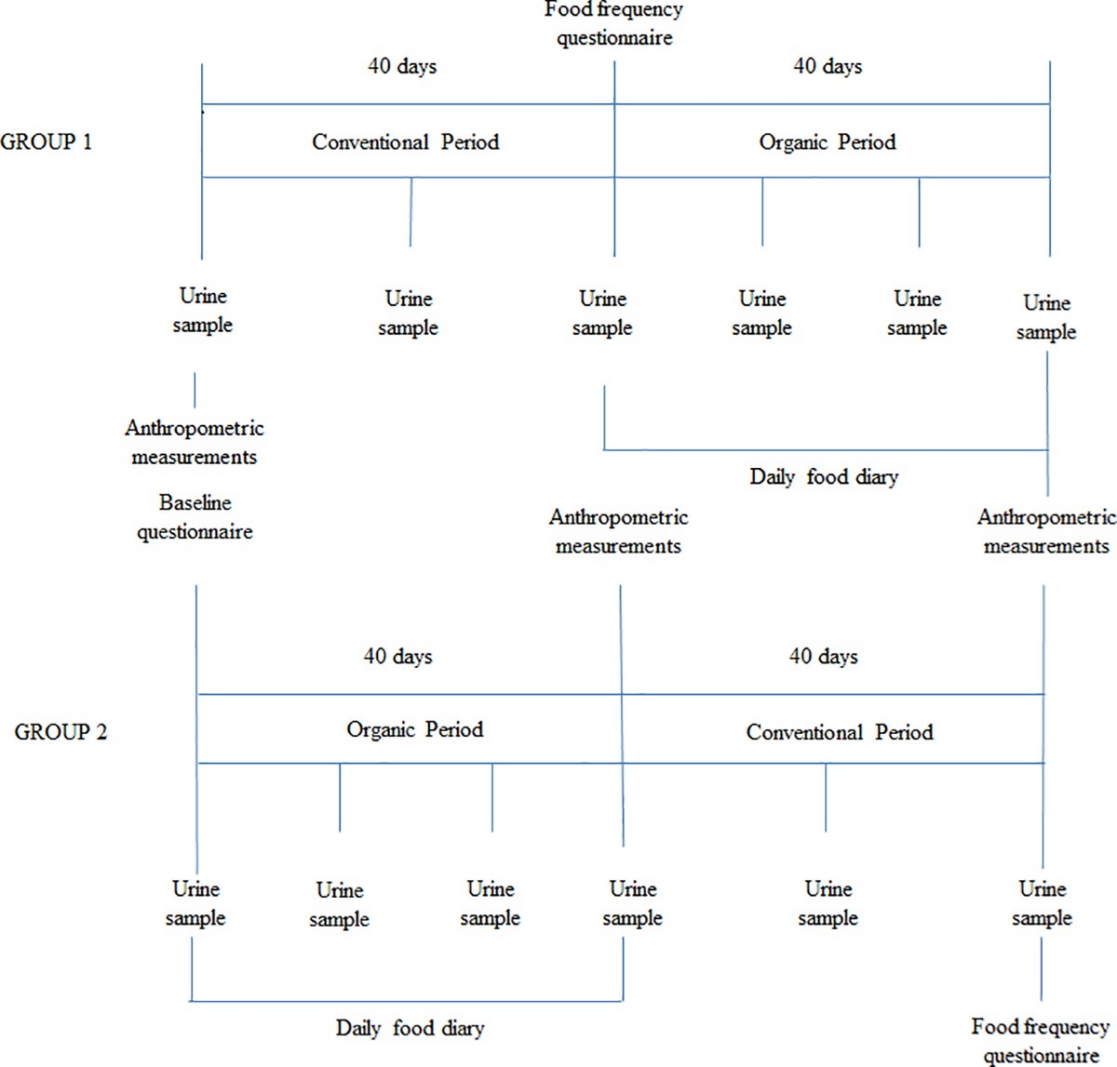
Study timeline and data collection procedure for the two groups of the study.

The ORGANIKO LIFE+ study was an investigator-initiated 2 x 2 cluster (school)-based, randomized crossover trial. The trial was conducted in six primary schools with two periods (40-days organic diet vs. 40-days of conventional diet) in Limassol, Cyprus during January-April 2017 (recruitment and intervention timeframe) (Fig 1). The organic products used for preparing the meals of the organic dietary menus were prepared by a restaurant abiding by the requirements of the EU Regulations No. 834/2007 on Organic Food and Farming and No. 889/2008 on Organic Production and Labelling of Organic Products. The full trial design can be found in the supporting information (S1 Text). The trial protocol was approved by the Cyprus National Bioethics Committee (EEBK/EΠ/2016/25, dated 07/05/2016) and the Cyprus Ministry of Education and Culture (7.15.06.15/2). The trial was performed in accordance with the principles of the Declaration of Helsinki. The authors assume responsibility for the accuracy and completeness of the data and analyses, as well as for the fidelity of the trial. The authors confirm that all ongoing and related trials for this drug/intervention are registered.

### Trial population

**Fig 2:**
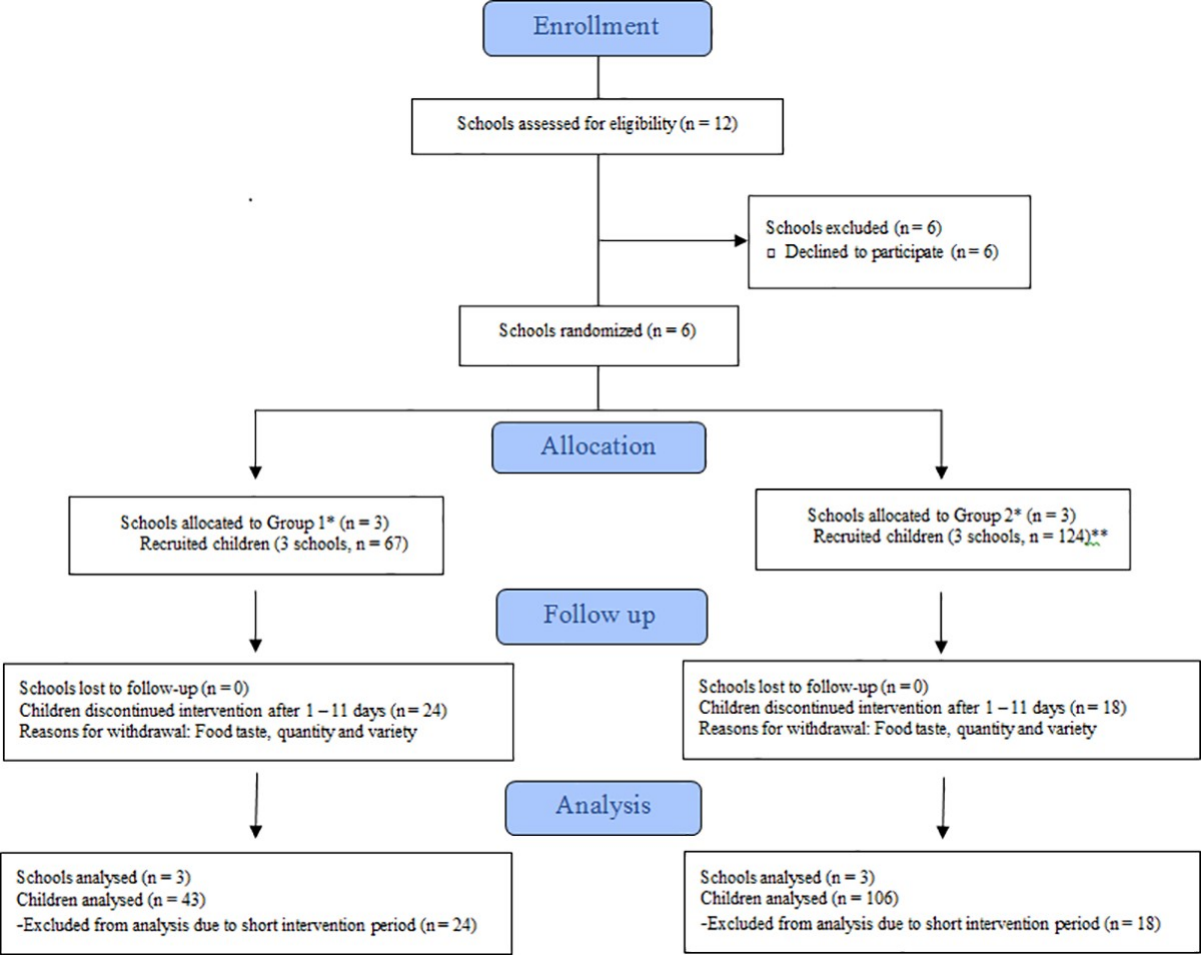
Flow diagram of participants included in the analysis. *Group 1: First organic period that was followed by the conventional period, Group 2: First conventional period that was followed by the organic period **Two children followed the opposite design compared to the rest children because they decided to participate after the trial had already started, at the end of the conventional period. These two children started with the second leg of the trial (organic diet) and then continued with the conventional diet.

The following eligibility criteria were set for the clusters (schools): i) being a public primary school, and ii) being located in the urban area of Limassol, Cyprus (Fig 2). Eligible participants were healthy 10-12-year old primary school children (5^th^ and 6^th^ grade), who had been living in Cyprus for at least the previous five years and were systematically consuming conventional food (>80% of a week’s meals) prior to the study recruitment. Eligible participants with any self-reported chronic disease conditions (e.g., asthma, type I diabetes or other chronic disease) or food allergies (e.g., to gluten or lactose tolerance) were excluded. Informed consent was obtained from the school headmaster, a written informed consent was provided by the children’s parents or legal guardians, and a verbal assent was obtained from the children.

### Randomization and masking

Schools were randomized *a priori* to two groups that differed in the sequence of the treatments; organic diet followed by conventional diet (Group 1) or conventional diet followed by organic diet (Group 2). The participant ratio of Group 1: Group 2 was 1: 2.5. Details on the reasons for the cluster randomization and the recruitment process are available in the supporting information (S1 Text). Briefly, the blinding of the participants to group assignment was not possible, since participants knew which diet they were following. The blinding of the researchers to the participants’ identity was achieved by the coding of all study materials (urine containers, questionnaires, and diaries). The study personnel who performed the sample analyses were kept masked to the allocation.

### Trial procedures

The recruitment process started with contacting randomly selected schools and then organizing meetings with parents and children to inform them about the study. After expression of interest to participate in the study, eligibility criteria were checked by the research team. Upon signing of informed consent forms, study materials (i.e., first morning urine void sample collection instruction, coded vials for urine collection, and food diaries) were provided. During the conventional period, participants were asked to maintain their usual dietary choices (>80% conventional diet) for a total of 40 days. During the organic period, participants were asked to follow strictly the two ~20-day sequential organic dietary menus provided to them for 40±3 days. The organic dietary menus were prepared by a registered dietitian based on the European Food Safety Agency (EFSA) guidelines for energy intake of 10–12 years old children [19], and included five meals per day (breakfast, morning snack, lunch, afternoon snack, and dinner). The fully prepared meals were delivered daily by the restaurant to the schools where the children were collecting them (S1 Text). Participants crossed over to the alternate diet on the day after the first period was completed. A washout period was not required as it was intrinsically included in the two periods, since the first urine sample of the second period was collected about 12 days after the beginning of the second period. Moreover, the pesticide half-lives are short (half-lives ranging 6.4–16.5 hours for pyrethroids and 5–33 hours for neonicotinoids), so no carryover effect was expected [20,21].

Each participant provided up to six first morning urine samples during the whole duration of the 2-period study; one baseline sample and two samples in the conventional period, and three samples in the organic period. Standardized methods were adopted for the anthropometric measurements (weight, height, and waist circumference), which were taken at the beginning of the study, at the end of the organic period, and at the end of the study (for Group 2, the end of study and end of organic period was the same time point) by trained researchers (S1 Text) [22]. Besides the baseline questionnaire, a food frequency questionnaire was also administered to the parents at the end of the conventional period through a telephone interview to collect information about the food habits of the children during the conventional period. All parents were asked to complete a food diary during the organic period for compliance assessment of non-organic food consumption incidences (S2 Text). The study questionnaires can be found in the supporting information (S3 Text).

### Outcomes

Per the trial protocol, the primary outcomes were the urinary biomarkers of exposure to pyrethroid pesticides (3-phenoxybenzoic acid, 3-PBA), and neonicotinoid pesticides, (6-chloronicotinic acid, 6-CN). The secondary outcomes were the biomarkers of oxidative stress/inflammation (8-iso-prostaglandin F2a [8-iso-PGF2a], malondialdehyde [MDA], and 8-hydroxy-2′-deoxyguanosine [8-OHdG]) measured in the same urine samples.

In post-hoc analyses, we considered the observational associations of 3-PBA/6-CN with OSI biomarkers as a possible mediating mechanism for the associations of the organic diet intervention with both outcomes. We also assessed the effect of the intervention on the more distal outcome of age-and sex-standardized BMI z-scores using the WHO 2007 growth reference standard for children [23]. BMI z-scores were calculated based on the measurements of weight and height taken at the baseline and at the end of the organic period (two timepoints), standardized for age and sex.

### Urine sample collection

On specific sampling dates, first morning urine voids were obtained at home in polypropylene, sterilized urine vials and collected at school by the research team. Urine vials were temporarily stored in a school/home fridge (4°C) until transferred to laboratory facilities for storage at -80°C.

### Biomarkers measurements

We measured two pesticide metabolites in urine samples: 3-PBA, a metabolite of pyrethroid pesticides, and 6-CN, a metabolite of neonicotinoid pesticides. The biomonitoring analysis was carried out using a gas-chromatographic-tandem mass spectrometric (GC–MS/MS) method based upon modifications of two existing protocols [24,25]. Quality control and quality assurance characteristics of the method can be found in the supporting information (S4 Text). The limits of detection (LOD) and limits of quantification (LOQ) (in parenthesis) were: 49 (1460) ng/L for 3-PBA, and 75 (2260) ng/L for 6-CN. A total of 854 urine samples were analyzed.

Competitive ELISA kits were used to determine urinary concentrations of 8-iso-PGF2α (Catalog no: STA-337; Cell Biolabs, Inc, California, USA) and 8-OHdG (Catalog no: ABIN2964843; antibodies-online, Aachen, Germany). The analyses were performed according to the manufacturer's instructions. Detection limits for 8-iso-PGF2α and 8-OHdG were 49 pg/mL and 0.59 ng/mL, respectively. MDA was measured using a spectrophotometric method, as previously described [26], with LOD of 0.28 μmol/L (S4 Text). Creatinine-corrected biomarker values were calculated after measurements of urinary creatinine using the colorimetric Jaffé method [27].

### Statistical analysis

Participants who followed the organic treatment for at least 12 days and provided at least one urine sample during the organic period were included in the analysis. The baseline characteristics were compared between the study groups (Group 1, Group 2) and between dropouts (i.e., enrolled students but did not participate for at least 12 days). Categorical variables were described with sample size and percentages and compared by chi-square test. Approximately normally-distributed continuous variables were described with means and standard deviations (SD), and compared by t-test, and non-normal continuous variables with medians and interquartile ranges (25^th^–75^th^ percentiles) and compared by the Wilcoxon rank sum test. The mean daily energy intake for the conventional diets was calculated based on the calories of each item of the food frequency questionnaire.

Biomarker (either pesticides or OSI) values <LOD were imputed with regression on order statistics (ROS) [28], if they contained ≥20% values below detection, or deterministically imputed as LOD/2 if <20% of the values were below detection limit. All biomarker data were corrected for urine dilution (biomarker mass per gram of urinary creatinine) prior to statistical analysis.

Changes in biomarkers between the conventional and organic treatments were assessed with: (i) the percent change between the last sample of the conventional treatment period (before the start of the organic treatment) and the last urine sample of organic treatment period, and (ii) the overall difference in median levels of biomarker concentrations between the conventional and organic phase. The percent change was estimated only for the participants who completed the full course of the organic treatment, using the log-transformed, creatinine-adjusted biomarker levels. A one-sample t-test was used to assess whether the percent change was different than zero. The overall differences in the medians of biomarkers between the conventional and the organic phase were assessed with the non-parametric Wilcoxon rank sum test on the creatinine-adjusted concentrations pooling all conventional samples (including the baseline) and the organic samples for all participants, regardless of the duration for which they followed the organic treatment.

Linear mixed-effect regression models were used to account for the duration and the effect of treatment (organic or conventional diet) where the biomarkers of exposure and OSI were the main outcomes. All models included student-level (repeated measures within person) and school-level (multiple students clustered within each school) random intercepts with an unstructured covariance matrix. Continuous variables, other than time (days of treatment), were centered at the population means. Linear models were fitted for the 3-PBA and the OSI biomarkers (log-transformed, creatinine-corrected), and, logistic models for 6-CN (binary variable; above and below LOD) due to the high number of values below LOD. A first set of models included fixed effects for treatment condition (organic or conventional) and time (days of treatment), where time = 0 was used for the start of the treatment. The models were adjusted for the baseline value (first urine sample for all children) of the outcome to account for the background participant levels. An interaction term for the day of treatment (when the sample was collected) and the treatment was considered and subsequently dropped if it did not meet the threshold of p-value<0. 05.

In *post-hoc* analyses, we used mixed-effect linear regression models to: i) describe the association of 3-PBA or 6-CN (as proxies of the treatment effect) with OSI biomarkers adjusting for the baseline value of the outcome, time, age and sex; and ii) describe the impact of organic diet on participants’ BMI scores with or without conditioning on 3-PBA or OSI biomarkers as candidate mediators.

Multiple testing was accounted for using the Benjamini-Hochberg method, considering 58 regression parameter tests of the aforementioned models. Q-value<0.05 were considered statistically significant (controlling the false discovery rate at 5%). Geometric mean ratios (GMR) and 95% CI were estimated exponentiating the regression parameters from the log-transformed outcome variable models. Odds ratios (OR) for detection of 6-CN and 95% CI were estimated exponentiating the regression parameters from the logistic regression models.

The first set of models were repeated in a sensitivity analysis that excluded the baseline sample as a fixed effect, and a second sensitivity analysis excluding two participants that followed the opposite order of treatment compared to the group their school was allocated. All analyses were performed in R (v.3.5) with RStudio (v.1.1.423) [29,30]. The statistical analysis plan and the input data can be found in the supporting information (S5 Text, S6 Text)

## Results and discussion

### Participant characteristics

Between October and December 2016, 12 public schools in the urban area of Limassol city were assessed for eligibility in the study and their headmasters were contacted. Six schools agreed to participate; three schools were randomly allocated to Group 1 (67 children) and the other three to Group 2 (124 children). In total, 24 children from Group 1 and 18 children from Group 2 who withdrew from the study 1–11 days after the beginning of the organic period and did not provide an organic period urine sample, were excluded from the data analysis. Baseline characteristics of the children who dropped out during days 1–11 were similar to the characteristics of children included in the main analysis (S1 Table). A total of 149 children were included in the main analysis with 43 children in Group 1 and 106 children in Group 2.

Overall, the sex distribution of the children was balanced (51% males), though a higher percentage of females was allocated in Group 2 (Table 1). The mean age was 11 years old and 89% completed 29–40 days of organic diet. A high level of education was reported for the participants’ parents, with the majority holding at least a university/college degree (82% for mothers and 65% for fathers). At baseline, most children had a normal weight (61%) with 38% being overweight or obese. The two groups differed in their consumption of specific foods during the conventional period. Specifically, children in Group 1 reported a lower consumption of meat, fish, eggs, nuts, and legumes (11 vs 17 portions per week), vegetables (4 vs 6 portions per week) and fats, sweets, and oils (25 vs 35 portions per week) than those in Group 2. The mean energy intake of the participants during the conventional period was estimated to be 2229 kcal, thus, higher than the reference dietary guidance value [average = 1976 kcal (2043 kcal for boys and 1908 kcal for girls)] calculated based on the EFSA average requirements for children of age 11 with moderate physical activity lifestyle [19].

**Table 1:** Demographics and baseline characteristics of the study population (overall and by group).

	Overall		Group 1		Group 2		
	Mean (SD) / Median [IQR]	N (%)	Mean (SD) / Median [IQR]	N (%)	Mean (SD) / Median [IQR]	N (%)	p-value^
N		149		43		106	
Sex							0.018
Female		73 (49.0)		14 (32.6)		59 (55.7)	
Male		76 (51.0)		29 (67.4)		47 (44.3)	
Age (years)	11.16 (0.59)		11.03 (0.53)		11.21 (0.61)		0.101
Mother’s education level							0.871
Master/PhD		41 (27.9)		13 (31.0)		28 (26.7)	
University/college		80 (54.4)		22 (52.4)		58 (55.2)	
Secondary		26 (17.7)		7 (16.7)		19 (18.1)	
Father’s education level							0.447
Master/PhD		41 (27.5)		15 (36.6)		26 (25.7)	
University/college		56 (37.6)		16 (39.0)		40 (39.6)	
Secondary		43 (28.9)		10 (24.4)		33 (32.7)	
Primary		2 (1.3)		0 (0.0)		2 (2.0)	
BMI-for-age at baseline*							0.114
Thinness		2 (1.4)		0 (0.0)		2 (1.9)	
Normal weight		90 (60.8)		23 (54.8)		67 (63.2)	
Overweight		36 (24.3)		9 (21.4)		27 (25.5)	
Obese		20 (13.5)		10 (23.8)		10 (9.4)	
BMI-for-age at the end of the organic diet treatment*							0.144
Thinness		2 (1.5)		0 (0.0)		2 (2.1)	
Normal weight		89 (67.4)		23 (62.2)		66 (69.5)	
Overweight		28 (21.2)		7 (18.9)		21 (22.1)	
Obese		13 (9.8)		7 (18.9)		6 (6.3)	
Waist circumference at baseline (cm)	69.00 [63.00, 77.00]		69.00 [66.50, 81.50]		69.00 [62.00, 76.00]		0.153
Days in organic period							0.589
12–21 days		12 (8.1)		4 (9.3)		8 (7.5)	
22–28 days		4 (2.7)		2 (4.7)		2 (1.9)	
29–40 days		133 (89.3)		37 (86.0)		96 (90.6)	
Number of samples provided (baseline sample included)							0.001
2		3 (2.0)		3 (7.0)		0 (0.0)	
3		3 (2.0)		3 (7.0)		0 (0.0)	
4		8 (5.4)		0 (0.0)		8 (7.5)	
5		3 (2.0)		1 (2.3)		2 (1.9)	
6		132 (88.6)		36 (83.7)		96 (90.6)	
Physical activity time** (hours/week)	4.00 [2.00, 6.00]		3.50 [1.75, 5.50]		4.00 [2.00, 6.00]		0.291
Sedentary activity time*** (hours/week)	19.00 [13.00, 28.00]		20.50 [14.50, 26.50]		16.40 [12.00, 28.00]		0.258
Milk products**** (portions/week)	15.85 (8.82)		15.85 (8.64)		15.85 (8.93)		0.999
Meat, fish, eggs, nuts, legumes**** (portions/week)	15.05 (7.31)		10.72 (4.46)		16.69 (7.52)		<0.001
Vegetables**** (portions/week)	5.38 (3.84)		3.78 (3.06)		5.98 (3.95)		0.003
Fruits**** (portions/week)	9.70 (6.69)		8.32 (5.33)		10.22 (7.09)		0.143
Cereals**** (portions/week)	21.78 (9.52)		19.45 (11.57)		22.66 (8.52)		0.081
Fats, sweets, oils**** (portions/week)	32.47 (17.70)		25.43 (11.27)		35.13 (18.96)		0.004

^the above variables were tested for differences between the two groups by χ^2^ tests for categorical variables, t-tests for normally distributed continuous variables and Wilcoxon tests for non-normally distributed continuous variables. These descriptive comparisons are simplified as they do not account for the clustering.

* Based on WHO 2007 cut-off points for BMI-for-age. BMI standard deviation scores taking in account age and sex were calculated and then based on the specific cut-offs, the BMI-for-age categories were created (<-2: Thinness; -2 < 1: Normal; >1: Overweight; >2: Obese)

** Summary of time spent in physical activities including hours per week spent on running, cycling, basketball, football, volleyball, swimming, dancing and other physical activities.

*** Summary of time spent in sedentary activities including hours per week spent on TV, computer, tablet, mobile phones, or other sedentary activities.

**** Food categories summarized based on consumption per week of food items belonging in each category as reported in the food frequency questionnaire for the conventional period of the study (food portion sizes were denoted in the questionnaire)–Food categories based on Children's Diet Pyramid for children aged 6–12 years (Ministry of Health, Cyprus)

### Follow-up and outcomes

The proportion of urine samples with 6-CN or 3-PBA values below LOD was higher in the organic period (77% and 32%, respectively) compared to the conventional period (66% and 15%, respectively). For 6-CN, aggregating both groups, a smaller percentage of samples had values above LOD by the end of organic treatment (23.4%) vs. the baseline (37.5%) (S1 Table). The percent change between the baseline and after 40 days of organic diet was highest for 3-PBA (-11.4%), followed by 8-OHdG (-1.7%), 8-iso-PGF2a (-1.6%), and MDA (-0.1%) (S1 Table). Median biomarker differences by treatment were significant for the pesticide biomarkers but not for the OSI biomarkers (S1 Table). More details about the biomarkers levels are available in S1 Table and S1 Fig. 

In regression models, during the organic diet treatment, participants in both groups had on average significantly lower levels of biomarkers of exposure to pyrethroids (3-PBA) (GMR = 0.297; 95% CI: 0.237, 0.373; Q<0.05) and the odds of being below the LOD of neonicotinoids (6-CN) was higher in the organic period (OR = 0.651; 95% CI: 0.463, 0.917; Q<0.05) (Table 2). Significantly lower levels of the OSI biomarker 8-OHdG (GMR = 0.888; 95% CI: 0.808, 0.976; Q<0.05) were also observed during the organic period (Table 2). A significant negative interaction between days of treatment and the dietary organic intervention was observed for 8-iso-PGF2a (β = -0.016; 95% CI: -0.023, -0.10; Q<0.05) and MDA (β = -0.005; 95% CI: -0.010, -0.001; Q<0.05), indicating a time-dependent reduction during the intervention period. The variance explained by school-level random intercepts was negligible (<0.001) for all (transformed) biomarkers of exposure and OSI (Table 2). Additional information on the p- and Q-values of the models can be found in S1 Table. The trends observed in the main analysis were retained also in the sensitivity analysis.

**Table 2:** Linear mixed-effect models of log-transformed pesticide metabolite 3-PBA and OSI biomarkers (8-OHdG, 8-iso-PGF2a, MDA) and logistic regression model of pesticide metabolite 6-CN (binary variable: Above and below LOD) as a function of time (# of days of treatment, time = 0 is start of treatment day), organic diet treatment (in comparison to the conventional diet treatment) and their interaction terms (if p<0.05), adjusting for the baseline levels of the compounds, and accounting for the repeated measurements and clustering by school.

	3-PBA (ng/g) Coefficient (95% CI)	8-OHdG (ug/g) Coefficient (95% CI)	8-iso-PGF2a (ng/g) Coefficient (95% CI)	MDA (nmol/g) Coefficient (95% CI)	6-CN (binary) ORs (95% CI)
Time	0.015 (0.005, 0.025)*	0 (-0.005, 0.004)	0.011 (0.005, 0.016)*	0.005 (0.002, 0.008)*	0.994 (0.978, 1.009)
Organic diet treatment	-1.214 (-1.44, -0.987)*	-0.119 (-0.213, -0.024)*	0.408 (0.232, 0.584)*	0.189 (0.083, 0.295)*	0.651 (0.463, 0.917)*
Interaction of Organic Diet* Time			-0.016 (-0.023, -0.010)*	-0.005 (-0.01, -0.001)*	
Number of samples	705	534	649	705	705
Number of participants	149	114	144	149	149
Number of schools	6	6	6	6	6
Participant-level random intercept variance	0.223	0.030	0.026	0.006	0.111
School-level random intercept variance	<0.0001	<0.0001	0.005	0.004	0.008
Residual variance	2.260	0.295	0.219	0.087	1
ICC _PARTICIPANT/SCHOOL_	0.09	0.10	0.10	0.06	0.03

Q-value: Benjamini-Hochberg (BH) adjusted p-value

*Q-value <0.05

Models details:

(a) 3-PBA, 8-OHdG, 8-iso-PGF2a and MDA are creatinine adjusted and log-transformed

(b) 6-CN is used as a binary variable in a logistic regression model with two levels: above and below LOD (below LOD is the reference)

(c) Adjusted for baseline levels of the dependent variable.

(d) Random intercepts for the repeated visits within participants, and the participants nested within schools, with unstructured covariance matrix.

Abbreviations: 3-PBA: 3-phenoxybenzoic acid; 8-OHdG: 8-hydroxy-2′-deoxyguanosine; 8-iso-PGF2a: 8-iso-Prostaglandin F2a; MDA: malondialdehyde; 6-CN: 6-chloronicotinic acid; CI: confidence interval; ORs: odds ratios

In the linear mixed effect models of the OSI biomarkers, a statistically significant (Q<0.05) association was found between the pesticide metabolite (3-PBA) and 8-OHdG (GMR = 1.064; 95% CI: 1.033, 1.095) or 8-iso-PGF2a (GMR = 1.058; 95% CI: 1.035, 1.081), but not with MDA (Table 3); the 6-CN was not found to be associated with the levels of the OSI biomarkers. The organic diet was negatively associated with age-and-sex-standardized BMI z-scores (β = -0.131; 95% CI: -0.179, -0.083; Q<0.001) (Table 4).

**Table 3:** Linear mixed-effect models of log-transformed OSI biomarkers levels (8-OHdG, 8-iso-PGF2a, MDA) regressed on the levels of the pesticide biomarkers (3-PBA & 6-CN) and time and adjusted for age, sex and baseline levels of the dependent variable, accounting for the repeated measurements and clustering by school (interaction terms were not significant).

	8-OHdG (ug/g) Coefficient (95% CI)	8-iso-PGF2a (ng/g) Coefficient (95% CI)	MDA (nmol/g) Coefficient (95% CI)	8-OHdG (ug/g) Coefficient (95% CI)	8-iso-PGF2a (ng/g) Coefficient (95% CI)	MDA (nmol/g) Coefficient (95% CI)
Time	-0.001 (-0.006, 0.003)	-0.001 (-0.004, 0.003)	0.001 (-0.001, 0.003)	-0.001 (-0.005, 0.003)	0 (-0.003, 0.004)	0.002 (0, 0.004)
3-PBA (ng/g)	0.062 (0.032, 0.091)*	0.056 (0.034, 0.078)*	0.005 (-0.009, 0.018)			
6-CN > LOD (binary)				0.1 (-0.009, 0.209)	0.006 (-0.082, 0.093)	-0.013 (-0.065, 0.039)
Number of samples	533	648	704	533	648	704
Number of participants	113	143	148	113	143	148
Number of schools	6	6	6	6	6	6
Participant-level random intercept variance	0.026	0.022	0.006	0.030	0.024	0.006
School-level random intercept variance	<0.0001	0.003	0.003	<0.0001	0.002	0.003
Residual variance	0.292	0.221	0.090	0.298	0.229	0.090
ICC _PARTICIPANT/SCHOOL_	0.08	0.09	0.06	0.09	0.09	0.06

Q-value: Benjamini-Hochberg (BH) adjusted p-value

*Q-value ≤0.003

Models details:

(a) 3-PBA, 8-OHdG, 8-iso-PGF2a and MDA are creatinine adjusted and log-transformed

(b) 6-CN is used as a binary variable with two levels: above and below LOD (below LOD is the reference)

(c) Adjusted for age, sex and baseline levels of the dependent variable.

(d) Random intercepts for the repeated visits within participants, and participants nested within schools, with unstructured covariance matrix.

Abbreviations: 3-PBA: 3-phenoxybenzoic acid; 8-OHdG: 8-hydroxy-2′-deoxyguanosine; 8-iso-PGF2a: 8-iso-prostaglandin F2a; MDA: malondialdehyde; 6-CN: 6-chloronicotinic acid; CI: confidence interval

**Table 4:** Difference in mean age-and-sex-standardized BMI z-scores by organic diet intervention, in models with or without further adjustment for log-transformed, creatinine-corrected biomarker of either exposure (pesticides) or effect (OSI), accounting for repeated measures and clustering by school.

	—————————————————-BMI z-score coefficient (95% CI)——————————————————————-					
Organic diet treatment	-0.131 (-0.179, -0.083)*	-0.13 (-0.183, -0.076)*	-0.121 (-0.17, -0.071)*	-0.133 (-0.181, -0.084)*	-0.095 (-0.153, -0.037)*	-0.128 (-0.181, -0.076)*
3-PBA (ng/g)		0.001 (-0.023, 0.026)				
6-CN–above LOD (binary)			0.059 (-0.014, 0.132)			
MDA (nmol/g)				-0.043 (-0.162, 0.075)		
8-OHdG (ug/g)					0.027 (-0.05, 0.103)	
8-iso-PGF2a (ng/g)						0.023 (-0.065, 0.111)
Number of measurements	265	265	265	265	234	252
Number of participants	133	133	133	133	133	133
Number of schools	6	6	6	6	6	6
Participant-level random intercept variance	1.263	1.264	1.268	1.264	1.256	1.260
School-level random intercept variance	0.083	0.083	0.084	0.082	0.081	0.082
Residual variance	0.040	0.040	0.039	0.040	0.040	0.042
ICC _PARTICIPANT/SCHOOL_	0.91	0.91	0.91	0.91	0.91	0.91

Q-value: Benjamini-Hochberg (BH) adjusted p-value

*Q-value ≤0.006

Models details:

(a) 3-PBA, 8-OHdG, 8-iso-PGF2a and MDA are creatinine adjusted and log-transformed

(b) 6-CN is used as a binary variable with two levels: above and below LOD (below LOD is the reference)

(c) Random intercepts for the repeated visits within participants, and the participants nested within schools; with unstructured covariance matrix.

Abbreviations: 8-OHdG: 8-hydroxy-2′-deoxyguanosine; 8-iso-PGF2a: 8-iso-prostaglandin F2a; MDA: malondialdehyde; 6-CN: 6-chloronicotinic acid; CI: confidence interval

In this cluster-randomized crossover trial, data showed that the organic diet treatment reduced the body burden of the biomarkers of exposure to pyrethroids (3-PBA) and neonicotinoids (6-CN). The observed trends were consistent with literature on the impact of organic diet in reducing the magnitude of biomarkers of exposure to organophosphorus pesticides [31–33]. Data also showed that there was an immediate and sustained reduction in 8-OHdG during the organic period, and, after an initial increase, a gradual reduction during the organic period of 8-iso-PGF2a and MDA (S1 Table, S1 Fig). The fact that the 6-CN was not detected in most of the samples (72% of samples <LOD) was expected given the restrictions introduced at the EU level in 2013 against certain neonicotinoid pesticides [34]. Our decision to randomize the intervention at the school-cluster level was intended to avoid the transfer of knowledge about the organic diet intervention from children randomized in the intervention arm to children randomized in the conventional arm (contamination effect) [35]. Compliance was reported to be 90% or higher (S1 Table), perhaps from a peer pressure effect as all children at a given school were assigned were following the same intervention. However, we cannot exclude the participants’ reporting bias. The findings did not change when we excluded the two children who did not comply with their school’s assigned randomization schedule but started the organic treatment with their classmates. A single, organic foods, certified restaurant was responsible for the preparation and provision of organic meals to all schools during the organic period, eliminating differences in cooking preparation options and cooking quality. As such, the same raw products, preparation of foods, delivery and consumption of organic meals was followed by all participants. The risk of bias in intervention assignment was minimized using central randomization. Thus, the results, i.e. reduction of pesticide biomarkers and impact of organic diet on OSI biomarkers can be considered generalizable for the specific population (i.e. primary school children residing in an urban area following the Cypriot diet). Given that participating schools were randomly selected from various areas of the city of Limassol, we do not expect that background population characteristics such as neighborhoods’ socioeconomic backgrounds have influenced the observed data.

A strength of the current trial is the larger sample size in comparison to previous trials on the effect of organic diet on OSI biomarkers and antioxidant capacity. Most studies included ≤ 40 participants and only one study had 130 participants [11]. Additionally, this study covers a large intervention duration (up to 40 days).

Limitations include the fact that the reported compliance may not reflect the actual compliance of the children to the organic diet, as children could either not consume all meal portion, or families could provide them with extra organic food items, if needed. The organic dietary treatment was a behavioral intervention that may have had other changes beyond the intended pesticide exposure reduction; one example could be the possible caloric imbalance between the two periods. Another example is that, one fruit and three portions of vegetables per day were provided during the organic period which is higher than what the participants had mentioned they consumed regularly during the conventional period (estimated to be 28 portions vs 15 portions). Overall, participants might have changed their habits during their participation as they were initially informed about organic diet and the value of healthy lifestyle.

In conclusion, in this trial, a systematic organic dietary intervention program followed for up to 40 days by healthy children recruited from primary schools was found to reduce biomarkers of exposure to pesticides (3-PBA and 6-CN), and over time, all measured OSI biomarkers (8-iso-PGF2a, 8-OHdG and MDA). Age- and sex-standardized BMI z-scores were reduced at the end of the organic diet scheme, but inferences regarding the potential benefits of organic diet for OSI and obesity are limited by the confounding of this organic diet intervention with caloric intake reduction and change in lifestyle (i.e., increased consumption of organic fruits and vegetables). Our findings are consistent with a possible mediating pathway of pesticide exposure reductions leading to decreased oxidative stress/inflammation and consequently lower BMI, but this was not causal and other studies are needed to investigate this hypothesis further and exclude alternative explanations.

## Supporting information

S1 Fig Biomarker box plots. (DOCX)

S1 Table Biomarker data trends. (DOCX)

S1 Text CONSORT checklist-study protocol. (DOCX)

S2 Text Food diary documentation. (DOCX)

S3 Text Study questionnaires. (PDF)

S4 Text Bioanalytical protocols. (DOCX)

S5 Text Statistical analysis plan. (DOCX)

S6 Text Scripts and input (the input data files contain pseudo birth dates). (ZIP)

## Abbreviations

GMR: geometric mean ratio
OSI: oxidative stress and inflammation
MDA: malondialdehyde
iso-PGF2a: 8-iso-15(S)-Prostaglandin F2α
8-OHdG: 8-oxo-2'-deoxyguanosine
BMI: body mass index
BH: Benjamini-Hoechberg
3-PBA: 3-phenoxybenzoic acid
6-CN: 6-chloronicotinic acid
