# Supplementary material for: A cluster-randomized crossover trial of organic diet impact on biomarkers of exposure to pesticides and biomarkers of oxidative stress/inflammation in primary school children
Source: PLoS One. 2019 Sep 4;14(9):e0219420. doi: 10.1371/journal.pone.0219420 (PMC6726134; doi:10.1371/journal.pone.0219420)
Supplement: S1 Fig — (DOCX) [file pone.0219420.s001.docx]

S1 Figure

A cluster-randomized crossover trial of organic diet for primary school children

*Konstantinos C. Makris^1*^, PhD, Corina Konstantinou^1^, MS, Xanthi D. Andrianou^1^, MS, Pantelis Charisiadis^1^, PhD, Alexis Kyriacou^2^, RD, Matthew O. Gribble^3,4^^, PhD DABT, and Costas A. Christophi^1^^, PhD*

^1^ Cyprus International Institute for Environmental and Public Health, Cyprus University of Technology, Limassol, Cyprus

^2^ Faculty of Health Sciences and Sport, University of Stirling, Stirling, UK

^3^ Department of Environmental Health, Emory University, Atlanta, GA, USA

^4^ Department of Epidemiology, Emory University, Atlanta, GA, USA

^ Both authors contributed equally to this work.

* Corresponding author: Konstantinos Christos Makris, Associate Professor of Environmental Health, Cyprus International Institute for Environmental and Public Health, School of Health Sciences, Cyprus University of Technology, Limassol, Cyprus.

Phone: 357-25002398, FAX: 357-25002676

E-mail: [konstantinos.makris@cut.ac.cy](mailto:konstantinos.makris@cut.ac.cy) (KCM)

| 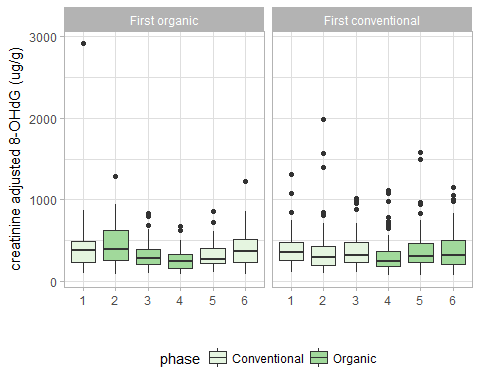 |
| --- |
| 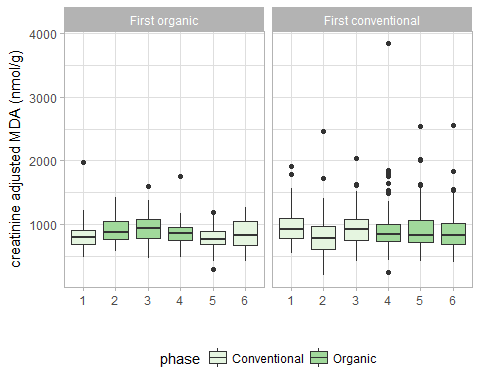 |
| 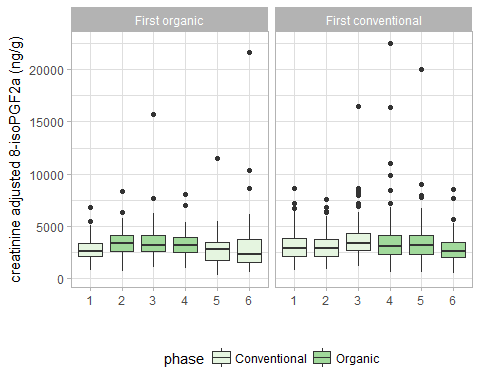 |

S1 Figure. Boxplots of the creatinine adjusted OSI biomarkers by group (left: Group 1; right: Group 2).
