# Supplementary material for: A cluster-randomized crossover trial of organic diet impact on biomarkers of exposure to pesticides and biomarkers of oxidative stress/inflammation in primary school children
Source: PLoS One. 2019 Sep 4;14(9):e0219420. doi: 10.1371/journal.pone.0219420 (PMC6726134; doi:10.1371/journal.pone.0219420)
Supplement: S1 Table — (DOCX) [file pone.0219420.s002.docx]

Additional Tables

A cluster-randomized crossover trial of organic diet for primary school children

*Konstantinos C. Makris^1*^, PhD, Corina Konstantinou^1^, MS, Xanthi D. Andrianou^1^, MS, Pantelis Charisiadis^1^, PhD, Alexis Kyriacou^2^, RD, Matthew O. Gribble^3,4^^, PhD DABT, and Costas A. Christophi^1^^, PhD*

^1^ Cyprus International Institute for Environmental and Public Health, Cyprus University of Technology, Limassol, Cyprus

^2^ Faculty of Health Sciences and Sport, University of Stirling, Stirling, UK

^3^ Department of Environmental Health, Emory University, Atlanta, GA, USA

^4^ Department of Epidemiology, Emory University, Atlanta, GA, USA

^ Both authors contributed equally to this work.

* Corresponding author: Konstantinos Christos Makris, Associate Professor of Environmental Health, Cyprus International Institute for Environmental and Public Health, School of Health Sciences, Cyprus University of Technology, Limassol, Cyprus.

Phone: 357-25002398, FAX: 357-25002676

E-mail: [konstantinos.makris@cut.ac.cy](mailto:konstantinos.makris@cut.ac.cy) (KCM)

##

## Tables

S1 Table Demographics and baseline characteristics of children not included in the main data analysis (drop outs; staying <12 days in the organic period) and of those children included in the main data analysis (Active participants; ≥12 days in the organic period).

|  | Dropouts | | Active participants | |  |
| --- | --- | --- | --- | --- | --- |
|  | Mean (SD)/ Median [IQR] | N (%) | Mean (SD)/ Median [IQR] | N (%) | p-value^ |
| N |  | 42 |  | 149 |  |
| Sex |  |  |  |  | 0.177 |
| Female |  | 15 (35.7) |  | 73 (49.0) |  |
| Male |  | 27 (64.3) |  | 76 (51.0) |  |
| Age (years) | 11.10 (0.87) |  | 11.16 (0.59) |  | 0.685 |
| Group |  |  |  |  | 0.001 |
| Group 1 |  | 24 (57.1) |  | 43 (28.9) |  |
| Group 2 |  | 18 (42.9) |  | 106 (71.1) |  |
| Mother’s education level |  |  |  |  | 0.179 |
| Master/PhD |  | 4 (12.5) |  | 41 (27.9) |  |
| University/college |  | 22 (68.8) |  | 80 (54.4) |  |
| Secondary |  | 6 (18.8) |  | 26 (17.7) |  |
| Father’s education level |  |  |  |  | 0.282 |
| Master/PhD |  | 6 (18.8) |  | 41 (28.9) |  |
| University/college |  | 18 (56.2) |  | 56 (39.4) |  |
| Secondary |  | 7 (21.9) |  | 43 (30.3) |  |
| Primary |  | 1 (3.1) |  | 2 (1.4) |  |
| Weight status* |  |  |  |  | 0.743 |
| Thinness |  | 1 (4.2) |  | 2 (1.4) |  |
| Normal weight |  | 13 (54.2) |  | 90 (60.8) |  |
| Overweight |  | 6 (25.0) |  | 36 (24.3) |  |
| Obese |  | 4 (16.7) |  | 20 (13.5) |  |
| Waist circumference (cm) | 70.00 [63.00, 74.50] |  | 69.00 [63.00, 77.00] |  | 0.719 |
| Physical activity time** (hours/week) | 2.00 [0.00, 3.00] |  | 4.00 [2.00, 6.00] |  | <0.001 |
| Sedentary activity time*** (hours/week) | 18.00 [9.93, 26.00] |  | 19.00 [13.00, 28.00] |  | 0.332 |

^the above variables were tested for differences between the two groups by chi-square tests for categorical variables, t-tests for normally distributed continuous variables and Wilcoxon tests for non-normally distributed continuous variables

*Based on WHO 2007 cut-off points for BMI-for-age. BMI standard deviation scores taking in account age and sex were calculated and then based on specific cut-offs the BMI-for-age categories were created (<-2: Thinness, 1>-1: Normal >1: Overweight >2: Obese)

** Summary of time spent in physical activities including hours per week spent on running, cycling, basketball, football, volleyball, swimming, dancing and other physical activities.

*** Summary of time spent in sedentary activities including hours per week spent on TV, computer, tablet, mobile and other sedentary activities.

S2 Table Distribution of 6-CN values above LOD by group and by sample

|  | Group 1 | Group 2 |
| --- | --- | --- |
|  | N (%) | N (%) |
| Sample 1 | 16 (37.2) | 40 (37.7) |
| Sample 2 | 14 (33.3) | 32 (30.2) |
| Sample 3 | 8 (20.0) | 30 (28.3) |
| Sample 4 | 10 (27.0) | 24 (22.6) |
| Sample 5 | 9 (24.3) | 22 (22.4) |
| Sample 6 | 19 (51.4) | 19 (19.8) |

Abbreviations: 6-CN: 6-chloronicotinic acid. LOD=0.075 ug/L.

Samples 2, 3, 4 for Group 1 and samples 4, 5, 6 for Group 2 were taken during the organic period and the rest samples, during the conventional period.

S3 Table Percent change of last conventional sample to last organic sample

|  | % change [95% C1] | p-value |
| --- | --- | --- |
| 3-PBA (ng/g creatinine) | 11.4 [5.7, 17.2 ] | 0.000 |
| MDA (nmol/g creatinine) | 0.1 [-1.1, 1.2] | 0.913 |
| 8-OHdG (μg/g creatinine) | 1.7 [-0.7, 4.0] | 0.167 |
| 8-iso-PGF2a (ng/g creatinine) | 1.6 [0.2, 2.9] | 0.023 |

All variables are creatinine-adjusted and log-transformed.

Based on the imputed data below LOD for 3-PBA & MDA

Abbreviations: 3-PBA: 3-phenoxybenzoic acid; MDA: malondialdehyde; 8-OHdG:

8-hydroxy-2′-deoxyguanosine; 8-iso-PGF2a: 8-iso-Prostaglandin F2a.

S4 Table Comparison of the medians of the pesticide metabolites and the biomarkers of oxidative stress/inflammation during the conventional treatment.

|  | Conventional | Organic |  |
| --- | --- | --- | --- |
|  | Median [25^th^-75^th^ percentile] | Median [25^th^-75^th^ percentile] | p-value |
| n | 435 | 419 |  |
| 6-CN (ng/g creatinine) | 26.32 [6.47, 180.38] | 19.50 [5.45, 79.46] | 0.017 |
| 3-PBA (ng/g creatinine) | 2088.35 [688.88, 5273.25] | 575.76 [164.14, 1681.08] | <0.001 |
| MDA (μmol/g creatinine) | 858.21 [691.40, 1016.46] | 846.15 [724.58, 1029.20] | 0.338 |
| 8-OHdG (μg/g creatinine) | 322.60 [222.02, 471.06] | 295.74 [202.04, 446.74] | 0.070 |
| 8-iso-PGF2a (ng/g creatinine) | 2963.74 [2112.35, 3871.45] | 3028.44 [2299.91, 3955.47] | 0.259 |

All variables are creatinine-adjusted. Based on the imputed data below LOD for 6-CN, 3-PBA & MDA

Abbreviations: 6-CN: 6-chloronicotinic acid; 3-PBA: 3-phenoxybenzoic acid; MDA: malondialdehyde;

8-OHdG: 8-hydroxy-2′-deoxyguanosine; 8-iso-PGF2a: 8-iso-Prostaglandin F2a.

S5 Table Percentiles of the pesticide metabolites and the biomarkers of oxidative stress/inflammation for the whole study period.

|  | min | 5th | 25th | 50th | 75th | 95th | max |
| --- | --- | --- | --- | --- | --- | --- | --- |
| 6-CN (ng/g creatinine) | 0.02 | 0.78 | 5.92 | 21.9 | 131.6 | 1003 | 22158 |
| 3-PBA (ng/g creatinine) | 4.77 | 51.60 | 292.6 | 1169 | 3283 | 12580 | 59515 |
| MDA (nmol/g creatinine) | 189 | 526 | 708 | 852 | 1025 | 1410 | 3846 |
| 8-OHdG (μg/g creatinine) | 69.4 | 116.7 | 212.3 | 309 | 456 | 801 | 2913 |
| 8-iso-PGF2a (ng/g creatinine) | 261 | 1332 | 2229 | 2981 | 3912 | 6320 | 22508 |

All variables are creatinine-adjusted. Based on the imputed data below LOD for 6-CN, 3-PBA & MDA

Abbreviations: 6-CN: 6-chloronicotinic acid; 3-PBA: 3-phenoxybenzoic acid; MDA: malondialdehyde;

8-OHdG: 8-hydroxy-2′-deoxyguanosine; 8-iso-PGF2a: 8-iso-Prostaglandin F2a.

S6 Table Percentiles of the pesticide metabolites and the biomarkers of oxidative stress/inflammation during the organic treatment.

|  | min | 5th | 25th | 50th | 75th | 95th | max |
| --- | --- | --- | --- | --- | --- | --- | --- |
| 6-CN (ng/g creatinine) | 0.02 | 0.66 | 5.45 | 19.5 | 79.5 | 680 | 13761 |
| 3-PBA (ng/g creatinine) | 4.77 | 45.03 | 164.14 | 575.8 | 1681 | 7527 | 42484 |
| MDA (nmol/g creatinine) | 237 | 553 | 725 | 846 | 1029 | 1485 | 3846 |
| 8-OHdG (μg/g creatinine) | 69 | 108 | 202 | 296 | 447 | 800 | 1576 |
| 8-iso-PGF2a (ng/g creatinine) | 447 | 1321 | 2300 | 3028 | 3955 | 6248 | 22508 |

All variables are creatinine-adjusted. Based on the imputed data below LOD for 6-CN, 3-PBA & MDA

Abbreviations: 6-CN: 6-chloronicotinic acid; 3-PBA: 3-phenoxybenzoic acid; MDA: malondialdehyde;

8-OHdG: 8-hydroxy-2′-deoxyguanosine; 8-iso-PGF2a: 8-iso-Prostaglandin F2a.

S7 Table Percentiles of the pesticide metabolites and the biomarkers of oxidative stress/inflammation during the conventional treatment.

|  | min | 5th | 25th | 50th | 75th | 95th | max |
| --- | --- | --- | --- | --- | --- | --- | --- |
| 6-CN (ng/g creatinine) | 0.09 | 0.87 | 6.47 | 26.3 | 180.4 | 1408 | 22158 |
| 3-PBA (ng/g creatinine) | 11.47 | 78.93 | 688.9 | 2088 | 5273 | 15224 | 59515 |
| MDA (nmol/g creatinine) | 189 | 508 | 691 | 858 | 1016 | 1355 | 2462 |
| 8-OHdG (μg/g creatinine) | 83.7 | 127.2 | 222 | 322.6 | 471 | 796 | 2913 |
| 8-iso-PGF2a (ng/g creatinine) | 260.7 | 1339 | 2112 | 2964 | 3871 | 6320 | 21642 |

All variables are creatinine-adjusted. Based on the imputed data below LOD for 6-CN, 3-PBA & MDA

Abbreviations: 6-CN: 6-chloronicotinic acid; 3-PBA: 3-phenoxybenzoic acid; MDA: malondialdehyde;

8-OHdG: 8-hydroxy-2′-deoxyguanosine; 8-iso-PGF2a: 8-iso-Prostaglandin F2a.

S8 Table P-values and Q-values of the models’ parameters

| Model | Fixed Effects | p-value | Q-value |
| --- | --- | --- | --- |
| 3-PBA ~ Time (days) + Organic diet | Organic diet | 0.000 | 0.000 |
| 8-iso-PGF2a ~ Time (days) + Organic diet + Time*Organic diet | Time (days) | 0.000 | 0.000 |
| 8-iso-PGF2a ~ Time (days) + Organic diet + Time*Organic diet | Time*Organic diet | 0.000 | 0.000 |
| 8-iso-PGF2a ~ Time (days) + Organic diet + Time*Organic diet | Baseline 8-iso-PGF2a levels | 0.000 | 0.000 |
| 8-iso-PGF2a ~ Time (days) + Organic diet + Time*Organic diet | Organic diet | 0.000 | 0.000 |
| MDA ~ Time (days) + Organic diet + Time*Organic diet | Baseline MDA levels | 0.000 | 0.000 |
| 8-OHdG ~ 3-PBA + Time (days) | 3-PBA | 0.000 | 0.000 |
| 8-iso-PGF2a ~ 3-PBA + Time (days) | 3-PBA | 0.000 | 0.000 |
| BMI ~ Organic diet | Organic diet | 0.000 | 0.000 |
| BMI ~ Organic diet + 3-PBA | Organic diet | 0.000 | 0.000 |
| BMI ~ Organic diet + 6-CN | Organic diet | 0.000 | 0.000 |
| BMI ~ Organic diet + MDA | Organic diet | 0.000 | 0.000 |
| BMI ~ Organic diet + 8-iso-PGF2a | Organic diet | 0.000 | 0.000 |
| MDA ~ Time (days) + Organic diet + Time*Organic diet | Organic diet | 0.001 | 0.003 |
| 8-iso-PGF2a ~ 3-PBA + Time (days) | Baseline 8-iso-PGF2a levels | 0.001 | 0.003 |
| MDA ~ 3-PBA + Time (days) | Baseline MDA levels | 0.001 | 0.003 |
| 8-iso-PGF2a ~ 6-CN + Time (days) | Baseline 8-iso-PGF2a levels | 0.001 | 0.003 |
| MDA ~ 6-CN + Time (days) | Baseline MDA levels | 0.001 | 0.003 |
| 8-OHdG ~ Time (days) + Organic diet | Baseline 8-OhdG levels | 0.002 | 0.006 |
| BMI ~ Organic diet + 8-OHdG | Organic diet | 0.002 | 0.006 |
| MDA ~ Time (days) + Organic diet + Time*Organic diet | Time (days) | 0.003 | 0.008 |
| 3-PBA ~ Time (days) + Organic diet | Time (days) | 0.004 | 0.011 |
| 8-OHdG ~ 3-PBA + Time (days) | Baseline 8-OHdG levels | 0.008 | 0.020 |
| MDA ~ Time (days) + Organic diet + Time*Organic diet | Time*Organic diet | 0.010 | 0.024 |
| 8-OHdG ~ 6-CN + Time (days) | Baseline 8-OHdG levels | 0.011 | 0.026 |
| 8-OHdG ~ Time (days) + Organic diet | Organic diet | 0.014 | 0.030 |
| 6-CN ~ Time (days) + Organic diet | Organic diet | 0.014 | 0.030 |
| 3-PBA ~ Time (days) + Organic diet | Baseline 3-PBA levels | 0.019 | 0.039 |
| 8-OHdG ~ 6-CN + Time (days) | 6-CN >LOD | 0.072 | 0.144 |
| BMI ~ Organic diet + 6-CN | 6-CN >LOD | 0.117 | 0.226 |
| MDA ~ 6-CN + Time (days) | Time (days) | 0.131 | 0.245 |
| 8-iso-PGF2a ~ 6-CN + Time (days) | Sex - Female | 0.146 | 0.260 |
| MDA ~ 3-PBA + Time (days) | Time (days) | 0.148 | 0.260 |
| 8-iso-PGF2a ~ 3-PBA + Time (days) | Sex - Female | 0.205 | 0.350 |
| 8-iso-PGF2a ~ 6-CN + Time (days) | Age | 0.335 | 0.538 |
| 8-OHdG ~ 3-PBA + Time (days) | Age | 0.339 | 0.538 |
| 8-iso-PGF2a ~ 3-PBA + Time (days) | Age | 0.343 | 0.538 |
| 6-CN ~ Time (days) + Organic diet | Time (days) | 0.414 | 0.632 |
| 8-OHdG ~ 6-CN + Time (days) | Age | 0.425 | 0.632 |
| BMI ~ Organic diet + MDA | MDA | 0.474 | 0.665 |
| MDA ~ 3-PBA + Time (days) | 3-PBA | 0.480 | 0.665 |
| 8-OHdG ~ 3-PBA + Time (days) | Time (days) | 0.490 | 0.665 |
| BMI ~ Organic diet + 8-OHdG | 8-OHdG | 0.493 | 0.665 |
| MDA ~ 6-CN + Time (days) | Sex - Female | 0.520 | 0.685 |
| MDA ~ 3-PBA + Time (days) | Sex - Female | 0.535 | 0.690 |
| 8-OHdG ~ 6-CN + Time (days) | Sex - Female | 0.550 | 0.690 |
| MDA ~ 3-PBA + Time (days) | Age | 0.570 | 0.690 |
| MDA ~ 6-CN + Time (days) | Age | 0.571 | 0.690 |
| BMI ~ Organic diet + 8-iso-PGF2a | 8-iso-PGF2a | 0.607 | 0.718 |
| MDA ~ 6-CN + Time (days) | 6-CN >LOD | 0.624 | 0.724 |
| 8-OHdG ~ 6-CN + Time (days) | Time (days) | 0.649 | 0.738 |
| 8-iso-PGF2a ~ 3-PBA + Time (days) | Time (days) | 0.665 | 0.742 |
| 8-OHdG ~ Time (days) + Organic diet | Time (days) | 0.816 | 0.878 |
| 8-OHdG ~ 3-PBA + Time (days) | Sex - Female | 0.817 | 0.878 |
| 8-iso-PGF2a ~ 6-CN + Time (days) | Time (days) | 0.883 | 0.923 |
| 8-iso-PGF2a ~ 6-CN + Time (days) | 6-CN >LOD | 0.896 | 0.923 |
| BMI ~ Organic diet + 3-PBA | 3-PBA | 0.908 | 0.923 |
| 6-CN ~ Time (days) + Organic diet | Baseline 6-CN >LOD | 0.923 | 0.923 |

Q-value: Benjamini-Hochberg (BH) adjusted p-value

Adjustments for models reported in the main manuscript.
