# Supplementary material for: A cluster-randomized crossover trial of organic diet impact on biomarkers of exposure to pesticides and biomarkers of oxidative stress/inflammation in primary school children
Source: PLoS One. 2019 Sep 4;14(9):e0219420. doi: 10.1371/journal.pone.0219420 (PMC6726134; doi:10.1371/journal.pone.0219420)
Supplement: S1 Text — (DOCX) [file pone.0219420.s003.docx]

Study Protocol

**CONSORT Checklist of Items for Reporting Cluster Randomized Trials of NPTs**

A cluster-randomized crossover trial of organic diet for primary school children

*Konstantinos C. Makris^1*^, PhD, Corina Konstantinou^1^, MS, Xanthi D. Andrianou^1^, MS, Pantelis Charisiadis^1^, PhD, Alexis Kyriacou^2^, RD, Matthew O. Gribble^3,4^^, PhD DABT, and Costas A. Christophi^1^^, PhD*

^1^ Cyprus International Institute for Environmental and Public Health, Cyprus University of Technology, Limassol, Cyprus

^2^ Faculty of Health Sciences and Sport, University of Stirling, Stirling, UK

^3^ Department of Environmental Health, Emory University, Atlanta, GA, USA

^4^ Department of Epidemiology, Emory University, Atlanta, GA, USA

^ Both authors contributed equally to this work.

* Corresponding author: Konstantinos Christos Makris, Associate Professor of Environmental Health, Cyprus International Institute for Environmental and Public Health, School of Health Sciences, Cyprus University of Technology, Irinis 95, Limassol, Cyprus.

Phone: 357-25002398, FAX: 357-25002676

E-mail: [konstantinos.makris@cut.ac.cy](mailto:konstantinos.makris@cut.ac.cy) (KCM)

## CONSORT Checklist of Items for Reporting Cluster Randomized Trials of NPTs

| **Section** | **Item** | | | **CONSORT Description** | | | | | | | | **Extension for NPT Trials & cluster randomised trials**^1,2^ | | | | | |  | | |  |  |  |  |
| --- | --- | --- | --- | --- | --- | --- | --- | --- | --- | --- | --- | --- | --- | --- | --- | --- | --- | --- | --- | --- | --- | --- | --- | --- |
| **Title and abstract** | | | | | | | | | | | | | | | | | |  | | |  |  |  |  |
|  | 1a | | | Identification as a randomized trial in the title | | | | | | | |  | | | | | |  | | |  |  |  |  |
|  | 1b | | | Structured summary of trial design, methods, results, and conclusions | | | | | | | | Refer to CONSORT extension for abstracts for NPT trials | | | | | |  | | |  |  |  |  |
| The title is A cluster-randomized crossover trial of organic diet for primary school children. The abstract can be found at the beginning of the manuscript. | | | | | | | | | | | | | | | | | | | | | |  |  |  |
| **Introduction** | | | | | | | | | | | | | | | | | | | | | |  |  |  |
| Background and Objectives | | 2a | | | Scientific background and explanation of rationale | | | | | | | | Rationale for using a cluster design | | | | | | | | |  |  |  |
|  | |  | | | Specific objectives or hypotheses | | | | | | | | Whether objectives pertain to the cluster level, the individual participant level or both | | | | | | | | |  |  |  |
| The background and objectives are described at the “Introduction” section of the manuscript.  The rationale of using a cluster design is described in the Methods part below (Item 3a). | | | | | | | | | | | | | | | | | | | | | |  |  |  |
| **Methods** |  | | |  | | | | | | | |  | | | | | | |  | | | | |  |
| Trial design | 3a | | | Description of trial design (such as parallel, factorial) including allocation ratio | | | | | | | | When applicable, how care providers were allocated to each trial group | | | | | | | | | |  |  |  |
|  | 3b | | | Important changes to methods after trial commencement (such as eligibility criteria), with reasons | | | | | | | | Definition of cluster and description of how the design features apply to the clusters: | | | | | | | | | |  |  |  |
| A 2 x 2 cluster-randomized cross-over trial at subject level was conducted to evaluate the effect of a 40-day organic diet compared to a 40-day conventional diet on biomarkers of exposure (pesticides metabolites) and biomarkers of effect (oxidative stress/inflammation markers) in children. The study was conducted in six randomly selected primary schools (clusters) with two periods (organic and conventional) in Limassol, Cyprus between January and April 2017. The trial was approved by the Cyprus National Bioethics Committee (ΕΕΒΚ/ΕΠ/2016/25) and the Cyprus Ministry of Education and Culture (7.15.06.15/2). The schools were *a priori* randomized to one of two groups differing only on the sequence of the treatments; organic diet -conventional diet (Group 1) and conventional diet-organic diet (Group 2). The ratio of Group 1: Group 2 participants was 1:2.5. | | | | | | | | | | | | | | | | | | | | | |  |  |  |
| The reasons for this cluster randomization were: (1) to avoid the transfer of knowledge within schools about the organic diet intervention from children randomised in the intervention arm to children randomised in the conventional arm (contamination effect); (2) to enhance compliance, since all participating children at a given school would follow the same intervention; (3) more efficient delivery of the organic meals during the organic period since the organic meals-preparing restaurant would daily deliver meals to 3 instead of 6 schools. | | | | | | | | | | | | | | | | | | | | | |  |  |  |
| The reasons for the crossover design were: (1) each participant serves as his/her own control and the influence of confounding variables (i.e., variables that are imbalanced between the treatment and control groups) is reduced; (2) repeated measures over a longitudinal study reduce the chance for a study finding to be attributable to chance under temporal measurement error. One organic-certified restaurant was responsible for the preparation and provision of organic meals for all schools during the organic period helping ensure that the treatment was comparable per the stable unit treatment value assumption (SUTVA) allowing us to draw meaningful inference about the effect of organic diet. Informed consent was obtained at school level by the headmaster of the participating school, followed by written informed consent that was obtained from each participating children’s parents or legal guardians. | | | | | | | | | | | | | | | | | | | | | |  |  |  |
| Participants | 4a | | | Eligibility criteria for participants | | | | | | | | When applicable, eligibility criteria for centers and those performing the interventions  Eligibility criteria for clusters | | | | | | | | | |  |  |  |
|  | 4b | | | Settings and locations where the data were collected | | | | | | | |  |  |  |  |  |  |  |  |  |  |  |  |  |
| Eligibility criteria for schools and participants  The following eligibility criteria were set for the clusters (schools): i) being a public primary school in the district of Limassol, Cyprus, and ii) being located within the urban area of Limassol. Following the bioethics approval of the study, 12 primary schools’ headmasters were randomly selected and contacted by the research team’s coordinator. Six schools accepted to implement the study. Eligible participants were healthy children attending any of the six selected primary schools with ages of 10 to 12 years old, residing in Cyprus, at least for the last five years, and systematically consuming conventional food (>80% of the week’s meals). Exclusion criteria included children with chronic conditions (e.g. diabetes, asthma) or allergies in food (e.g. gluten, lactose tolerance).  Recruitment process, setting for data collection  Informational flyers were offered to parents and information meetings were arranged to present study-relevant details and to provide study materials for the parents’ review (menus, details of study, forms of interest, consent forms for the parents/guardians of the participating child, and forms for breakfast and afternoon snack options). Parents that could not attend the meeting received the relevant material at home through the schools. Similar meetings, although using different study materials, were arranged with children in each of the six selected schools to inform them about the study, to explain to them the definition of organic food products (raw and processed), and to have them taste organic fruits. The importance of compliance to the study protocol was highlighted to both parents and children and the ways to report it in a diary, including examples of proper use. The research team checked if all eligibility criteria were satisfied, using the completed interest forms, and parents were accordingly informed via telephone. Following, informed consents were signed by parents of the children that were willing to participate. For eligible participants, a baseline questionnaire was administered to parents via telephone. Bags containing six coded urine vials, sampling dates, instructions for urine collection, and the food diary were given to children or to their parents during school hours. The organic restaurant was selected based on a public tender procurement procedure with some of the requirements being the use of 100% certified organic raw materials and food products, frequently renewed certificate for preparation of organic meals, and national accreditation for delivery of meals to schools, complying to ISO 22000 and HACCP protocols. | | | | | | | | | | | | | | | | | | | | | |  |  |  |
| Interventions† | 5 | | | The interventions for each group with sufficient details to allow replication, including how and when they were actually administered | | | | | | | Precise details of both the experimental treatment and comparator  Whether interventions pertain to the cluster level, the individual participant level, or both | | | | | | | | | | |  |  |  |
| Each school that participated in the study was *a priori* randomized to one of the two groups depending on the sequence of the treatments; organic-conventional (Group 1) and conventional-organic (Group 2). The order in which participants undertook the organic and conventional periods of the study depended on their school, hence the intervention randomization pertained to the cluster/school level. Schools in Group 1 began first with the organic period and continued with the conventional period whereas schools in Group 2 began first with the conventional period and then switched to the organic period. During the conventional period, participants were asked to maintain their usual dietary habits and choices (>80% conventional diet) for a total of maximum 40 days. The organic period was organized in two 20-day cycles with differences in the menu to allow for greater variety in the meals, so that the participants’ compliance to the organic treatment would be enhanced. During the organic period, participants were asked to strictly follow the two 20-day organic dietary menus provided to them for a total of 40 days. The organic dietary menus were prepared by a certified dietitian based on EFSA energy requirements for children aged 10-12 years,^3^ and included five meals per day; breakfast, morning snack, lunch, afternoon snack, and dinner. The meals of the organic period were delivered to schools Monday to Saturday, except for Sunday. Upon arrival of daily meals to each school, the teachers and headmaster were responsible for handing the packages to each participating child, using a project participant list around lunch time. On Saturday, the food package included both the Saturday and Sunday meals; for the Sunday meals, the foods were raw so that the parents would cook them fresh on Sunday. The meals of lunch and dinner were the same for all participants. For the breakfast, morning and afternoon snacks, children could choose based on a list of available products before the beginning of the organic period. Participants crossed over to the alternate diet on the following day after the first period was completed. A washout period was not required; it was intrinsically included in the two periods, since the first urine sample of the second period was collected about 12 days after the beginning of the second period and the pesticides half-lives are short (half-life for pyrethroids is less than 12 h and those for neonics range from 5-33 hours), so no carryover effect was expected.^4,5^ | | | | | | | | | | | | | | | | | | | | | |  |  |  |
| Each participant provided maximum six first morning urine samples during the whole duration of the 2-period study; one baseline sample, two samples in the conventional period, and three samples in the organic period (Fig.1). Anthropometric measurements (weight, height, and waist circumference) were taken at the beginning of the study, at the end of the organic period, and at the end of the study (for Group 2, the end of study and end of organic period was the same time point) by trained researchers at the school premises.^6^ A baseline questionnaire was administered to parents at the beginning of the study through a telephone interview to collect information on demographic characteristics the child’s lifestyle/behaviour habits, and possible non-dietary pesticide exposure sources. A food frequency questionnaire was administered to the parents at the end of the conventional period through a telephone interview to collect information about the food habits of the children during the conventional period. A food diary was provided to the parents at the beginning of the study and parents completed it during the organic period, in order to collect information about the compliance of the children to the organic dietary menu. | | | | | | | | | | | | | | | | | | | | | |  |  |  |
|  | 5a | | |  | | | Description of the different components of the interventions and, when applicable, descriptions of the procedure for tailoring the interventions to individual participants | | | | | | | | | | | | | | |  |  |  |
| Reported in item 5 | | | | | | | | | | | | | | | | | | | | | |  |  |  |
|  | 5b | | |  | | | Details of whether and how the interventions were standardized. | | | | | | | | | | | | | | |  |  |  |
| The organic diet intervention was standardized as a single restaurant was the only certified provider of the organic meals for the participants. Moreover, we attempted to standardize the organic meals using two indicative 20-day dietary menus that were prepared by a registered dietitian based on the average energy requirements of children 10-12 years old. However, because children may have different needs based on their activity level and metabolism, parents were advised to complement their children’s diet with certified organic products, if it was deemed necessary, and that their children shouldn’t be forced to consume the total quantity of their organic meals, if they were satisfied with a smaller portion. In the case that any deviations from the organic menu (consumption of conventional food items during a party, school event, societal event, etc.) occurred, parents were a priori advised to simply note it in the food diary. In cases of absence from school (e.g. for health reasons, flue, etc.), the parents were responsible to pick up the meals from school, note in the diary the days of absence and self-report the medical reason of absence. If the child couldn’t eat the organic food because he/she was sick, then the parents would also note that in the diary if other foods were consumed (for example soup). In case that children/parents forgot to pick up the organic meals from their schools, the school would inform a member of the research team. The research team member had access to the contact details of the participants and would call them to arrange the pick-up of the organic food. | | | | | | | | | | | | | | | | | | | | | |  |  |  |
|  | 5c | | |  | | | Details of whether and how adherence of care providers to the protocol was assessed or enhanced | | | | | | | | | | | | | | |  |  |  |
| Since there was only one provider of organic meals to schools, the same raw organic materials/ingredients and products, preparation of meals, and delivery of organic packages were applied to all participants. The research team was in close communication with the organic meal provider and delivery team to ensure that the meals preparation and menu options for each day were strictly followed based on the 20-day organic menu cycle. The research team was conducting frequent checks in school during meal delivery hours and during organic meal hand out to participants to ensure adherence to the menu. The team had also weekly visits to each school to interact with student participants asking their opinion on the food choices and the menu quality. Children and parents opinions about the menu were routed back to the organic restaurant to enhance the adherence of the menu protocol to the participant needs. The participants’ parents were given access to a hotline that was referring them to the trained researcher for communicating any issues with their meals; the research team was in daily communication with the restaurant ensuring that a full cycle of quality control and participant opinions were feeding back into the standardised protocol of the organic period. | | | | | | | | | | | | | | | | | | | | | |  |  |  |
|  | 5d | | |  | |  | Details of whether and how adherence of participants to interventions was assessed or enhanced | | | | | | | | | | | | | | |  |  |  |
| The adherence of participants to the organic diet intervention was assessed with a food diary in which parents were instructed to record any exceptions to the organic diet. Moreover, the diary was used to note any sickness and any pesticide use at home. The frequent telephone communication with parents, the weekly visits at schools, and the daily communication with the restaurant were key factors that further enhanced the compliance to the organic diet treatment. In order to encourage adherence to the organic diet until the end of the period, a public event was organized during the mid-period of the study with activities for the participating children and free sampling of organic food products. | | | | | | | | | | | | | | | | | | | | | |  |  |  |
| Outcomes | 6a | | | Completely defined pre-specified primary and secondary outcome measures, including how and when they were assessed | | | Whether outcome measures pertain to the cluster level, the individual participant level, or both | | | | | | | | | | |  | |  |  |  |  |  |
| Per the trial protocol, the primary outcomes were the biomarkers of exposure to pesticides using two non-specific pesticides metabolites (3-phenoxybenzoic acid (3-PBA), 6-chloronicotinic acid (6-CN)) measured in first morning urine voids collected any two up to six time points (full participation of child all 80 days of the trial). The secondary outcomes were the biomarkers of oxidative stress/inflammation (8-iso-prostaglandin F2a (8-iso-PGF2a), malondialdehyde (MDA) and 8-hydroxy-2′-deoxyguanosine (8-OHdG)) measured in the same urine samples.  In post-hoc analysis, we also reported the effect of the intervention on age and sex-adjusted BMI standard deviation scores (SDS) using the WHO 2007 growth reference standard for children.^7^ BMI SDS were calculated based on the measurements of weight and height taken at the baseline and end of the organic period (two timepoints), adjusting for age and sex. We also reported the observational associations of 3-PBA with OSI biomarkers as post-hoc ancillary analyses. | | | | | | | | | | | | | | | | | | | | | |  |  |  |
|  | 6b | | | Any changes to trial outcomes after the trial commenced, with reasons | | | | |  | | | | | | | | | | | | |  |  |  |
| The only change that took place was the addition of the BMI SDS as a post-hoc outcome because of the BMI importance in often mediating associations between exposures to environmental stressors and metabolic outcomes. However, this was a post-hoc analysis since the current study design could not support the investigation of biological plausibility mechanisms by which the intervention could influence the magnitude and variability of BMI scores. Furthermore, we cannot exclude the possibility that the participants were consuming a different amount of calories or changed their habits during the organic phase as opposed to the conventional diet, as the current study was not designed to delivering strict isocaloric diets at the two phases (but participants were free to consume as much they wished). Additionally, the food frequency questionnaire that was used at the end of the conventional period aimed to capture the overall habits of the participants, and we have not recorded deviation from the organic diet in which extra or less organic food - compared to what was provided - was consumed during the organic period as all participants were advised to avoid disturbing usual eating habits. | | | | | | | | | | | | | | | | | | | | | |  |  |  |
| Sample size | 7 | | | How sample size was determined and, when applicable, explanation of any interim analyses and stopping rules | | | | | When applicable, details of whether and how the clustering by care providers or centers was addressed  Method of calculation, number of clusters(s) (and whether equal or unequal cluster sizes are assumed), cluster size, a coefficient of intracluster correlation (ICC or k), and an indication of its uncertainty | | | | | | | | | | | | |  |  |  |
| In order to estimate the sample size of this crossover study, the PS software for calculating sample size was used.^8^ We used the paired t test study design for calculating the power of this test, assuming that each subject has a pair of values (one for conventional and one for organic diet). The variable used for the estimation of the sample size was a biomarker of inflammation (C-reactive protein), because the biomarkers of inflammation/oxidative stress were one the primary outcomes for the trial and we information on pesticide levels were unavailable at that time. The lack of pertinent human studies with similar setting and characteristics to ours that involved exactly the same biomarkers of effect (8-OHdG and isoprostanes) led us to use the available human study of CRP.^9^ | | | | | | | | | | | | | | | | | | | | | |  |  |  |
| The input parameters were: significance level, within patient standard deviation or standard deviation of the difference between the two values for the same patient, power and minimal detectable difference in means. Based on the literature, the within subject standard deviation of CRP was assumed to be 0.5^9^ and we hypothesized a minimal detectable difference in biomarkers of effect (CRP) between the organic and conventional treatment to be 0.1, based on other intervention studies^10–12^. Assuming a power of 80% and a two-sided 5% significance level, the sample size for this two-treatment crossover study was estimated to be 200 children. The clustering was not addressed, but the within cluster within period and the within cluster between period intra cluster correlation estimates were assumed equal. | | | | | | | | | | | | | | | | | | | | | |  |  |  |
| Randomization– sequence generation† | 8 | | | Method used to generate the random allocation sequence, including details of any restriction (e.g., blocking, stratification) | | | | | | | | | | | Details of stratification or matching if used | | |  | |  |  |  |  |  |
| Schools were randomly assigned following simple randomization procedures of no restriction or matching (computerized random numbers) to 1 of 2 groups (organic-conventional or conventional-organic). | | | | | | | | | | | | | | | | | | | | | |  |  |  |
| Allocation concealment | 9 | | | Mechanism used to implement the random allocation sequence (such as sequentially numbered containers), describing any steps taken to conceal the sequence until interventions were assigned | | | | | | | | | |  | | Specification that allocation was based on clusters rather than individuals and whether allocation concealment (if any) was at the cluster level, the individual participant level, or both | | | | | |  |  |  |
| Allocation was done at the school level rather than the individual level. The interventions were assigned after receiving the confirmation from the headmaster, before the meeting with the parents and the children. The allocation sequence could not be concealed from the participants since they knew when they were consuming their typical diet and when the organic meals. Also, the allocation sequence could not be concealed from the researchers enrolling the participants since they were informing the participants of the relevant information, such as the first day of the organic meals pick-up and the last day of the organic period. The allocation sequence, however, was concealed from the researchers performing the biomarker analysis since the urine vials were coded. | | | | | | | | | | | | | | | | | | | | | |  |  |  |
| Implementation | 10 | | | Who generated the allocation sequence, who enrolled participants, and who assigned participants to interventions | | | | | | | Who generated the random allocation sequence, who enrolled clusters, and who assigned clusters to interventions | | | | | | | | | | | |  | |
| Simple randomization was performed by a computer generated random list that was prepared by an investigator with no clinical involvement in the trial. A member of the research team enrolled participants after knowing the allocation sequence for each school since parents and children should be informed of the order the intervention was going to take place. | | | | | | | | | | | | | | | | | | | | | |  |  |  |
| Blinding | 11a | | | If done, who was blinded after assignment to interventions (for example, participants, care providers, those assessing outcomes) and how | | | | | | | | If done, who was blinded after assignment to interventions (e.g.,  participants, care providers, those administering co-interventions, those assessing outcomes) and how | | | | | |  | |  |  |  |  |  |
| The blinding of the participants to group assignment was not possible, since participants knew which diet they were following. The blinding of the researchers to the subjects’ identity was achieved by the coding of all study materials (urine containers, questionnaires, and diaries). The study personnel who obtained the outcome measurements were also not informed of the group assignment. The personnel who delivered the intervention did not take any outcome measurements. All outcome assessors and data analysts were kept masked to the allocation. | | | | | | | | | | | | | | | | | | | | | |  |  |  |
|  | 11b | | | If relevant, description of the similarity of interventions | | | |  | | | | | | | | |  | | | | |  |  |  |
| Not relevant. | | | | | | | | | | | | | | | | | | | | | |  |  |  |
|  | 11c | | |  | | | | | | | If blinding was not possible, description of any attempts to limit bias | | | | | | |  | |  |  |  |  |  |
| Not relevant. | | | | | | | | | | | | | | | | | | | | | |  |  |  |
| Statistical methods† | 12 | | | **Statistical methods used to compare groups for primary outcome(s);** methods for additional analyses, such as subgroup analyses and adjusted analyses | | | | When applicable, details of whether and how the clustering by care providers or centers was addressed  How clustering was taken into account | | | | | | | | | |  | |  |  |  |  |  |
| During the design phase of the study, power calculations were conducted to estimate the optimal sample size of a study assuming 80% power with a two-sided 5% significance level to detect a minimal detectable difference of 0.1 units in OSI biomarkers between the organic and conventional treatment, while the within subject standard deviation of the biomarker of effect was assumed to be 0.5. The clustering was not addressed, but the within cluster within period and the within cluster between period intra cluster correlation estimates were assumed equal. | | | | | | | | | | | | | | | | | | | | | |  |  |  |
| Descriptive statistics were used to summarize the demographic and other characteristics of participating children. Categorical variables were described with frequencies and percentages, normally-distributed continuous variables as mean±SD and non-normal continuous variables as median and interquartile range (25th-75th percentiles). Box plots were used to visually inspect the biomarker data. For data below LOD, imputation was performed based on existing suggestions for handling non-detect data^13^. For 6-CN and 3-PBA, the percentage of data below LOD was 72% and 23% accordingly, so the imputation was performed using the regression on order statistics (ROS). For MDA and creatinine, the percentage of data below LOD was very low (≈ 1%), so the LOD/2 imputation was used. The baseline characteristics were compared between the study groups with t-test for the normal continuous variables or Wilcoxon test, and chi-square for the normal, non-normal, and for the categorical variables, respectively.  The percent change between the baseline (last sample of conventional treatment before the start of the organic treatment) and after 40 days of organic diet (last sample of organic treatment) was determined with one sample t-test for all biomarkers (log-transformed) and the change between the conventional and organic treatment (medians) was determined using Wilcoxon test for all biomarkers (non-normal variables). | | | | | | | | | | | | | | | | | | | | | |  |  |  |
| **Primary analysis of outcomes**  Linear mixed-effect regression models were used for continuous outcomes and logistic models for binary variables (i.e. those with >70%values below LOD), to account for the duration and the effect of treatment. To account for the clustering of participants within schools and the repeated measures within person, the models will include student-level and school-level random intercepts with unsupervised covariance matrix.  Fixed effects were: the treatment condition (organic or conventional) and time (days of treatment, where time=0 was the start of the treatment). An interaction term for the treatment condition and time were considered and dropped when not meeting the threshold of p-value=0.05. The models were adjusted for the baseline value of the outcome in order to account for the background biomarker levels of the participant, The baseline sample was a conventional sample for both groups but it was not consecutive to the conventional phase due to the study design and, thus, it is indicative of each participants exposure prior to participation in the study  In all regression models, the continuous variables, other than time, were centred at the population means.  **Post-hoc analysis**  Post-hoc ancillary analyses to help towards the interpretation of the results included:  -Regression models that describe the association of primary outcomes with secondary outcomes adjusting for the baseline levels of each outcome, time, age and sex.  -Regression models that describe the impact of organic diet on participants’ BMI SDS with or without conditioning on the outcomes as candidate mediators. These models included only participants with available anthropometric measurements before and after the organic treatment, hence only two timepoints for the BMI SDS and the biomarkers were used.  **Sensitivity analysis**  Two sets of sensitivity analyses were undertaken based on the primary analysis of the outcomes:  -Regression models that were not adjusted for the baseline value of the outcome. Hence, the baseline sample excluded from the analysis.  -Regression models excluding the two participants who followed the opposite order of treatment compared to the group their school was allocated.  Estimation of the geometric mean ratios and odds ratios  Geometric mean ratios (GMR) of the outcomes and 95% confidence intervals (95% CIs) were estimated exponentiating the regression parameters from the linear mixed-effect regression models. Odds ratios (OR) and 95% CIs were estimated exponentiating the regression parameters from the logistic regression models. | | | | | | | | | | | | | | | | | | | | | |  |  |  |
| **Results** | | | | | | | | | | | | | | | | | | | | | |  |  |  |
| Participant flow (a diagram is strongly recommended) | | 13a | | | For each group, the numbers of participants who were randomly assigned, received intended treatment, and were analyzed for the primary outcome | | | | | For each group, the numbers of clusters that were randomly assigned, received intended treatment, and were analysed for the primary outcome | | | | | | | | | | | |  |  |  |
|  | | 13b | | | For each group, losses and exclusions after randomization, together with reasons | | | | |  | | | | | | | | | | | |  |  |  |
|  | | 13c | | |  | | | | | For each group, the delay between randomization and the initiation of the intervention | | | | | | | | | | | |  |  |  |
|  | | | new | |  | | | | | Details of the experimental treatment and comparator as they were implemented | | | | | | | | | | | |  |  |  |
| The participant flow details and diagram are included in the “Results” section of the manuscript. | | | | | | | | | | | | | | | | | | | | | |  |  |  |
| Recruitment | | | 14a | | Dates defining the periods of recruitment and follow-up | | | | |  | | | | | | | | | | | |  |  |  |
|  | | | 14b | | Why the trial ended or was stopped | | | | |  | | | | | | | | | | | |  |  |  |
| The study tool place during January-April 2017. The study ended according to the study design.  The dates are described in the “Methods” section of the manuscript. | | | | | | | | | | | | | | | | | | | | | |  |  |  |
| Baseline data | | | 15 | | A table showing baseline demographic and clinical characteristics for each group | | | | | When applicable, a description of care providers (case volume, qualification, expertise, etc.) and centers (volume) in each group | | | | | | | | | | | |  |  |  |
|  | | |  | |  | | | | | Baseline characteristics for the individual and cluster levels as applicable for each group | | | | | | | | | | | |  |  |  |
| The table with the baseline data is included in the “Results” section of the manuscript | | | | | | | | | | | | | | | | | | | | | |  |  |  |
| Numbers analysed | | | 16 | | For each group, number of participants (denominator) included in each analysis and whether the analysis was by original assigned groups | | | | | For each group, number of clusters included in each analysis | | | | | | | | | | | |  |  |  |
| The numbers analysed are described in the “Results” section of the manuscript. | | | | | | | | | | | | | | | | | | | | | |  |  |  |
| Outcomes and estimation | | | 17a | | For each primary and secondary outcome, results for each group, and the estimated effect size and its precision (such as 95% confidence interval) | | | | | Results at the individual or cluster level as applicable and a coefficient of intracluster correlation (ICC or k) for each primary outcome | | | | | | | | | | | |  |  |  |
|  | | | 17b | | For binary outcomes, presentation of both absolute and relative effect sizes is recommended | | | | |  | | | | | | | | | | | |  |  |  |
| Ancillary analyses | | | 18 | | Results of any other analyses performed, including subgroup analyses and adjusted analyses, distinguishing pre-specified from exploratory | | | | |  | | | | | | | | | | | |  |  |  |
| The primary and secondary outcomes results are described in the “Results” section of the manuscript. | | | | | | | | | | | | | | | | | | | | | |  |  |  |
| Harms | | | 19 | | All important harms or unintended effects in each group | | | | |  | | | | | | | | | | | |  |  |  |
| Not relevant | | | | | | | | | | | | | | | | | | | | | |  |  |  |
| **Discussion** | | |  | |  | | | | |  | | | | | | | | | | | |  |  |  |
| Limitations | | | 20 | | Trial limitations, addressing sources of potential bias, imprecision, and, if relevant, multiplicity of analyses | | | | | In addition, take into account the choice of the comparator, lack of or partial blinding, and unequal expertise of care providers or centers in each group | | | | | | | | | | | |  |  |  |
| Generalizability | | | 21 | | Generalizability (external validity, applicability) of the trial findings | | | | | Generalizability (external validity) of the trial findings according to the intervention, comparators, patients, and care providers and centers involved in the trial  Generalisability to clusters and/or individual participants (as relevant) | | | | | | | | | | | |  |  |  |
| Interpretation | | | 22 | | Interpretation consistent with results, balancing benefits and harms, and considering other relevant evidence | | | | |  | | | | | | | | | | | |  |  |  |
| The above are described in the “Discussion” section of the manuscript. | | | | | | | | | | | | | | | | | | | | | |  |  |  |
| **Other information** | | |  | |  | | | | |  | | | | | | | | | | | |  |  |  |
| Registration | | | 23 | | Registration number and name of trial registry | | | | |  | | | | | | | | | | | |  |  |  |
| The registration number is reported in the abstract of the manuscript. | | | | | | | | | | | | | | | | | | | | | |  |  |  |
| Protocol | | | 24 | | Where the full trial protocol can be accessed, if available | | | | |  | | | | | | | | | | | |  |  |  |
| **Trial registration number:** NCT02998203  The full trial protocol is included here and in the clinicaltrials.gov website | | | | | | | | | | | | | | | | | | | | | |  |  |  |
| Funding | | | 25 | | Sources of funding and other support (such as supply of drugs), role of funders | | | | |  | | | | | | | | | | | |  |  |  |
| The trial was designed by the Cyprus International Institute for Environmental and Public Health and neither the funder (EU LIFE+ programme) nor the sponsoring organic products companies were involved with the design or conduct of the trial and they were not involved in data collection or analysis. | | | | | | | | | | | | | | | | | | | | | |  |  |  |

## Supplementary References

1. Boutron I, Altman DG, Moher D, Schulz KF, Ravaud P, for the CONSORT NPT Group. CONSORT Statement for Randomized Trials of Nonpharmacologic Treatments: A 2017 Update and a CONSORT Extension for Nonpharmacologic Trial Abstracts. Ann Intern Med 2017;167(1):40.

2. Campbell MK, Piaggio G, Elbourne DR, Altman DG, for the CONSORT Group. Consort 2010 statement: extension to cluster randomised trials. BMJ 2012;345(sep04 1):e5661–e5661.

3. European Food Safety Authority (EFSA) Panel on Dietetic Products, Nutrition and Allergies (NDA), Scientific Opinion on Dietary Reference Values for energy. EFSA Journal 2013; 11(1): 3005. doi:10.2903/j.efsa.2013.3005.

4. ATSDR - Toxicological Profile: Pyrethrins and Pyrethroids [Internet]. [cited 2018 May 23];Available from: https://www.atsdr.cdc.gov/toxprofiles/tp.asp?id=787&tid=153

5. Harada, KH, Tanaka K, Sakamoto H et al. Biological Monitoring of Human Exposure to Neonicotinoids Using Urine Samples, and Neonicotinoid Excretion Kinetics. PLoS ONE 2016; 11(1). doi: 10.1371/journal.pone.0146335.

6. NHANES. Anthropometry Procedures Manual. 2007. [cited 2018 May 17]; Available from: https://wwwn.cdc.gov/nchs/data/nhanes/2007-2008/manuals/manual_an.pdf\

7. De Onis M. Development of a WHO growth reference for school-aged children and adolescents. Bull World Health Organ 2007; 85(09): 660–7.

8. Dupont WD and Plummer WD. PS: Power and Sample Size Calculation version 3.1.2, 2014. http://biostat.mc.vanderbilt.edu/wiki/Main/PowerSampleSize

9. Koenig W. Update on C-reactive protein as a risk marker in cardiovascular disease. Kidney Int 2003;63:S58–61.

10. Dourado GKZS, Cesar TB. Investigation of cytokines, oxidative stress, metabolic, and inflammatory biomarkers after orange juice consumption by normal and overweight subjects. Food Nutr Res 2015;59(1):28147.

11. Huang T, Larsen KT, Møller NC, Ried-Larsen M, Frandsen U, Andersen LB. Effects of a multi-component camp-based intervention on inflammatory markers and adipokines in children: A randomized controlled trial. Prev Med 2015;81:367–72.

12. Izadpanah A, Barnard RJ, Almeda AJE, et al. A short-term diet and exercise intervention ameliorates inflammation and markers of metabolic health in overweight/obese children. Am J Physiol-Endocrinol Metab 2012;303(4):E542–50.

13. Helsel DR. More than obvious: better methods for interpreting nondetect data. Environ Sci Technol 2005;39:419A-423A.
