# Supplementary material for: A cluster-randomized crossover trial of organic diet impact on biomarkers of exposure to pesticides and biomarkers of oxidative stress/inflammation in primary school children
Source: PLoS One. 2019 Sep 4;14(9):e0219420. doi: 10.1371/journal.pone.0219420 (PMC6726134; doi:10.1371/journal.pone.0219420)
Supplement: S2 Text — (DOCX) [file pone.0219420.s004.docx]

Additional text

A cluster-randomized crossover trial of organic diet for primary school children

*Konstantinos C. Makris^1*^, PhD, Corina Konstantinou^1^, MS, Xanthi D. Andrianou^1^, MS, Pantelis Charisiadis^1^, PhD, Alexis Kyriacou^2^, RD, Matthew O. Gribble^3,4^^, PhD DABT, and Costas A. Christophi^1^^, PhD*

^1^ Cyprus International Institute for Environmental and Public Health, Cyprus University of Technology, Limassol, Cyprus

^2^ Faculty of Health Sciences and Sport, University of Stirling, Stirling, UK

^3^ Department of Environmental Health, Emory University, Atlanta, GA, USA

^4^ Department of Epidemiology, Emory University, Atlanta, GA, USA

^ Both authors contributed equally to this work.

* Corresponding author: Konstantinos Christos Makris, Associate Professor of Environmental Health, Cyprus International Institute for Environmental and Public Health, School of Health Sciences, Cyprus University of Technology, Limassol, Cyprus.

Phone: 357-25002398, FAX: 357-25002676

E-mail: [konstantinos.makris@cut.ac.cy](mailto:konstantinos.makris@cut.ac.cy) (KCM)

##

## Food diary digitization notes

**Important**

Digitization of the diaries will be only taking place on the premises of the Water and Health Laboratory by taking into consideration the comments written bellow.

Hard or soft copies (i.e. questionnaires, consent forms, name lists, files etc) of material that might contain personal information of the participants should under no circumstances be communicated to anyone without the approval of the Principal Investigator. Additionally, hard copies should never be removed from the Laboratory.

All the process of the digitization will take place on the laboratory computers and university email accounts should be used for all email communication with regards to the digitization process.

**Introduction**

The food diary is used by children during the organic phase of the study. The parents note with tick (✓) for meals that have been consumed by their children according to the organic menu without any additions of conventional foods and write down any changes they made in their diet. It is important that they note down only the conventional foods they have consumed in the appropriate part of the diary. If they have made changes with regards to the order they ate the meals of the organic menu, they don’t need to note them down.

**Data entry**

Microsoft Excel was used for the digitization of the data.

**Notes to take into account when entering data**

- The final results of the diary digitization are percentages of non-compliance for each participant.
- This percentage is calculated by taking in account that each meal of the day has a specific weight.
- The breakfast, lunch and dinner weigh 1 point, the morning and afternoon snacks weigh 0.5 point and small changes in breakfast, lunch and dinner weigh 0.5 point.
- Each week has a total of 28 points.
- The researcher notes the points for non-compliance of the menu, i.e. the points for meals that have been replaced with conventional food or there has been an addition of conventional foods.
- These points are noted for each day of the week and then a sum is calculated.
- The sum is then divided by 28 (total points for a week) and multiplied by 100 (to calculate the percentage of non-compliance for a week).
- The sum of all points is then divided by the number of days, for which there are data multiplied by 4 (e.g 38 days * 4) and multiplied by 100 (to calculate the percentage of non-compliance for the total period).
- The days before sampling are highlighted (need to check individually for each child its sampling dates) and again the percentage of non-compliance is estimated by dividing by 4 (total points for a day) & multiplying by 100.
- For days that we don’t have data because the child left the study, we write NA (meaning “Not applicable”)
- For days/diaries that we don’t have data because the child lost the diary or didn’t note, we write ND (meaning “No data”)
- For weeks that are not complete, either because the child left the study or because the study is completed before the end of the week, the percentage of non-compliance needs to be calculated according to the days of the week (e.g. 2 days of week 6 because study is completed -> % of non-compliance = sum/8 * 100)
- Regarding the pesticides use & illness occurrence, the following are applied:
  - If no pesticides are used & no illness was presented during the whole period, the letter N is written
  - If pesticides are used or/and illness is presented in one or more weeks, the week number is written and any other relevant info.
  - If there isn’t a tick on either Yes or No for pesticides use and/or presence of illness, the letters ND are written.
- After the end of the digitization, a number of questionnaires (40%) are checked by another researcher in order to see if the digitization was done correctly.
  - Regarding the percentage of non-compliance for the total period, if the value calculated by the second researcher is different by <3%, then the result is considered correct.
  - Regarding the percentage of non-compliance for the total period, if the value calculated by the second researcher is different by ≥3%, then the result is considered wrong and re-calculation is needed.
  - Regarding the percentage of non-compliance for the day before sampling, if the value calculated by the second researcher is different by <3%, then the result is considered correct.
  - Regarding the percentage of non-compliance for the day before sampling, if the value calculated by the second researcher is different by ≥3%, then the result is considered wrong and re-calculation is needed.
- Abbreviations:
  - DbS1: % of non-compliance on day before first sample in organic phase
  - DbS2: % of non-compliance on day before second sample in organic phase
  - DbS3: % of non-compliance on day before third sample in organic phase
  - Total_period: % of non-compliance for the total organic phase (days with ND are not included in the calculation)

Table 1 Weight estimations for meals

| **Points** |  |
| --- | --- |
| 1 | Breakfast, lunch, dinner |
| 0.5 | Morning and afternoon snacks |
| 0.5 | Small changes in breakfast, lunch, dinner |
| 28 | Total for week |

Table 2. Example of table for calculating the non-compliance of a participant. First, the non-compliance of each day of the week is noted, then the sum of the week is calculated and then the percentage of non-compliance for a week.

| **[Participant code]** | | | | | | |
| --- | --- | --- | --- | --- | --- | --- |
|  | **Week 1** | **Week 2** | **Week 3** | **Week 4** | **Week 5** | **Week 6** |
| **Day 1** | 2 | 0 | 0 | 0 | 1 | 0 |
| **Day 2** | 1.5 | 0 | 1 | 1 | 0 | 1 |
| **Day 3** | 1 | 1 | 0 | 1.5 | 0 | 2 |
| **Day 4** | 1 | 0 | 0 | 1 | 0 | 0.5 |
| **Day 5** | 0 | 1 | 0.5 | 0 | 1 | 1.5 |
| **Day 6** | 0.5 | 1 | 0 | 0.5 | 2 | 0 |
| **Day 7** | 0 | 1 | 1.5 | 1 | 1 | 0 |
| **Sum** | 6 | 4 | 3 | 5 | 5 | 5 |
| **% non compliance** | 21 | 14 | 11 | 18 | 18 | 18 |

Table 3 Example of table for summarizing the results of non-compliance for all participants

| **Percentage of non-compliance** | | | | | |
| --- | --- | --- | --- | --- | --- |
|  | **[Participant code]** | **[Participant code]** | **[Participant code]** | **[Participant code]** | **[Participant code]** |
| **Week 1** | 21 | 23 | 20 | 21 | 13 |
| **Week 2** | 14 | 40 | 20 | 10 | 11 |
| **Week 3** | 11 | 10 | 11 | 5 | 7 |
| **Week 4** | 18 | 25 | 20 | 3 | 9 |
| **Week 5** | 12 | 13 | 11 | 10 | 18 |
| **Week 6** | 8 | 10 | 5 | 10 | 15 |

Table 4 Example of table for digitizing the use of pesticides and presence of illness

| **CODE** | **ILLNESS** | **PESTICIDES** |
| --- | --- | --- |
|  | N | N |
|  | Week 3 (15/3) | N |
|  | N | N |
|  | Week 5 (no drugs) | N |
|  | Week 6 (1/4) | N |
|  | N | N |
|  | N | N |
|  | N | N |
|  | Week 1 & 2 (cough) | N |
|  | N | N |
