# Supplementary material for: A cluster-randomized crossover trial of organic diet impact on biomarkers of exposure to pesticides and biomarkers of oxidative stress/inflammation in primary school children
Source: PLoS One. 2019 Sep 4;14(9):e0219420. doi: 10.1371/journal.pone.0219420 (PMC6726134; doi:10.1371/journal.pone.0219420)
Supplement: S3 Text — (PDF) [file pone.0219420.s005.pdf]

CYPRUS INTERNATIONAL INSTITUTE
FOR ENVIRONMENTAL & PUBLIC HEALTH

S3 Questionnaires

ID Number

FOOD HABITS QUESTIONNAIRE

CHILD'S FIRST AND LAST NAME: ……………………………………………………….

DATE: ……………………………………………………………….

In the last month, how many times have you consumed the following foods: e.g., if you consumed one glass of milk once per day, then you would write 1 in the column times/day and 7 in the column times/week. If it is once per month, then you would write 1 in the column times/month. If you don't remember or you don't know, then simply mark √ in the relevant box (see the example below).

Food	Consumption	Frequency				
		Never	Times/Day	Times/ Week	Times/ Month	I don't remember/ I don't know
Milk (glass of water, 250ml)	1 glass/day		1	7		
	2 glasses/day				2	
Yogurt, Full (Packaging 200g)	3 servings/ week			3		
Meat (portion=90gr)	2 μερίδες/1 ημέρα/ εβδομάδα		2	1		
	2 μερίδες/2 ημέρες/ εβδομάδα		2	2		
	Ποτέ	√				
Bread (slice=30gr)	2 slices/ημέρα		2	7		
Cheese (portion=30gr)	2 portions/week			2		
Boiled Vegetables , e.g. cauliflower etc. (½ cup)	1 cup/day		2 (1×2)	7		
	2 cups/ week			4 (2×2)		
Legumes (½ cup)	3 cups/week				6 (3×2)	
Fruits (number)	3 fruits/day		3	7		

QUESTIONNAIRE

FOOD	FREQUENCY				
	Never	Times/ Day	Times/ Week	Times/ Month	I don't remember/ I don't know
1. Milk (portion=size of glass of water, 250ml)					
**a.** Fresh whole					
**b.** Fresh low-fat					
**c.** Fresh fat-free					
**d.** Condensed whole					
**e.** Condensed low-fat					
**f.** Condensed fat-free					
**g.** Sweetened					
**h.** Chocolate					
2. Yogurt (plain or with fruits) (small package of 200g)					
**a.** Full					
**b.** Low-fat					
**c.** Fat-free					
3. Cheese (portions are about the size of a matchbox, portion=30gr)					
**a.** Parmesan, Graviera, Kasseri, Cheddar					
**b.** Feta, Halloumi, Fresh Anari, Edam					
**c.** Soft or Cream Cheeses and Spreads (e.g., Cottage, Philadelphia, La vache qui rit)					
Is the cheese that you usually eat low in fat? (Mark √ in the corresponding box)					
**a.** Never					
**b.** Sometimes					
**c.** Frequently					
**d.** I don't remember/I don't know					
4. Eggs (I don't know)					

**FOOD**	**FREQUENCY**				
	Never	Times/ Day	Times/ Week	Times/ Month	I don't remember/ I don't know
5. Morning cereals (medium-sized bowl)					
**a.** Cornflakes, Rice-Krispies, other cereals low in fiber					
**b.** All-Bran, Fruit 'n' Fiber, Muesli, other cereals high in fiber					
**c.** chocolate or sweetened, e.g.,. Coco-Pops, Frosties					
6. Bread, buns, loaf, pita, toast, crouttons (φέτα/slice=30γρ)					
**a.** White					
**b.** Village bread/Simit					
**c.** Black					
**d.** Wheat/Multi-Seeds					
7. Wheat (portion= ½ cup)					
**a.** Rice					
**b.** Bulgur					
**c.** Trahana					
8. Pasta					
**a.** Pasta, Orzo (porion= ½ cup)					
**b.** Stuffed Pasta (e.g., Ravioli, Tortellini) (portion= ½ cup)					
**c.** Pastitsio (one medium-sized piece) (number)					
9. Potatoes					
**a.** Fried (portion= 20-25 potatoes)					
**b.** Boiled (portion= 2 small potatoes – 90 gr)					
**c**. Baked (portion= 2 small potatoes – 90 gr)					
**d**. Puree (portion=½ cup)					
10. Legumes e.g.,. lentils, beans (portion=½ cup)					

FOOD	FREQUENCY				
	Never	Times/ Day	Times/ Week	Times/ Month	I don't remember/ I don't know
11. Meat, Meat and fish items					
**a.** Meat or minced meat: pork (e.g., steak, meatballs, souvlaki, souvla) (portion=90gr)					
**b.** Meat: lamb/goat (e.g., souvla, kleftiko) (portion=90gr)					
**c.** Meat or minced meat: beef (e.g., steak, hamburger) (portion=90gr)					
**d.** Chicken or rabbit (portion=90gr)					
**e.** Liver (portion=90gr)					
**f.** Sausages (portion=1 piece) (accept the times that are used specifically for sandwiches)					
**i.** Salami, bacon					
**ii.** Lountza, ham					
**g.** Sausages (portion=1 Frankfurt Sausage) (except those used in loukanokopites)					
**h.** Canned meat (e.g., ZWAN) (portion=120gr)					
**i.** Canned fish (e.g., tuna) (portion=120gr)					
**j.** fresh or frozen fish, seafood (calamari, octopus, etc.) (portion=90gr)					
12. Fruits and Vegetables					
**a.** Raw vegetables, salads (portion=1 cup)					
**b.** Cooked vegetables: greens, beans, cauliflower, etc. (portion=½ cup)					
**c.** Fresh fruits (portion=1 apple, 1 medium cluster of grapes, 15 cherries, 2 apricots, 1 medium-sized slice of watermelon)					
**d.** Fresh juice from fresh fruits or vegetables (glass of water, 250ml)					
13. Oils, Spreads, and Olives (which are used in cooking, served to compliment the main dish, and/or as bread spreads)					
**a.** Olive oil (tablespoon)					
**b.** Seed oil, Sunflower oil, etc. (tablespoon)					
**c.** Margarine (teaspoon)					
**d.** Low-fat margarine (e.g., Flora light/Vitalite) (teaspoon)					
**e.** Butter (teaspoon)					
**f**. Mayonnaise (teaspoon)					
**g.** Olives (number)					

FOOD	FREQUENCY				
	Never	Times/ Day	Times/ Week	Times/ Month	I don't remember/ I don't know
14. Sugar, honey, marmalade, etc.					
**a.** Sugar (teaspoon)					
**b.** Honey (teaspoon)					
**c.** Marmalade (etc.)					
**d.** Fruit Compote (portion=½ cup)					
15. Chocolates, biscuits, ice-cream, sweets, cake					
**a.** Merenda e.g., Nutella (teaspoon)					
**b.** Chocolates (e.g., one chocolate ION=45gr) List					
**c.** Wafers (e.g., one σοκοφρέτα=40gr)					
**d.** Cookies (portion=1 cookie)					
**e.** Ice-cream (portion=1 scoop)					
**f.** Creams (e.g., pudding, mousse) (1 medium-sized bowl)					
**g.** Cake, tarts, pie, sweets (1 medium-sized piece)					
16. Snacks and meals					
**a**. Cheese pies, souffle (number)					
**b.** Sausage pie, chicken pie (number)					
**c.** Tahinopita, olive pie (number)					
**d.** Croissant, donuts (number)					
**e.** Sandwhich (ham-cheese, lountza-halloumi) (number)					
**f.** Sandwhich (Cheese or halloumi) (number)					
**g**. Sandwhich (tuna) (number)					
**h.** Pizza (one large-sized slice) (number)					
**i**. Meals «Fast Food» (e.g., Mc Donald's, KFC) (number)					
**j.** Moussaka (one medium-sized piece) (number)					
**k.** Stuffed foods or dolmas (number)					

FOOD	FREQUENCY				
	Never	Times/ Day	Times/ Week	Times/ Month	I don't remember/ I don't know
17. Crisps, potato chips, popcorn (smalls bags)					
18. Nuts (small coffee cups)					
19. Drinks and Coffee					
**a.** Water (glasses)					
**b. Soda** (Cans, 330cc)					
**i.** Soda with sugar					
**ii.** Sugar-free soda (diet)					
**c. Juice** (1 box/1 glass 250ml)					
**i.** Concentrated juices (e.g., Lanitis, KEAN etc.)					
**ii.** Fruit juices (e.g., Keanita)					
**d. Other drinks** (1 box/bottle)					
**i.** Isotonic (e.g.,. Lucozade, Gatorade)					
**ii.** Energy drinks (e.g., Redbull, Shark)					
***20.* Supplements**					
**a.** Vitamins (specify ....)					
**b.** Other (e.g., protein drinks) (specify ……………………….............)					
***21.* Other foods**					
**a.**					
**b.**					

Thank you.

Κωδικός Αριθμός

ΕΡΩΤΗΜΑΤΟΛΟΓΙΟ ΣΥΧΝΟΤΗΤΑΣ ΔΙΑΤΡΟΦΗΣ

ΟΝΟΜΑΤΕΠΩΝΥΜΟ ΠΑΙΔΙΟΥ: …………………………………………………………

ΗΜΕΡΟΜΗΝΙΑ: ……………………………………………………………………

Τον τελευταίο μήνα, πόσες φορές κατανάλωσες τις παρακάτω τροφές: π.χ. αν κατανάλωσες 1 ποτήρι γάλα 1 φορά την ημέρα τότε θα γράψεις 1 στη στήλη <u>φορές/ημέρα</u> και 7 στη στήλη <u>φορές/εβδομάδα</u>, αν είναι 1 φορά την εβδομάδα τότε θα γράψεις 1 στη στήλη <u>φορές/εβδομάδα,</u> αν είναι 1 φορά το μήνα τότε θα γράψεις 1 κάτω από τη στήλη <u>φορές/μήνα</u> κι αν είναι ποτέ ή δεν θυμάσαι/δεν γνωρίζεις τότε θα σημειώσεις √ στο αντίστοιχο κουτάκι (βλέπε πίνακα παρακάτω).

**Τρόφιμα**	**Κατανάλωση**	**Συχνότητα**				
		Ποτέ	Φορές/ ημέρα	Φορές/ εβδομάδα	Φορές/ μήνα	Δεν θυμάμαι/ Δεν γνωρίζω
Γάλα (ποτήρι του νερού, 250ml)	1 ποτήρι/ημέρα		1	7		
	2 ποτήρια/μήνα				2	
Γιαούρτι πλήρες (συσκευασία 200γρ)	3 κεσεδάκια/ εβδομάδα			3		
Κρέας (μερίδα=90γρ)	2 μερίδες/1 ημέρα/ εβδομάδα		2	1		
	2 μερίδες/2 ημέρες/ εβδομάδα		2	2		
	Ποτέ	√				
Ψωμί (φέτα=30γρ)	2 φέτες/ημέρα		2	7		
Τυρί (μερίδα=30γρ)	2 μερίδες/εβδομάδα			2		
Λαχανικά βραστά , π.χ. κουνουπίδι κ.τ.λ. (½ φλιτζάνι)	1 φλιτζάνι/ημέρα		2 (1×2)	7		
	2 φλιτζάνια/ εβδομάδα			4 (2×2)		
Όσπρια (½ φλιτζάνι)	3 φλιτζάνια/μήνα				6 (3×2)	
Φρούτα (αριθμός)	3 φρούτα/ημέρα		3	7		

ΕΡΩΤΗΜΑΤΟΛΟΓΙΟ

ΤΡΟΦΙΜΑ	ΣΥΧΝΟΤΗΤΑ				
	Ποτέ	Φορές/ ημέρα	Φορές/ εβδομάδα	Φορές/ μήνα	Δεν θυμάμαι/ Δεν γνωρίζω
**1. *Γάλα*** (ένα ποτήρι νερού, 250ml)					
**α.** φρέσκο πλήρες					
**β.** φρέσκο ημιάπαχο					
**γ.** φρέσκο άπαχο					
**δ.** εβαπορέ πλήρες					
**ε.** εβαπορέ ημιάπαχο					
**στ.** εβαπορέ άπαχο					
ζ. ζαχαρούχο					
**η.** σοκολατούχο					
**2. *Γιαούρτι (σκέτο ή με φρούτα)*** (μικρή συσκευασία 200γρ)					
**α.** πλήρες					
**β.** ημιάπαχο					
**γ.** άπαχο					
**3. *Τυρί*** (μερίδες μεγέθους σπιρτόκουτου, μερίδα=30γρ)					
**α.** κεφαλοτύρι, γραβιέρα, κασέρι, Cheddar					
**β.** φέτα, χαλούμι, φρέσκα αναρή, Edam					
**γ.** μαλακό τυρί/κρεμώδες (π.χ. Cottage, Philadelphia, La vache qui rit)					
Το τυρί που τρως είναι χαμηλό σε λιπαρά; (σημείωσε √ στο αντίστοιχο κουτάκι)					
**α.** ποτέ					
**β.** μερικές φορές					
**γ.** συνήθως/πάντα					
**δ.** δεν θυμάμαι/δεν γνωρίζω					
**4. *Αυγά*** (αριθμός)					

ΤΡΟΦΙΜΑ	ΣΥΧΝΟΤΗΤΑ				
	Ποτέ	Φορές/ ημέρα	Φορές/ εβδομάδα	Φορές/ μήνα	Δεν θυμάμαι/ Δεν γνωρίζω
**5. *Δημητριακά προγεύματος (μέτριο μπολ)***					
**α.** Cornflakes, Rice-Krispies, άλλα χαμηλά σε φυτικές ίνες)					
**β.** All-Bran, Fruit ‘n’ Fiber, Muesli, άλλα ψηλά σε φυτικές ίνες					
**γ.** σοκολατούχα, ζαχαρούχα, π.χ. Coco-Pops, Frosties					
**6. *Ψωμί, φραντζόλα, πίτα, φρυγανιά, παξιμάδι*** (φέτα/slice=30γρ)					
**α.** άσπρο					
**β.** χωριάτικο ψωμί/κουλούρι					
**γ.** μαύρο					
**δ.** πολύσπορο					
**7. *Δημητριακά*** (μερίδα= ½ φλιτζάνι)					
**α.** ρύζι					
**β.** πλιγούρι (πουργούρι)					
**γ.** τραχανάς					
**8. *Ζυμαρικά***					
**α.** μακαρόνια, κριθαράκι (μερίδα= ½ φλιτζάνι)					
**β.** γεμιστά ζυμαρικά (π.χ. ραβιόλες, τορτελλίνια) (μερίδα= ½ φλιτζάνι)					
**γ.** μακαρόνια φούρνου (ένα μέτριο κομμάτι) (αριθμός)					
**9. *Πατάτες***					
**α.** τηγανιτές (μερίδα= 20-25 πατάτες)					
**β.** βραστές (μερίδα= 2 μικρές πατάτες - 90γρ)					
**γ.** φούρνου (μερίδα= 2 μικρές πατάτες - 90γρ)					
**δ.** πουρέ (μερίδα= ½ φλιτζάνι)					
**10. *Όσπρια π.χ. φακές, φασόλια*** (μερίδα=½ φλιτζάνι)					

ΤΡΟΦΙΜΑ	ΣΥΧΝΟΤΗΤΑ				
	Ποτέ	Φορές/ ημέρα	Φορές/ εβδομάδα	Φορές/ μήνα	Δεν θυμάμαι/ Δεν γνωρίζω
**11. *Κρέας, προϊόντα κρέατος και ψάρια***					
**α.** κρέας ή κιμάς: χοιρινό (π.χ. μπριζόλα, σιεφταλιές, σουβλάκι, χοιρινή σούβλα) (μερίδα=90γρ)					
**β.** κρέας: αρνί/κατσίκι (π.χ. αρνίσια σούβλα, αρνί στο φούρνο, κλέφτικο) (μερίδα=90γρ)					
**γ.** κρέας ή κιμάς: βοδινό (π.χ. στέικ, hamburger) (μερίδα=90γρ)					
**δ.** κοτόπουλο ή κουνέλι (μερίδα=90γρ)					
**ε.** συκώτι (μερίδα=90γρ)					
**στ.** αλλαντικά (μερίδα=1 φέτα) (εκτός αυτών που περιέχονται σε σάντουιτς)					
**i.** σαλάμι, μπέικον, μουρτατέλα					
**ii.** λούντζα, χαμ					
**ζ.** λουκάνικα (μερίδα=1 μέγεθος φραγκφούρτης) (εκτός αυτών που περιέχονται σε λουκανικόπιτες)					
**η.** κονσέρβες κρέατος (π.χ. ZWAN) (μερίδα=120γρ)					
**θ.** κονσέρβες ψαριού (π.χ. τόνος) (μερίδα=120γρ)					
**ι.** ψάρι φρέσκο ή κατεψυγμένο, θαλασσινά (χταπόδι, καλαμάρι κ.α.) (μερίδα=90γρ)					
**12. *Λαχανικά και φρούτα***					
**α.** λαχανικά ωμά, σαλάτες (μερίδα=1 φλιτζάνι)					
**β.** μαγειρευμένα λαχανικά: χόρτα, φασολάκι, κουνουπίδι κ.τ.λ. (μερίδα=½ φλιτζάνι)					
**γ.** φρέσκα φρούτα (μερίδα=1 μήλο, 1 μέτριο τσαμπί σταφύλι, 15 κεράσια, 2 χρυσόμηλα, 1 μέτρια φέτα καρπούζι)					
**δ.** χυμοί από φρέσκα φρούτα ή λαχανικά (ποτήρι νερού, 250ml)					
**13. *Λάδι, αλείμματα, και ελιές*** (που χρησιμοποιούνται στο μαγείρεμα, προστίθενται την διάρκεια του φαγητού στο τραπέζι, αλείφονται στο ψωμί)					
**α.** ελαιόλαδο (κουταλιές σούπας)					
**β.** σπορέλαιο, ηλιανθέλαιο κ.τ.λ. (κουταλιές σούπας)					
**γ.** μαργαρίνη (κουταλάκια γλυκού)					
**δ.** μαργαρίνη χαμηλή σε λιπαρά (π.χ. Flora light/Vitalite) (κουταλάκια γλυκού)					
**ε.** βούτυρο (κουταλάκια γλυκού)					
**στ.** μαγιονέζα (κουταλάκια γλυκού)					

**ΤΡΟΦΙΜΑ**	**ΣΥΧΝΟΤΗΤΑ**				
	Ποτέ	Φορές/ ημέρα	Φορές/ εβδομάδα	Φορές/ μήνα	Δεν θυμάμαι/ Δεν γνωρίζω
**ζ.** ελιές (αριθμός)					
**14. *Ζάχαρη, μέλι, μαρμελάδα κ.τ.λ.***					
**α.** ζάχαρη (κουταλάκια γλυκού)					
**β.** μέλι (κουταλάκια γλυκού)					
**γ.** μαρμελάδα & γλυκά κουταλιού (κουταλάκια γλυκού)					
**δ.** κομπόστα φρούτων (μερίδα=½ φλιτζάνι)					
**15. *Σοκολάτες, μπισκότα, παγωτά, γλυκά, κέικ***					
**α.** μερέντα π.χ. nutella (κουταλάκια γλυκού)					
**β.** σοκολάτες (π.χ. μια σοκολάτα ΙΟΝ=45γρ) προσδιόρισε					
**γ.** γκοφρέτες (π.χ. μια σοκοφρέτα=40γρ)					
**δ.** μπισκότα, κουλουράκια (μερίδα=1 μπισκότο ή κουλουράκι)					
**ε.** παγωτά (μερίδα=1 μπαλάκι/scoop)					
**στ.** κρέμες (π.χ. καραμελέ, μους) (1 μέτριο μπολ)					
**ζ.** κέικ, τούρτες, τάρτες, γλυκίσματα (1 μέτριο κομμάτι)					
**16. *Σνακς και γεύματα***					
**α.** τυρόπιτες, πουρέκια, σουφλέ (αριθμός)					
**β.** λουκανικόπιτες, κοτόπιτες (αριθμός)					
**γ.** ταχινόπιτες, ελιόπιτες (αριθμός)					
**β.** κρουασάν, ντόνατς (αριθμός)					
**δ.** σάντουιτς (ζαμπόν-τυρί, λούντζα-χαλούμι) (αριθμός)					
**ε.** σάντουιτς (τυρί ή χαλούμι) (αριθμός)					
**στ**. σάντουιτς (τόνος κ.α.) (αριθμός)					
**ζ.** πίτσα (ένα κομμάτι από μεγάλου μεγέθους πίτσα) (αριθμός)					
**η**. γεύματα «φαστ φουντ» (π.χ. Mc Donald's, KFC) (αριθμός)					
**θ.** μουσακάς (ένα μέτριο κομμάτι) (αριθμός)					

**ΤΡΟΦΙΜΑ**	**ΣΥΧΝΟΤΗΤΑ**				
	Ποτέ	Φορές/ ημέρα	Φορές/ εβδομάδα	Φορές/ μήνα	Δεν θυμάμαι/ Δεν γνωρίζω
**ι.** γεμιστά ή ντολμάδες (αριθμός)					
17. ***Γαριδάκια, πατατάκια, ποπ κορν*** (μικρά σακουλάκια)					
18. ***Ξηροί καρποί*** (φλιτζανάκια καφέ)					
19. ***Ποτά και καφέδες***					
**α.** νερό (ποτήρια)					
**β. αναψυκτικά** (τενεκεδάκια, 330cc)					
**i.** αναψυκτικά με ζάχαρη					
**ii.** αναψυκτικά χωρίς ζάχαρη (diet)					
**γ. χυμοί** (1 κουτί/1 ποτήρι 250ml)					
**i.** χυμοί συμπυκνωμένοι (π.χ. Λανίτη, KEAN κ.τ.λ.)					
**ii.** φρουτοποτά (π.χ. Κεανίτα)					
**δ. άλλα ποτά** (1 κουτί/μπουκάλι)					
**i.** ισοτονικά (π.χ. Lucozade, Gatorade)					
**ii.** energy drinks (π.χ. Redbull, Shark)					
20. **Συμπληρώματα**					
**α.** βιταμίνες (προσδιόρισε ....)					
**β.** άλλα (π.χ. πρωτεΐνες) (προσδιόρισε ……………………….............)					
21. **Άλλα φαγητά**					
**α.**					
**β.**					

Ευχαριστούμε.

ORGANIKO life+ | CYPRUS INTERNATIONAL INSTITUTE FOR ENVIRONMENTAL AND PUBLIC HEALTH

ΕΡΩΤΗΜΑΤΟΛΟΓΙΟ

Βιολογική Διατροφή και Υγεία Παιδιών

**Child's First and Last Name**			
**Parent's First and Last Name**			
**Primary Telephone**			
**Questionnaire ID**		**Date**	

<u>PART A</u>

Please answer the following questions:

1. Education level:

	**Mother**	**Father**
Primary Education	☐	☐
Secondary Education	☐	☐
University/College	☐	☐
Master's/Doctorate's	☐	☐
Other…………………………………	☐	☐

2. Mother's Occupation: ☐
3. Father's Occupation ☐
4. City/Village of Residence: ☐
5. ZIP Code: ☐
6. Length of Stay in Current Residence: ☐ months/years

7. How frequently have you used pesticides in your place of residence (including indoor and outdoor areas-garden, grass, lawn, trees) in the past 3 months? By pesticides, we refer to insect repellents, herbicides, insecticides, fungicides, or rat/mouse poisons.

	Outdoors	Indoors	
Insect repellents			Times /week/month
Insecticides			Times /week/month
Fungicides			Times /week/month
Herbicides			Times /week/month
Rat/Mouse Poison			Times /week/month

If you don't use pesticides, please continue at Part B.

8. When was the most recent use of pesticides in your place of residence? (days, weeks, months, etc.)?

	Outdoors	Indoors
Insect repellents		
Insecticides		
Fungicides		
Herbicides		
Rat/Mouse Poison		

9. Do you know which pesticides are usually used in your place of residence? Please list them:

Part B

Please answer the following questions with regards to your child participating in the study:

1. Child's Gender: Male ☐ Female ☐
2. Child's Birthday: ☐
3. Does your child have the tendency to put their fingers in their mouth (nail biting, finger/thumb sucking, etc.)?

   Yes ☐ No ☐
4. How much time does your child spend in gardens or parks on a weekly basis?

   Garden: ☐ hours/week

   Park: ☐ hours/week

5. How many hours per week does your child spend doing physical activities or sports on a weekly basis?

	**Hours/week**
Running	
Cycling	
Basketball	
Football/Soccer	
Volleyball	
Swimming	
Dance	
Other ………………………	

6. How much time does your child spend in passive activities on a weekly basis?

	**Hours/week**
Television	
Computer	
Tablet	
Mobile phone	
Other ………………………..	

Part C (Completed by a Research Member)

**Date**	**Starting Date (…./…./…….)**	**Final Date (…./…./…….)**
**Weight**		
**Height**		
**Waist circumference**		

ΕΡΩΤΗΜΑΤΟΛΟΓΙΟ

Βιολογική Διατροφή και Υγεία Παιδιών

**Ονοματεπώνυμο παιδιού**			
**Ονοματεπώνυμο γονέα**			
**Τηλέφωνο επικοινωνίας (κινητό)**			
**Κωδικός ερωτηματολογίου**		**Ημερομηνία**	

Μέρος Α

Παρακαλώ όπως απαντήσετε τις ακόλουθες ερωτήσεις:

1. Μορφωτικό επίπεδο:

	**Μητέρα**	**Πατέρας**
Δημοτικό	☐	☐
Γυμνάσιο-Λύκειο	☐	☐
Πανεπιστήμιο-Κολλέγιο	☐	☐
Μεταπτυχιακό-Διδακτορικό	☐	☐
Άλλο ……………………………………	☐	☐

2. Εργασία μητέρας: ☐
3. Εργασία πατέρα: ☐
4. Πόλη/χωριό διαμονής: ☐
5. Ταχυδρομικός κώδικας: ☐
6. Χρόνος διαμονής στην παρούσα κατοικία: ☐ μήνες/χρόνια

7. Πόσο συχνά χρησιμοποιήσατε φυτοφάρμακα στην οικία σας (εσωτερικό σπιτιού και εξωτερικό σπιτιού-κήπος, γρασίδι, δέντρα) τους τελευταίους 3 μήνες; Όπου φυτοφάρμακα αναφερόμαστε σε ζιζανιοκτόνα ή εντομοκτόνα ή εντομοαπωθητικά ή μυκητοκτόνα ή ποντικοφάρμακα.

	**Εξωτερικό σπιτιού**	**Εσωτερικό σπιτιού**	
Εντομοαπωθητικά			Φορές /βδομάδα/μήνα
Εντομοκτόνα			Φορές /βδομάδα/μήνα
Μυκητοκτόνα			Φορές /βδομάδα/μήνα
Ζιζανιοκτόνα			Φορές /βδομάδα/μήνα
Ποντικοφάρμακα			Φορές /βδομάδα/μήνα

Αν δεν χρησιμοποιείτε φυτοφάρμακα, παρακαλώ όπως συνεχίσετε με το Μέρος Β.

8. Πότε έγινε η πιο πρόσφατη χρήση φυτοφαρμάκων στην οικία σας (μέρες, εβδομάδες, μήνες κλπ);

	**Εξωτερικό σπιτιού**	**Εσωτερικό σπιτιού**
Εντομοαπωθητικά		
Εντομοκτόνα		
Μυκητοκτόνα		
Ζιζανιοκτόνα		
Ποντικοφάρμακα		

9. Γνωρίζετε ποια φυτοφάρμακα χρησιμοποιείτε συνήθως στην οικεία σας; Παρακαλώ ονομάστε τα.

<u>Μέρος Β</u>

Παρακαλώ όπως απαντήσετε τις ακόλουθες ερωτήσεις για το παιδί σας:

1. Φύλο παιδιού: Αγόρι ☐ Κορίτσι ☐
2. Ημερομηνία γέννησης παιδιού: ☐
3. Έχει το παιδί σας τη συνήθεια να βάζει το χέρι στο στόμα του (ονυχοφαγία, πιπίλισμα δακτύλου);

   Ναι ☐ Όχι ☐

4. Πόσο χρόνο περνά το παιδί σας σε πάρκα και κήπους σε εβδομαδιαία βάση;

   Κήπος: ☐ ώρες/εβδομάδα

   Πάρκο: ☐ ώρες/εβδομάδα

5. Πόσο χρόνο αφιερώνει το παιδί σας σε αθλοπαιδιές, σε εβδομαδιαία βάση;

	**Ώρες/εβδομάδα**
Τρέξιμο	
Ποδηλασία	
Καλαθόσφαιρα	
Ποδόσφαιρο	
Πετόσφαιρα	
Κολύμπι	
Χορός	
Άλλο ……………………..	

6. Πόσο χρόνο αφιερώνει το παιδί σας σε καθιστικές δραστηριότητες σε εβδομαδιαία βάση;

	**Ώρες/εβδομάδα**
Τηλεόραση	
Υπολογιστής	
Tablet	
Κινητό	
Άλλο ………………………..	

<u>Μέρος Γ</u> (Θα συμπληρωθεί από μέλος της ερευνητικής ομάδας)

**Ημερομηνία**	**Αρχή μελέτης (…./…./…….)**	**Τέλος μελέτης (…./…./…….)**
**Βάρος**		
**Ύψος**		
**Περιφέρεια μέσης**		
