# Supplementary material for: A cluster-randomized crossover trial of organic diet impact on biomarkers of exposure to pesticides and biomarkers of oxidative stress/inflammation in primary school children
Source: PLoS One. 2019 Sep 4;14(9):e0219420. doi: 10.1371/journal.pone.0219420 (PMC6726134; doi:10.1371/journal.pone.0219420)
Supplement: S4 Text — (DOCX) [file pone.0219420.s006.docx]

BioAnalytical Protocols for

A cluster-randomized crossover trial of organic diet for primary school children

*Konstantinos C. Makris^1*^, PhD, Corina Konstantinou^1^, MS, Xanthi D. Andrianou^1^, MS, Pantelis Charisiadis^1^, PhD, Alexis Kyriacou^2^, RD, Matthew O. Gribble^3,4^^, PhD DABT, and Costas A. Christophi^1^^, PhD*

^1^ Cyprus International Institute for Environmental and Public Health, Cyprus University of Technology, Limassol, Cyprus

^2^ Faculty of Health Sciences and Sport, University of Stirling, Stirling, UK

^3^ Department of Environmental Health, Emory University, Atlanta, GA, USA

^4^ Department of Epidemiology, Emory University, Atlanta, GA, USA

^ Both authors contributed equally to this work.

* Corresponding author: Konstantinos Christos Makris, Associate Professor of Environmental Health, Cyprus International Institute for Environmental and Public Health, School of Health Sciences, Cyprus University of Technology, Limassol, Cyprus.

Phone: 357-25002398, FAX: 357-25002676

E-mail: [konstantinos.makris@cut.ac.cy](mailto:konstantinos.makris@cut.ac.cy) (KCM)

Table of Contents

[Pesticides Protocol 3](#_Toc535949210)

[Malondialdehyde Determination in Urine: Analytical Protocol 6](#_Toc535949211)

[References 12](#_Toc535949212)

## Pesticides Protocol

**This is a published biomonitoring protocol in Analytical and Bioanalytical Chemistry, 2019, pp 1–9, Cohort-friendly protocol for the determination of two urinary biomarkers of exposure to pyrethroids and neonicotinoids using gas chromatography-triple quadrupole mass spectrometry.** **https://doi.org/10.1007/s00216-019-01925-9**

**Authors: Pantelis CharisiadisThibaut Delplancke, and Konstantinos C. Makris**

**Materials and Methods**

Hydrochloric acid 37%, acetone, methanol and methyl tert-butyl ether (MTBE) were purchased from Merck. Acetonitrile GC grade and sodium sulphate from Aldrich. 2-phenoxybenzoic acid (2-PBA), 3-phenoxybenzoic acid (3-PBA) and 6-chloronicotinic acid (6-CN) were purchased from Aldrich. Toluene from BDH and bis-(trimethylsilyl)trifluoroacetamide (BSTFA) from Supelco.

**Standard and working solutions**

Stock solution of 50 mg L^−1^ in acetonitrile for each of the 6-chloronicotinic acid (6-CN) and 3-phenoxybenzoic acid (3-PBA) was prepared from the initial concentration of 1000 mg L^−1^, and further diluted to prepare calibration and additive solutions in acetonitrile. A 2-phenoxybenzoic acid (2-PBA) solution of 50.0 mg L^−1^ in acetonitrile was prepared (50 mg in 1000 mL) and further diluted for the working solution of 5.0 mg L^−1^. All solutions containing pesticides are stored always at −20 °C in GC glass vials.

**Preparation of the Calibration Solutions**

Calibration solutions of 6-chloronicotinic acid (6-CN) and 3-phenoxybenzoic acid (3-PBA) in acetonitrile were used at eleven different concentrations to obtain the calibration curve. One single solution of 6-CN and 3-PBA was prepared at 1,500 mg L^−1^ concentration in acetonitrile from each stock solution. Serial dilutions for 11 calibration points, starting from the highest concentration to the lowest were prepared from the working concentration of the two pesticides.

**Sample preparation**

The urine samples to be analysed were removed from the freezer -80°C and thawed in room temperature. Our methodology was based upon the principles of the previous published methodologies^1,2^ after several modifications. In detail extraction protocol was optimized by mixing 2mL urine sample and following the sequence of the next steps. Addition of 20μL 2-phenoxybenzoic acid (internal standard) and addition 0.5mL of HCl and incubation at 90 C for 2h. After the samples were cooled in room temperature and 2mL of MTBE was added, following 15 min in a lab shaker at 150 rpm and then centrifugation for 2 min at 3000 rpm. 1mL of the upper phase was transferred into a GC autosampler screw top glass vial and dried under gas nitrogen. Addition of 150μL toluene, followed by 1min vortex and 50μL BSTFA was added in each sample. A 200μL portion of sample transferred in a glass insert placed in a GC autosampler screw top glass vial and then injected to the instrumentation for GC-MS analysis.

**GC-MS/MS analysis**

GC-MS spectra were recorded on an Agilent 7890A GC equipped with an Agilent 7000B triple quadrupole MS detector. Compounds were separated on an Rxi-5ms (5% diphenyl/95% dimethylpolysiloxane) column from Restek (30 m × 250 μm × 0.25 μm) and helium carrier gas (99.999%) flow was maintained at 1.0 mL min^-1^. The inlet temperature was set at 140 °C for 0.1 min then ramped to 300 °C at a rate 300 °C min^-1^ where it was maintained. The oven was set to 30 °C for 5min, ramped to 100 °C at a rate 50 °C min^-1^ where it was maintained for 2.4 min, then ramped to 260 °C at a rate 120 °C min^-1^ where it was maintained for 1.6 min followed by a post run period at 260 °C for 1 min. The total run time was 20 min for each sample. The injection volume was 2μL, and the injection syringe was washed 1 time with toluene and 1 with acetone before and after sample injection. MSD transfer line and MS source temperatures were held at 250 °C, while quadruples were held at 150 °C. Mass spectra were obtained using electron impact ionization (70 eV) in the multiple reaction monitoring (MRM) mode in 3 per second scanning cycles, with a solvent delay of 4.0 min. The system was controlled by the software Mass Hunter Workstation (Agilent, rev. B.05.00).

**Calibration curve and linearity**

To eliminate any matrix effects and to reduce endogenous levels of the analytes of interest, procedural calibration standards were prepared in pooled urine sample with negligible pesticides measured concentrations diluted. An aliquot of 2mL from the pooled urine sample was placed in a 7mL screw top glass vial with screw cap. The desired concentration of dilution standard solution was added. The curve was established by measuring eleven samples of urinary pesticides at final concentration from 0.030 to 13.7009 μg L^−1^ using GC-MS/MS in the MRM mode. Quantitative analysis was based on peak area measurements as ratios versus peak area of internal standard. Peaks were automatically integrated using the instrument software; the analyst checked and corrected any discrepancies in peak selection yielding an accurate integration. All samples were treated as following the sample preparation procedure.

**Method detection limit**

The limit of quantification (LOQ) and limit of detection (LOD) were determined based on the standard deviation of nine measurements of the lowest concentrations level of the calibration curve. The limit of detection (LOD) was calculated by adding 3× the standard deviation of the response. Accordingly, the limit of quantification (LOQ) was equal to 3× the LOD. The limit of detection (LOD) for 6CN and 3PBA is 0.075 μg L^−1^ and 0.049 μg L^−1^, respectively.

**Quality Assurance-Quality Control**

A 10% of quality controls and checks were placed in each batch of the analysis to ensure quality assurance and quality control safeguards of the method. Urine samples were spiked with a solution of the targeted analytes at final concentration of 1.7 μg L^-1^, 2.4 μg L^-1^ and 4.8 μg L^-1^, and their recoveries in unknown samples were calculated. Blanks were properly used in each batch. The recoveries values for 6CN and 3PBA were 97.9%, 96.1%, and 111.9%, 95.7%, 99.8%, and 83.0%, respectively. The intra-day variability for 6CN and 3PBA at the specific concentration levels were 3.1%, 7.7%, and 10.4%, 4.6%, 3.3%, and 1.7%, respectively. The inter-day variability for 6CN and 3PBA at the specific concentration levels were 7.2%, 6.8%, and 9.3%, 6.8%, 5.8%, and 4.3%, respectively. The average recovery of the internal standard during all samples analysis was 84.4% plus/minus of about 6%.

## Malondialdehyde Determination in Urine: Analytical Protocol

**Description**

TBARS (Thiobarbituric Acid Reactive Substances) is one of the methods for determination of lipid peroxidation. The principle of this method is based on the reaction of malondialdehyde with thiobarbituric acid in acidic conditions (pH ≈ 1-2) and at a high temperature (T = 90°C) to form a pink MDA-TBA complex, which can be quantified spectrophotometrically at 532 nm.


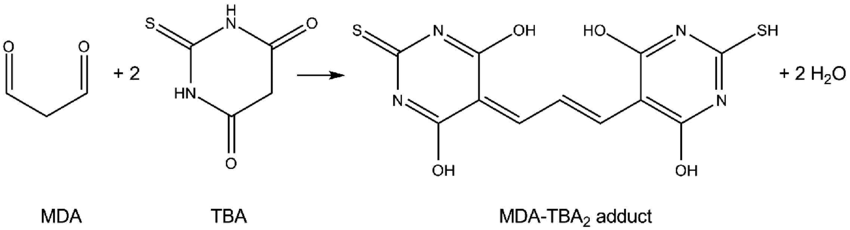


Figure 2. Reaction of malondialdehyde (MDA) with 2 molecules of 2-thiobarbituric acid (TBA)^3^

The protocol is based on a previously described method with modifications^3^. The modifications are listed below:

|  | **This protocol** | **Weitner et al., 2016** |
| --- | --- | --- |
| **Spectrophotometer equipment** | Microplate (96-well plates) | Quartz cuvettes |
| **Centrifugation** | 6000 rpm for 5 min | No |
| **MDA stock solution storage** | +4°C | -20°C |
| **Working solutions concentration (μΜ)** | 0.28 – 17.76 | 0.1 – 10.11 |
| **Urine sample volume (μL)** | 100 | 400 |
| **Heating duration (min)** | 45 | 30 |
| **Heating instrument** | Oven | Temperature controlled heating block |
| **Reaction stopping procedure** | Placing of samples in -20°C for 15 min | Placing of samples on ice |
| **Measurement wavelength (nm)** | 540 | 532 |
| **Determination of MDA concentration** | Calibration curve | Standard addition procedure |
| **No of samples for each urine sample** | 2 replicates | 4 (blank, test, and 2 with added known concentration  of MDA at two levels) |

**Chemicals**

- 2-thiobarbituric acid (TBA) (Sigma-Aldrich)
- o-phosphoric acid (o-H_3_PO_4_) (BDH Chemicals)
- 1,1,3,3-tetramethoxypropane (Sigma-Aldrich)

**Instrumentation**

- Photometer microplate reader Sunrise model by Tecan Group LTD, controlled by the Magellan Tecan version 7.1 software
- Vortex V1 plus (Boeco, Germany)
- Centrifucator Biocen 20 (Orto Alresa)
- Oven (Memmert)
- Freezer -20°C

**Solutions**

- **2-thiobarbituric acid (TBA, 0.6%)**

A 100 mL volumetric flask is used within which 0.6122 g of 2-thiobarbituric acid (TBA) in a pellet form are pre-dissolved with approximately 50 mL bidistilled water. The solution is heated and mixed and the volumetric flask is subsequently filled to its nominal volume bidistilled water. The solution is stable in room temperature for a month.

- **O-phosphoric acid (o-H_3_PO_4_, 1%)**

In a 100 mL volumetric flask, 1.18 mL of O-phosphoric acid (o-H_3_PO_4_) is transferred and filled with bidistilled water up to its nominal volume. The solution is stable in +4°C for a month.

- **TMP interim solution (1, 1, 3, 3-tetramethoxypropane, 200 mg/L)**:

In a 100 mL volumetric flask, 201 μL of 1, 1, 3, 3-tetramethoxypropane (MDA) is transferred and filled with bidistilled water to its nominal volume. The solution is stable in +4°C for a month.

- **TMP Stock Solution (1,1,3,3-tetramethoxypropane, 2 mg/L):**

In a 1000 mL volumetric flask, 10 mL of TMP interim solution (200 mg/L) is transferred and filled with bidistilled water to its nominal volume. The solution is stable in +4°C for a month.

- **TMP calibration solutions**

The table indicates the TMP stock solution dilutions prepared with bidistilled water in 1.5 mL centrifuge tubes. The calibration standards are prepared fresh daily.

**Table 1**. Pipetting scheme for the preparation of the calibration standards solutions in water.

| Concentration of the calibration standard solution (μg/L) | Concentration of the calibration standard solution (μMol/L) | Final Volume of the calibration standard solution (mL) | Volume of TMP stock solution (mL) |
| --- | --- | --- | --- |
| 20 | 0.28 | 1.5 | 0.015 |
| 40 | 0.56 | 1.5 | 0.03 |
| 80 | 1.11 | 1.5 | 0.06 |
| 160 | 2.22 | 1.5 | 0.12 |
| 320 | 4.44 | 1.5 | 0.24 |
| 640 | 8.88 | 1.5 | 0.48 |
| 1280 | 17.76 | 1.5 | 0.96 |

**Specimen Collection and Storage**

First morning urine samples were collected from children participating in the “Organic diet and children’s health study”, which is implemented under the ORGANIKO project. Our sampling team collected from schools, the children’s urine samples in a 60 mL polypropylene vial with a minimum headspace and immediately transferred in a portable ice bag. Upon arrival in the laboratory the samples were stored in the −80 °C freezer until analysed. 1.2 ml from each sample was aliquoted into 1.5 mL centrifuge tubes.

**Sample preparation**

The frozen urine samples in the 1.5 mL centrifuge tubes are thawed and shaken briefly in the vortex before centrifuged at 6000 rounds per minute for 5 minutes. 100 μL of the supernatant are transferred to a new 1.5 mL centrifuge tube.

**Procedure**

Pooled urine sample without the addition of TBA serves as the blank. For quality control purposes, three samples are used:

1. S: 100 μL of pooled urine sample
2. S60: 5 μL of 1280 μg/L TMP standard solution are added in 100 μL of pooled urine sample (final concentration = 61 μg/L).
3. S80: 5 μL of 1680 μg/L TMP solution are added in a 100 μL of pooled urine sample (final concentration = 80 μg/L). The 1680 μg/L TMP solution is prepared by adding 8.4 mL of TMP stock solution and filled with bidistilled water up to 10 mL.

Analytical steps:

1. 100 μL of urine sample/standard/blank/quality control are placed in a 1.5 mL centrifuge tube.
2. 250 μL of TBA (0.6%) are added in all tubes except the blank.
3. 750 μL of o-H3PO4 (1%) are added in all tubes.
4. The reaction mixture is heated at 90°C for 45 min in the oven.
5. Reaction is stopped by placing the samples in the freezer (-20°C) for 15 min.
6. 250 μL of the mixture are applied in the 96-well plate based on the template (Table 2). Each sample/ standard/blank/quality control is analyzed in duplicate.
7. Samples absorbance is measured at 540 nm wavelength.

Table 2. Template of a 96 well-plate.

|  | **1** | **2** | **3** | **4** | **5** | **6** | **7** | **8** | **9** | **10** | **11** | **12** |
| --- | --- | --- | --- | --- | --- | --- | --- | --- | --- | --- | --- | --- |
| **A** | Blank | Blank | 20 | 20 | 40 | 40 | 80 | 80 | 160 | 160 | 320 | 320 |
| **B** | 640 | 640 | 1280 | 1280 | Sample | Sample | Sample | Sample | Sample | Sample | Sample | Sample |
| **C** | Sample | Sample | Sample | Sample | Sample | Sample | Sample | Sample | Sample | Sample | Sample | Sample |
| **D** | Sample | Sample | S60 | S60 | S80 | S80 | Sample | Sample | Sample | Sample | Sample | Sample |
| **E** | Sample | Sample | Sample | Sample | Sample | Sample | Sample | Sample | Sample | Sample | Sample | Sample |
| **F** | Sample | Sample | Sample | Sample | Sample | Sample | S | S | S60 | S60 | S80 | S80 |
| **G** | Sample | Sample | Sample | Sample | Sample | Sample | Sample | Sample | Sample | Sample | Sample | Sample |
| **H** | Sample | Sample | Sample | Sample | Sample | Sample | Sample | Sample | Sample | Sample | Blank | Blank |

**MDA Concentration Calculation**

The MDA concentration is based on the calibration curve included at every well-plate. All samples/standards/quality controls absorbance is corrected by subtracting the average of the blank absorbance. For constructing the calibration curve, the mean of the duplicates of the standards is calculated and plotted as a function of the corresponding concentrations (see example of a curve at Fig.1 and quantitative data of the curve at Table 3).

Figure 2. Calibration curve of the creatinine analysis in urine samples.

Table 3. Quantitative data of the calibration curve from Figure 1.

| **Analyte** | **Equation** | **R2** | **LOD (μg/L)** | **LOQ (μg/L)** |
| --- | --- | --- | --- | --- |
| Malondialdehyde | y = 0.0005 x - 0.0002 | 0.9994 | 13 | 43 |

**Quality Control Recovery**

At the end of each analysis interval, the recoveries of the calibration curve were calculated. Quality control solutions were prepared as aforementioned and fortified with the desired concentrations of standard compounds. The percent recovery, R, was calculated for the concentration of each as shown below:

R = [(A-B)/C] x 100

Where:

R = Recovery %

A = Fortified measurement of S60/S80

B = Background measurement of S

C = Fortifying concentration (for S60 = 61 μg/L and for S80 = 80 μg/L)

The recoveries of the analyte being determined must fall between 80% and 120%.

For the LOD and LOQ determination, ten calibration curve solutions of the low limit of the curve, were prepared and measured as samples. LOD was then calculated by multiplying the standard deviation of these ten samples concentration by a factor of 3 and for LOQ, by multiplying it by a factor of 10.

## References

1. Leng G, Gries W. Determination of Pyrethroids in Blood Plasma and Pyrethroid/ Pyrethrin Metabolites in Urine by Gas Chromatography– Mass Spectrometry and High-Resolution GC–MS. :19.

2. Nomura H, Ueyama J, Kondo T, et al. Quantitation of neonicotinoid metabolites in human urine using GC-MS. J Chromatogr B 2013;941:109–15.

3. Weitner T, Inić S, Jablan J, Gabričević M, Domijan A-M. Spectrophotometric Determination of Malondialdehyde in Urine Suitable for Epidemiological Studies. Croat Chem Acta 2016;89(1):133–9.
