# Supplementary material for: A cluster-randomized crossover trial of organic diet impact on biomarkers of exposure to pesticides and biomarkers of oxidative stress/inflammation in primary school children
Source: PLoS One. 2019 Sep 4;14(9):e0219420. doi: 10.1371/journal.pone.0219420 (PMC6726134; doi:10.1371/journal.pone.0219420)
Supplement: S5 Text — (DOCX) [file pone.0219420.s007.docx]

**Statistical Analysis Plan (SAP)**

**A cluster-randomized crossover trial of organic diet for primary school children**

*Konstantinos C. Makris^1*^, PhD, Corina Konstantinou^1^, MS, Xanthi D. Andrianou^1^, MS, Pantelis Charisiadis^1^, PhD, Alexis Kyriacou^2^, RD, Matthew O. Gribble^3,4^^, PhD DABT, and Costas A. Christophi^1^^, PhD*

^1^ Cyprus International Institute for Environmental and Public Health, Cyprus University of Technology, Limassol, Cyprus

^2^ Faculty of Health Sciences and Sport, University of Stirling, Stirling, UK

^3^ Department of Environmental Health, Emory University, Atlanta, GA, USA

^4^ Department of Epidemiology, Emory University, Atlanta, GA, USA

^ Both authors contributed equally to this work.

* Corresponding author: Konstantinos Christos Makris, Associate Professor of Environmental Health, Cyprus International Institute for Environmental and Public Health and School of Health Sciences, Dean, Cyprus University of Technology, Limassol, Cyprus.

Phone: 357-25002398, FAX: 357-25002676

E-mail: [konstantinos.makris@cut.ac.cy](mailto:konstantinos.makris@cut.ac.cy) (KCM)

Table of Contents

[Introduction 3](#_Toc517440282)

[Study design 3](#_Toc517440283)

[General aspects 3](#_Toc517440284)

[Objectives 4](#_Toc517440285)

[Inclusion and exclusion criteria 4](#_Toc517440286)

[Inclusion criteria for clusters 4](#_Toc517440287)

[Inclusion criteria for participants 4](#_Toc517440288)

[Exclusion criteria for participants 4](#_Toc517440289)

[Randomization and blinding 4](#_Toc517440290)

[Recruitment process 5](#_Toc517440291)

[Intervention 6](#_Toc517440292)

[Adherence 7](#_Toc517440293)

[Adherence of organic meals provider 7](#_Toc517440294)

[Adherence of participants to the organic diet 7](#_Toc517440295)

[Flow of schools and children 8](#_Toc517440296)

[Withdrawals 8](#_Toc517440297)

[Outcomes 8](#_Toc517440298)

[Sample size 9](#_Toc517440299)

[Statistical analysis 9](#_Toc517440300)

[General principles 9](#_Toc517440301)

[Participant population 9](#_Toc517440302)

[Levels of confidence and p values 10](#_Toc517440303)

[Unadjusted and adjusted analyses 10](#_Toc517440304)

[Multiple testing 10](#_Toc517440305)

[Handling of missing data and data below LOD 10](#_Toc517440306)

[Baseline characteristics 11](#_Toc517440307)

[Conventional period energy analysis 11](#_Toc517440308)

[Change between baseline and the end of the organic period 12](#_Toc517440309)

[Overall change between conventional and organic period 12](#_Toc517440310)

[Primary analysis of outcomes 12](#_Toc517440311)

[Post-hoc analysis 13](#_Toc517440312)

[Sensitivity analysis 13](#_Toc517440313)

[Estimation of the geometric mean ratios and odds ratios 13](#_Toc517440314)

[References 14](#_Toc517440315)

[SAP Appendix 15](#_Toc517440316)

# Introduction

The purpose of this SAP is to define and describe the final analysis of the ORGANIKO LIFE+ study data.

# Study design

## General aspects

The ORGANIKO LIFE+ study is an investigator-initiated 2 x 2 cluster-randomized, crossover, blinded outcome assessed trial to evaluate the effect of an organic diet intervention on biomarkers of exposure (pesticides metabolites) and biomarkers of effect (oxidative stress/inflammation markers) in children aged 10-12 years, in Cyprus. The trial was conducted in six primary schools in two periods in Limassol, Cyprus, during October 2016-April 2017. The trial was designed by the Cyprus International Institute for Environmental and Public Health, and neither the funder (EU LIFE+ programme) nor the sponsoring companies of organic products were involved with the design or conduct of the trial. The sponsors were also not involved in the data collection or analysis, the writing of the manuscript, nor the decision to submit it for publication. The trial protocol was approved by the Cyprus National Bioethics Committee (ΕΕΒΚ/ΕΠ/2016/25) and the Cyprus Ministry of Education and Culture (7.15.06.15/2). The trial was registered (ClinicalTrials.gov identifier NCT02998203) and was performed in accordance with the principles of the Declaration of Helsinki.

The reasons for the cluster randomization were: (1) to avoid the transfer of knowledge within schools about the organic diet intervention from children randomized in the intervention arm to children randomized in the conventional arm (contamination effect); (2) to enhance compliance, since all participating children at a given school would follow the same intervention; (3) to ensure more efficient delivery of the organic meals during the organic period since the organic meals-preparing restaurant would daily deliver meals to 3 instead of 6 schools.

The reasons for choosing the crossover design were the following: (1) each participant serves as his/her own control and the influence of confounding variables (i.e., variables that are imbalanced between the treatment and control groups) is reduced; (2) repeated measures over a longitudinal study reduce the chance for a study finding to be attributable to chance under temporal measurement error. One organic-certified restaurant was responsible for the preparation and provision of organic meals for all schools during the organic period helping ensure that the treatment was comparable per the stable unit treatment value assumption (SUTVA). Informed consent was obtained at school level by the headmaster of the participating school, followed by written informed consent that was obtained from each participating children’s parents or legal guardians.

## Objectives

The primary objective of the ORGANIKO LIFE+ trial was to determine the effectiveness of an organic diet intervention in reducing the body burden of urinary concentrations of pyrethroid and neonicotinoid pesticide metabolites in children aged 10-12 years in Cyprus.

The secondary objective of this trial was to evaluate the effect of an organic diet intervention on urinary biomarkers of oxidative stress/inflammation (OSI).

## Inclusion and exclusion criteria

### Inclusion criteria for clusters

Public primary schools within the urban area of Limassol.

### Inclusion criteria for participants

Healthy children (males and females) at the age of 10-12 years old, attending public primary schools in Limassol, residing in Cyprus at least for the last five years and systematically consuming conventional food (>80% of the week’s meals).

### Exclusion criteria for participants

Children with chronic conditions (e.g. diabetes, asthma) or allergies in food (e.g. gluten, lactose tolerance).

## Randomization and blinding

The unit of randomization was the school. Schools were randomly assigned following simple randomization procedures of no restriction or matching (computerized random numbers) to 1 of 2 groups depending on the sequence of the treatments (Group 1 and Group 2). Simple randomization was performed by a computer generated random list that was prepared by an investigator with no clinical involvement in the trial. A member of the research team enrolled participants after knowing the allocation sequence for each school since parents and children had to be informed of the order the intervention was going to take place.

Participants of schools in Group 1 began first with the organic period and continued with the conventional period whereas participants of schools in Group 2 began first with the conventional period and then switched to the organic period.

The blinding of the participants to group assignment was not possible with the present study design. The blinding of the researchers to the subjects’ identity was achieved by the coding of all study materials (urine containers, questionnaires, and diaries). The study personnel who obtained the outcome measurements were also not informed of the group assignment. The personnel who delivered the intervention did not take any outcome measurements. All outcome assessors and data analysts were kept masked to the allocation.

## Recruitment process

Following the bioethics approval of the study, twelve primary schools’ headmasters were randomly selected and contacted by the study coordinator. Six schools accepted to implement the study.

Informational flyers were offered to parents and information meetings were arranged to present study-relevant details and to provide additional study materials for their review (menus, details of study, interest forms, consent forms for the parents/guardians of the participating child, and forms for breakfast and afternoon snack options). Parents that could not attend the meeting received the relevant material at home through the schools. Similar meetings, although using different study materials, were arranged with children in each school to inform them about the study, to explain to them the definition of organic food products (raw and processed), and to have them taste organic fruits. The importance of compliance to the study protocol was highlighted to both parents and children and the ways to report it in a diary, including examples of proper use. The research team checked if all eligibility criteria were satisfied, using the completed interest forms, and parents were accordingly informed via telephone. Following, informed consents were signed by parents of the children that were willing to participate. Bags containing six coded urine vials, sampling dates, instructions for urine collection, and the food diary were given to children or to their parents during school hours. The organic restaurant was selected based on a public tender procurement procedure with some of the requirements being the use of 100% certified organic raw materials and food products, frequently renewed certificate for preparation of organic meals, and national accreditation for delivery of meals to schools, complying to ISO 22000 and HACCP protocols.

## Intervention

During the conventional period, participants were asked to maintain their usual dietary habits and choices (>80% conventional diet) for a total of maximum 40 days. The organic period was organized in two 20-day cycles with differences in the menu to allow for greater variety in the meals, so that the participants’ compliance to the organic diet would be enhanced. During the organic period, participants were asked to strictly consume the two organic dietary menus provided to them for a total of 40 days. The organic dietary menus were prepared by a certified dietitian based on EFSA energy requirements for children aged 10-12 years,^1^ and included five meals per day; breakfast, morning snack, lunch, afternoon snack, and dinner. The meals of the organic period were delivered to schools Monday to Saturday, except for Sunday. Upon arrival of daily meals to each school, the teachers and headmaster were responsible for handing the packages to each participating child, using a project participant list around lunch time. On Saturday, the food package included both the Saturday and Sunday meals; for the Sunday meals, the foods were raw so that the parents would cook them fresh on Sunday. The meals of lunch and dinner were the same for all participants. For the breakfast, morning and afternoon snacks, children could choose based on a list of available products before the beginning of the organic period. Participants crossed over to the alternate diet on the following day after the first period was completed. A washout period was not required; it was intrinsically included in the two periods, since the first urine sample of the second period was collected about 12 days after the beginning of the second period and the pesticides half-lives are short (half-life for pyrethroids is less than 12 h and those for neonics range from 5-33 hours), so no carryover effect was expected.^2.3^

Each participant provided maximum six first morning urine samples during the whole duration of the 2-period study; one baseline sample, two samples in the conventional period, and three samples in the organic period. Anthropometric measurements (weight, height, and waist circumference) were taken at the beginning of the study, at the end of the organic period, and at the end of the study (for Group 2, the end of study and end of organic period was the same time point) by trained researchers at the school premises.^4^ A baseline questionnaire was administered to parents at the beginning of the study through a telephone interview to collect information on demographic characteristics, the child’s lifestyle/behaviour habits, and possible non-dietary pesticide exposure sources. A food frequency questionnaire was administered to the parents at the end of the conventional period through a telephone interview to collect information about the food habits of the children during the conventional period. A food diary was provided to the parents at the beginning of the study and parents completed it during the organic period, in order to collect information about the compliance of the children to the organic dietary menu.

## Adherence

### Adherence of organic meals provider

There was only one provider of organic meals to schools, hence the same raw organic materials/ingredients and products, preparation of meals, and delivery of organic packages were applied to all participants. The research team was in close communication with the organic meal provider and delivery team to ensure that the meals preparation and menu options for each day were strictly followed based on the 20-day organic menu cycle. The research team was conducting frequent checks in school during meal delivery hours and during organic meal hand out to participants to ensure adherence to the menu. The team had also weekly visits to each school to interact with student participants asking their opinion on the food choices and the menu quality. Children and parents’ opinions about the menu were routed back to the organic restaurant to enhance the adherence of the menu protocol to the participant needs. The participants’ parents were given access to a hotline that was referring them to the trained researcher for communicating any issues with their meals; additional daily communication with the restaurant ensured that a full cycle of quality control and participant opinions were feeding back into the standardised protocol of the organic period.

### Adherence of participants to the organic diet

The adherence of participants to the organic diet intervention was assessed with a food diary in which parents were instructed to record any exceptions to the organic diet. Only consumption of conventional food products was noted in the diary. If a child would consume additional organic meals or snacks, or a smaller part of the provided meals, it was not requested to include it in the dairy. Moreover, the diary was used to note any sickness and any pesticide use at home. The frequent telephone communication with parents, the weekly visits at schools, and the daily communication with the restaurant were key factors that further enhanced the compliance to the organic diet treatment. In order to encourage adherence to the organic diet until the end of the period, a public event was organized during the mid-period of the study with activities for the participating children and free sampling of organic food products.

## Flow of schools and children

The flow of schools and children through the trial is reported in accordance with the CONSORT extension statement for cluster trials.^5^ The flow diagram includes the number of eligible and recruited schools, number of eligible and recruited children and then, by allocated group, the number of children who completed the trial, the number lost to follow-up and the numbers included in the analysis.

## Withdrawals

Parents could choose to withdraw their children from the study at any point of the organic diet intervention without having to provide any explanation. Children who discontinued completing the data collection prior to the end of the trial period were withdrawn but their already collected data remained available for analysis, unless parents had requested otherwise. Reasons for withdrawal were documented wherever possible.

## Outcomes

Per the trial protocol, the primary outcomes are the biomarkers of exposure to pesticides using two non-specific pesticides metabolites (3-phenoxybenzoic acid (3-PBA), 6-chloronicotinic acid (6-CN)) measured in first morning urine voids collected any two up to six time points. The secondary outcomes are the biomarkers of oxidative stress/inflammation (8-iso-prostaglandin F2a (8-iso-PGF2a), malondialdehyde (MDA) and 8-hydroxy-2′-deoxyguanosine (8-OHdG)) measured in the same urine samples.

## Sample size

To estimate the sample size, the PS software for calculating sample size was used.^6^ We used the paired t test study design for calculating the power of this test, assuming that each subject has a pair of values (one for conventional and one for organic diet). The variable used for the estimation of the sample size was a biomarker of inflammation (C-reactive protein), because the biomarkers of inflammation/oxidative stress were one the primary outcomes for the trial and we information on pesticide levels were unavailable at that time. The lack of pertinent human studies with similar setting and characteristics to the present study that involved exactly the same biomarkers of effect (8-OHdG and isoprostanes) led us to use the available human study of CRP.^7^

The input parameters were: significance level, within patient standard deviation or standard deviation of the difference between the two values for the same patient, power and minimal detectable difference in means. Based on the literature, the within subject standard deviation of CRP was assumed to be 0.5,^7^ and we hypothesized a minimal detectable difference in biomarkers of effect (CRP) between the organic and conventional treatment to be 0.1, based on other intervention studies.^8–10^ Assuming a power of 80% and a two-sided 5% significance level, the sample size for this two-treatment crossover study was estimated to be 200 children. The clustering was not addressed, but the within cluster within period and the within cluster between period intra cluster correlation estimates were assumed equal.

# Statistical analysis

## General principles

### Participant population

The analysis population consists of all children excluding withdrawn children whose parents also withdrew their consent for the data to be used or children who left the trial with less than 12 days in the organic diet arm and did not provide any samples during the organic diet intervention.

Whilst it was not anticipated that there would be children who cross-over their trial group (i.e. change from intervention to control, or vice versa), any two exceptions were documented. Two children (following their parents’ signed consent) decided to enroll in the study after the study was initiated and at the end of the conventional arm, right before the beginning of the organic diet treatment, based on their school’s group (Group 2). These children provided a baseline sample, 2 or 3 samples during the organic period and 2 conventional samples after the organic diet period ended, so they basically followed the opposite design based on their school. However, they did follow the organic period at the same time as their classmates. These two children were allocated to Group 1 for analysis purposes. There was no issue of transferring knowledge within the school about the organic diet intervention from these two children since they were participating in the intervention arm at the same time the rest children of the school were. The only difference was that these two children participated in the conventional arm after the end of the organic diet and not before the beginning of the organic diet treatment, as the rest of the children of their respective school.

The full analysis population consists of all children who followed the organic treatment for at least 12 days and provided at least one urine sample during the organic diet intervention.

### Levels of confidence and p values

Statistical tests and confidence intervals were two-sided. 95% confidence intervals will be presented wherever possible. The statistical significance level set will be at the 5% level.

### Unadjusted and adjusted analyses

All outcomes were used with for creatinine levels to account for urine dilution. If outcomes are not normally-distributed and the analyses require the normality assumption, then outcomes will be log-transformed (natural logarithm).

### Multiple testing

Multiple testing will be accounted for, using the Benjamini-Hochberg (false discovery rate, FDR) method considering all the regression parameters of the primary and post-hoc analyses. Q-value <0.05 indicates statistical significance of an association after controlling the false discovery rate at 5%.

### Handling of missing data and data below LOD

Missing values in the questionnaire were not imputed. For the biomarkers data below the limit of detection (LOD), imputation was performed based on existing suggestions for handling non-detect urinary measured concentrations data.^11^ For outcomes that contained <20% values below the detection limit, values <LOD were imputed as LOD/2 and for outcomes that contained ≥20% values below detection, values <LOD were imputed with regression on order statistics (ROS). With the ROS method, non-detect data are replaced based on a probability plot of detects. Outcomes with >70% values below detection, were used as binary variables in the primary, post-hoc and sensitivity analyses.

## Baseline characteristics

The baseline characteristics were summarized overall and by study group. These include: demographics, anthropometric measurements (waist circumference and BMI standard deviation scores (SDS)), days in organic period, total samples number, physical and sedentary activity time and frequency of food categories consumption. BMI SDS was calculated based on the measurements of weight and height, adjusting for age and sex, using the WHO 2007 growth reference standard for children.^12^ Continuous variables were checked for normality with QQ plots and histograms. Categorical variables were described with frequencies and percentages, normally-distributed continuous variables with means and standard deviations, and non-normal continuous variables with medians and interquartile ranges (25th-75th percentiles).

Baseline data were tested for difference between both study groups by

- Chi-squared test for categorical variables
- T-test for normally distributed continuous variables
- Wilcoxon test for non-normally distributed continuous variables

## Conventional period energy analysis

The diet of the children (self-reported food items consumption) during the conventional period was recorded using a food frequency questionnaire (ffq) that was administered to parents through telephone. The ffq was based on the Kafatos food frequency questionnaire^13,14^, that was used originally in the Crete’s population (Greek language). Kafatos was one of the key researchers accompanying Prof. Dontas in the famous 7 countries study’s publications^13^, where this FFQ questionnaire was validated and used in the Crete’s population. Both Cyprus and Crete are Mediterranean, Greek-speaking islands in very close geographic and cultural proximity. The Kafatos FFQ was adapted accordingly to the Cypriot diet^15^ and was used in the Cyprus Research Promotion Foundation study called Cyprus Metabolism Study on 18 yrs. old male soldiers (n-1100) (CYACCESS prospective study).

The mean daily energy intake of the participants was calculated based on a table, that was provided by a registered dietitian and which reported the energy (kcals) of each food item included in the questionnaire (see SAP Appendix).

## Change between baseline and the end of the organic period

The change in outcomes (biomarkers of pesticide exposure and of oxidative stress) between the baseline and the end of the organic period was assessed with the percent change between the last sample of the conventional treatment period (before the start of the organic treatment) and the last urine sample of the organic treatment period. The percent change was estimated only for the participants who completed the full course of the organic treatment. A one-sample T-test was be used to assess whether the percent change was significantly different than zero.

## Overall change between conventional and organic period

The overall change in outcomes between the conventional and organic period was assessed using the overall difference in median levels. The conventional period consists of all conventional samples, including the baseline sample, and the organic period will consist of all organic samples for all participants, regardless of the duration for which they followed the organic treatment. The overall difference in the median levels of the outcomes between the conventional and the organic period was assessed using the unpaired T-test if the outcomes were normally distributed or using the non-parametric Wilcoxon test if the outcomes were non-normally distributed.

## Primary analysis of outcomes

Linear mixed-effect regression models were used for continuous outcomes and logistic models for binary variables (i.e. those with >70%values below LOD), to account for the duration and the effect of treatment. To account for the clustering of participants within schools and the repeated measures within person, the models will include student-level and school-level random intercepts with unsupervised covariance matrix.

Fixed effects were: the treatment condition (organic or conventional) and time (days of treatment, where time=0 was the start of the treatment). An interaction term for the treatment condition and time were considered and dropped when not meeting the threshold of p-value=0.05. The models were adjusted for the baseline value of the outcome in order to account for the background biomarker levels of the participant, The baseline sample was a conventional sample for both groups but it was not consecutive to the conventional phase due to the study design and, thus, it is indicative of each participants exposure prior to participation in the study

In all regression models, the continuous variables, other than time, were centred at the population means.

## Post-hoc analysis

Post-hoc ancillary analyses to help towards the interpretation of the results included:

- Regression models that describe the association of primary outcomes with secondary outcomes adjusting for the baseline levels of each outcome, time, age and sex.
- Regression models that describe the impact of organic diet on participants’ BMI SDS with or without conditioning on the outcomes as candidate mediators. These models included only participants with available anthropometric measurements before and after the organic treatment, hence only two timepoints for the BMI SDS and the biomarkers were used.

## Sensitivity analysis

Two sets of sensitivity analyses were undertaken based on the primary analysis of the outcomes:

- Regression models that were not adjusted for the baseline value of the outcome. Hence, the baseline sample excluded from the analysis.
- Regression models excluding the two participants who followed the opposite order of treatment compared to the group their school was allocated.

## Estimation of the geometric mean ratios and odds ratios

Geometric mean ratios (GMR) of the outcomes and 95% confidence intervals (95% CIs) were estimated exponentiating the regression parameters from the linear mixed-effect regression models. Odds ratios (OR) and 95% CIs were estimated exponentiating the regression parameters from the logistic regression models.

# References

1. European Food Safety Authority (EFSA) Panel on Dietetic Products, Nutrition and Allergies (NDA), Scientific Opinion on Dietary Reference Values for energy. EFSA Journal 2013; 11(1): 3005. doi:10.2903/j.efsa.2013.3005.

2. ATSDR - Toxicological Profile: Pyrethrins and Pyrethroids [Internet]. [cited 2018 May 23];Available from: https://www.atsdr.cdc.gov/toxprofiles/tp.asp?id=787&tid=153

3. Harada, KH, Tanaka K, Sakamoto H et al. Biological Monitoring of Human Exposure to Neonicotinoids Using Urine Samples, and Neonicotinoid Excretion Kinetics. PLoS ONE 2016; 11(1). doi: 10.1371/journal.pone.0146335.

4. NHANES. Anthropometry Procedures Manual. 2007. [cited 2018 May 17]; Available from: <https://wwwn.cdc.gov/nchs/data/nhanes/2007-2008/manuals/manual_an.pdf\>

5. Campbell MK, Piaggio G, Elbourne DR, Altman DG; for the CONSORT Group. Consort 2010 statement: extension to cluster randomised trials. BMJ. 2012 Sep 4;345:e5661. PMID: 22951546

6. Dupont WD and Plummer WD. PS: Power and Sample Size Calculation version 3.1.2, 2014. <http://biostat.mc.vanderbilt.edu/wiki/Main/PowerSampleSize>

7. Koenig W. Update on C-reactive protein as a risk marker in cardiovascular disease. Kidney Int 2003;63:S58–61.

8. Dourado GKZS, Cesar TB. Investigation of cytokines, oxidative stress, metabolic, and inflammatory biomarkers after orange juice consumption by normal and overweight subjects. Food Nutr Res 2015;59(1):28147.

9. Huang T, Larsen KT, Møller NC, Ried-Larsen M, Frandsen U, Andersen LB. Effects of a multi-component camp-based intervention on inflammatory markers and adipokines in children: A randomized controlled trial. Prev Med 2015;81:367–72.

10. Izadpanah A, Barnard RJ, Almeda AJE, et al. A short-term diet and exercise intervention ameliorates inflammation and markers of metabolic health in overweight/obese children. Am J Physiol-Endocrinol Metab 2012;303(4):E542–50.

11. Helsel DR. More than obvious: better methods for interpreting nondetect data. Environ Sci Technol 2005;39:419A-423A.

12. De Onis M. Development of a WHO growth reference for school-aged children and adolescents. Bull World Health Organ 2007; 85(09): 660–7.

13. Kafatos A, Diacatou A, Voukiklaris G, Nikolakakis N, Vlachonikolis J, Kounali D, Mamalakis G, Dontas AS. Heart disease risk-factor status and dietary changes in the Cretan population over the past 30 y: the Seven Countries Study. Am J Clin Nutr 1997;65:1882-1886.

14. Vergetaki A, Papadaki A, Linardakis M. Kafatos A. Changes in 10-year cardiovascular risk and behavioral risk factors in men in Crete, Greece, since the Seven Countries’ Study (1960–1997). J. Public Health (Berl.) (2018) 26: 409. <https://doi.org/10.1007/s10389-017-0879-z>

15. Savva, SC, Y Kourides, M Tornaritis, M Epiphaniou-Savva, C Chadjigeorgiou, and A. Kafatos (2002). Obesity in children and adolescents in Cyprus. Prevalence and predisposing factor. International Journal of Obesity, 26, 1036-1045.

# SAP Appendix

Table Energy analysis for conventional diet: Portions & Food composition source. Food items based on the food frequency questionnaire

| **Food** | **Portion^*^** | **Energy (kcal)** | **Source**  **(Food No/page)^**^** |
| --- | --- | --- | --- |
| **Milk** |  |  |  |
| **Cow’s milk, full fat** | 250ml | 165.0 | 3 (218) |
| **Cow’s milk, semi-skimmed** | 250ml | 115.0 | 3 (212) |
| **Cow’s milk, skimmed** | 250ml | 85.0 | 3 (207) |
| **Evaporated milk, full fat** | 250ml | 377.5 | 3 (231) |
| **Evaporated milk, semi-skimmed** | 250ml | 267.5 | 3 (232) |
| **Evaporated milk, skimmed** | 250ml | 166.7 | 6 (1) |
| **Condensed milk** | 250ml | 832.5 | 3 (228) |
| **Chocolate milk** | 250ml | 157.5 | 3 (234) |
| **Other milk** | Not taken in account for the analysis | | |
| **Yoghurt, plain or with fruits** |  |  |  |
| **Full fat** | 200g | 229.0 | 3 (283 284 291 292) |
| **Reduced fat** | 200g | 134.0 | 3 (287 288) |
| **Fat free** | 200g | 101.0 | 3 (289 290) |
| **Cheese** |  |  |  |
| **Kefalotyri, graviera, kasseri, cheddar** | 30g | 91 | 2 (43) 10 (430) 3 (259) |
| **Feta, halloumi, Anari, edam** | 30g | 86.0 | 3 (270 269) 2 (38 28) |
| **Soft or creamy cheese e.g. Cottage, Philadelphia, La vache qui rit^+^** | 30g | 85.2 | 3 (264 280 267) |
| **Eggs (number)** | 60g^1^ | 97.8 | 3 (341 342) |
| **Breakfast cereals (medium bowl)** |  |  |  |
| **Cornflakes, Rice-Krispies, other low fibre breakfast cereals** | 60g^7^ | 227.0 | 7 (47.2 53.1) |
| **All-Bran, Fruit ‘n Fiber, Μuesli, other high fibre breakfast cereals** | 60g^7^ | 197.3 | 7 (46.1 48.2 51.1) |
| **Chocolate, sugary e.g. Coco-Pops, Frosties** | 60g^7^ | 229.8 | 3 (82) |
| **Bread** |  |  |  |
| **White** | 30g | 72.8 | 3 (53 70 50) |
| **Traditional bread/ Rusk** | 30g | 79.8 | 8 (1 2) |
| **Brown** | 30g | 69.2 | 3 (62 71) |
| **Wholegrain** | 30g | 84.7 | 3(46 67) 2 (56) |
| **Cereals (1/2 cup)** |  |  |  |
| **Rice** | 75g^7^ | 104.6 | 3 (24 17) |
| **Bulgur** | 75g^7^ | 62.3 | 5 (1) |
| **Trahanas** | 15g^7^ – raw/dry | 56.6 | 2 (53) |
| **Pasta** |  |  |  |
| **Pasta, Orzo (1/2 cup)** | 75g^7^ – cooked; 35g – raw/dry | 81.4 | 3 (36) 5 (2) |
| **Stuffed pasta e.g. ravioli, tortellini (1/2 cup)** | 75g^7^ – cooked; 50g – raw/dry | 143.5 | 6 (2) 5 (3) |
| **Oven pasta (makaronia tou fournou) (1 medium piece)** | 150g^7^ | 222.0 | 2 (98) |
| **Potatoes** |  |  |  |
| **Fried (20-25 pieces)** | 100g^7^ | 264.0 | 3 (713 716 723) |
| **Boiled** | 90g | 67.5 | 3 (702) |
| **Baked** | 90g | 134.1 | 3 (710) |
| **Puree (1/2 cup)** | 100g^7^ | 104.0 | 3 (709) |
| **Legumes e.g. lentils, beans (1/2 cup)** | 80g^7^ | 83.6 | 3 (741 742 746 754) |
| **Meat, meat products and fish** |  |  |  |
| **Meat or minced meat: pork e.g. pork chop, sheftalia, souvlaki, souvla** | 90g | 267.3 | 3 (471 462) [2 (109)] |
| **Meat: lamb/goat e.g. souvla, cooked in oven, kleftiko** | 90g | 237.0 | 3 (437 439 458) |
| **Meat or minced meat: burger e.g. steak, hamburger** | 90g | 166.2 | 3 (418 423 424) |
| **Chicken or rabbit** | 90g | 131.0 | 3 (486 508) |
| **Liver** | 90g | 161.1 | 3 (515 518) |
| **Cold cuts e.g. salami, bacon, mortadella (1 slice)** | 10g^1,7^ | 33.3 | 3 (551 392) 6 (5) |
| **Cold cuts e.g.** **lountza, ham (1 slice)** | 30g^1,7^ | 31.8 | 6 (6) 3 (405) |
| **Sausages** | 120g | 247.6 | 2 (124 125) |
| **Meat, tinned e.g. ZWAN** | 120g | 246.0 | 3 (539) |
| **Fish, tinned** | 120g | 172.8 | 3 (668 669) |
| **Fish or seafood, fresh, frozen** | 90g | 151.8 | 3 (619 659 681) |
| **Vegetables and fruits** |  |  |  |
| **Vegetables, fresh, salads (1/2 cup)** | 100g^7^ | 13.0 | 3 (888 858 805) |
| **Vegetables, cooked: greens, green beans, cauliflower (1/2 cup)** | 50g^7^ | 12.2 | 3 (849 749 798) |
| **Fresh fruits (1 portion)** | 130g^1^ | 56.4 | 3 (921 923 933 955 969) |
| **Juices, from fresh fruits or vegetables** | 250ml | 73.3 | 3 (1121 1126 1129) |
| **Oils, spreads and olives** |  |  |  |
| **Olive oil (1Tsp)** | 11g^1,7^ | 134.9 | 3 (380) |
| **Vegetables oil e.g. seed oil and sunflower oil (1Tsp)** | 11g^1,7^ | 134.9 | 3 (388) |
| **Margarine (1tsp)** | 15g^7^ | 111.3 | 3 (357) |
| **Margarine light e.g. Flora light, Vitalite (1tsp)** | 15g^7^ | 83.0 | 3 (362) |
| **Butter (1tsp)** | 15g^7^ | 111.8 | 3 (352) |
| **Mayonnaise (1tsp)** | 15g^7^ | 103.7 | 3 (1198) |
| **Olives (number)** | 6g^7^ | 5.2 | 3 (972) |
| **Sugar, honey, marmalade etc.** |  |  |  |
| **Sugar (1tsp)** | 15g^7^ | 59.1 | 3 (1044) |
| **Honey (1tsp)** | 18g^1,7^ | 51.8 | 3 (1033) |
| **Marmalade or Traditional Cypriot sweet (gliko tou koutaliou) (1Tsp)** | 20g^1,7^ | 52.2 | 3 (1307 1041) |
| **Fruit, tinned (1/2 cup)** | 80g^1,7^ | 36.5 | 3 (925 926 978 979 982 983) |
| **Chocolates, biscuits, ice-cream, sweets, cakes** |  |  |  |
| **Marenda e.g. Nutella (1Tsp)** | 15g^1,7^ | 81.1 | 6 (7) |
| **Chocolate** | 50g | 223.5 | 3 (1049) |
| **Wafers** | 40g | 210.0 | 3 (120 121) |
| **Biscuits, cookies (1 biscuit/cookie)** | 15g^1,7^ | 73.1 | 3 (103 104 110) |
| **Ice-cream (1 scoop)** | 40g^7^ | 77.6 | 3 (306 309 310 313) |
| **Creams e.g. caramel, mousse (1 medium bowl)** | 100g^1,7^ | 126.5 | 3 (318 327) |
| **Cake, tarts(1 medium piece)** | 80g^1,7^ | 219.8 | 3 (314 330 332 335 337) |
| **Snacks and meals** |  |  |  |
| **Greek cheese pie, pourekia, soufflé (number)** | 125^1,7^ | 395.8 | 2 (86, 79) 5 (4) |
| **Greek sausage and chicken pie (number)** | 150^1,7^ | 487.5 | 3 (558 540) |
| **Tahinopita, Greek olive pie (number)** | 150^1,7^ | 570.0 | 2 (87) |
| **Croissant, doughnuts (number)** | 50^1,7^ | 194.0 | 3 (66 160) |
| **Sandwich (cheese or halloumi) (number)** | 120g^1,7^ | 348.0 | 3 (73) |
| **Sandwich (ham or lountza or turkey) (number)** | 120g^1,7^ | 348.0 | 3 (73) |
| **Sandwich (cold cuts and cheese) (number)** | 120g^1,7^ | 348.0 | 3 (73) |
| **Sandwich (tuna etc.) (number)** | 120g^1,7^ | 284.4 | 3 (77) |
| **Pizza (number)** | 90g^1,7^ | 226.3 | 3 (197 198 203 204 205) |
| **Fast food e.g. McDonald’s, KFC (number)** | one meal*** | 1074.5 | 8 (3 4) |
| **Moussakas (number)** | 200g^7^ | 387.0 | 4 (1) |
| **Stuffed vegetables and vine leaves (number)** | 125g^7^ | 226.3 | 2 (96) |
| **Chips, popcorn (small package)** | 28g^1^ | 157.2 | 3 (1077 1079) |
| **Nuts (small coffee cup)** | 30g^7^ | 185.6 | 3 (1004 1006 1018 1028) |
| **Drinks and coffees** |  |  |  |
| **Water (1 glass)** | 200ml | 0.0 | 3 (1232) |
| **Soft drinks, with sugar** | (330ml) | 135.3 | 3 (1107) |
| **Soft drinks, diet** | (330ml) | 3.3 | 3 (1108) |
| **Squash** | (250ml)**** | 68.0 | 7 (96.2) |
| **Fruit drinks** | (250ml) | 92.5 | 7 (94.1 95.1) |
| **Isotonic drinks e.g. Lucozade, Gatorade (1 bottle)** | 500ml**** | 348.2 | 7 (98.2) |
| **Energy drinks e.g. Redbull, Shark (1 bottle)** | 300ml**** | 231.0 | 6 (8 9) |
| **Supplements** | was reviewed and considered unlikely to have any meaningful in macronutrient content | | |
| **Vitamins** | estimated as 0kcals/g for macronutrients | | |
| **Other foods** | Not taken in account for the analysis | | |

*If no reference on portion size, then the portion size used was the available at the food frequency questionnaire. If the portion size was not available from the food frequency questionnaire (i.e. in g or mls) then the reference of the source is available; conversion factor from ml to g =1 was used for all fluids**If more than source is used then the mean was calculated. *** defined as 1 big mag + fries regular + sugary soft drink regular for McDonald’s and 2 chicken breasts + fries regular + sugary soft drink regular for KFC **** made of 30ml squash + 220 water ***** based on online sources.

Tsp = Table spoon; tsp = tea spoon;

**References for the Table:**

^1^Food Standards Agency (FSA; 2005) Food Portion Sizes. 3^rd^ ed. TSO: Norwich

^2^State General Laboratory (SGL; 2013) Composition Tables of Cypriot Foods. Press and Information Office: Nicosia (in Greek)

^3^Food Standard Agency (2006) McCance and Widdowson’s. The Composition of Foods. 6^th^ ed. Royal Society of Chemistry: Cambridge

^4^Hellenic Health Foundation (http://www.hhf-greece.gr/tables/FoodItems.aspx?l=el last accessed 08.06.2018)

(1) Aubergine Moussakas

^5^United States Department of Agriculture Agricultural Research Service USDA Food Composition Databases (https://ndb.nal.usda.gov/ndb/search/list?home=true last accessed 08.06.2018)

(1)Basic Report:  20013, Bulgur, cooked

(2) Full Report (All Nutrients): 45256552, SITOS, ORZO PIQUANT, UPC: 821522000268

(3) Basic Report:  22901, Tortellini, pasta with cheese filling, fresh-refrigerated, as purchased

(4) Basic Report:  11658, Spinach soufflé

(5) Full Report (All Nutrients):  45222374, VEGETABLE COUSCOUS STUFFED BELL PEPPERS, UPC: 5051379085414

^6^**Online resources**

(1) Carnation® Fat Free Evaporated Skim Milk http://www.carnationmilk.ca/En/Products/Carnation-Fat-Free-Evaporated-Skim-Milk last accessed 15.06.2018

(2) Mitsides – Cyprus ravioli http://www.mitsidesgroup.com/productdetails-gr/ravioli/cyprus-ravioli-375g last accessed 15.06.2018

(3) Pastitsio https://akispetretzikis.com/el/recipe/to-pastitsio-toy-akh/print.pdf last accessed 15.06.2018

(4) Sieftalia https://akispetretzikis.com/el/categories/kreas/seftalies last accessed 15.06.2018

(5) Mortadella http://nikas.gr/el/products/μορταδέλα-με-πιπέρι-και-φυστίκι last accessed 15.06.2018

(6) Lounza http://www.grigoriou.com.cy/products.php?product=23 last accessed 15.06.2018

(7) Nutella https://www.nutella.com/en/us/range last accessed 15.06.2018

(8) Gatorade https://www.myfitnesspal.com/food/calories/gatorade-cool-blue-591ml-20-oz-bottle-630496846 last accessed 15.06.2018

(9) Red bull https://www.myfitnesspal.com/food/calories/red-bull-250ml-red-bull-595369265 last accessed 15.06.2018

^7^ Cheyette C. and Balolia Y (2013) Carbs & Cals (eth ed.) Chello Publishing:London

^8^Zorbas (local bakery chain) (1) Athienitiko (2) Elliniko karveli [after personal communication] (3) McDonald’s menu (4) KFC menu [hardcopy]

^9^ Kyriacou, A., Evans, J. M. M., Economides, N. & Kyriacou, A. Adherence to the Mediterranean diet by the Greek and Cypriot population: a systematic review. Eur. J. Public Health 25(6), 1012-1018 (2015).

^10^ Trichopoulou A. (2004) Composition tables of Greek foods. Prisianou A.E.: Athens [in Greek]
