# Supplementary material for: A cluster-randomized crossover trial of organic diet impact on biomarkers of exposure to pesticides and biomarkers of oxidative stress/inflammation in primary school children
Source: PLoS One. 2019 Sep 4;14(9):e0219420. doi: 10.1371/journal.pone.0219420 (PMC6726134; doi:10.1371/journal.pone.0219420)
Supplement: S6 Text — (ZIP) [file pone.0219420.s008.zip › S6 Scripts and input_deidentif/ORGANIKO_manuscript_final_1904_part1_rev.docx]

A cluster-randomized cross over trial of organic diet for primary school children - Part 1

The script was prepared by Corina Konstantinou and Xanthi Andrianou for the statistical analysis of the cluster-randomized crossover trial of organic diet for primary school children, conducted within the EU LIFE+ programme funded project Organiko in Cyprus.

This script includes Part 1 of the analysis (descriptives, description of the change in biomarker levels) and the dataset that will be the input of Part 2 of the analysis.

**Update: April 2019**  Following the manuscript review for PlosOne, the script was revised to include reviewer’s suggestions.

### Table 1 A - Demographic characteristics - Study participants

Demographic & other characteristics of participants with >=12 days in the organic phase

|  | Overall |
| --- | --- |
| n | 149 |
| Group1 = First conventional (%) | 106 (71.1) |
| medulev1 (%) |  |
| Secondary | 26 (17.7) |
| University/college | 80 (54.4) |
| Master/PhD | 41 (27.9) |
| fedulev1 (%) |  |
| Primary | 2 ( 1.4) |
| Secondary | 43 (30.3) |
| University/college | 56 (39.4) |
| Master/PhD | 41 (28.9) |
| Sex = Female (%) | 73 (49.0) |
| age_baseline (mean (SD)) | 11.16 (0.59) |
| Waist_start (median [IQR]) | 69.00 [63.00, 77.00] |
| Waist_endorg (median [IQR]) | 68.00 [63.00, 74.00] |
| Waist_endconv (median [IQR]) | 69.00 [66.50, 75.00] |
| BMI_start (mean (SD)) | 19.24 (3.48) |
| BMI_endorg (mean (SD)) | 18.65 (2.97) |
| BMI_endcon (mean (SD)) | 19.51 (3.35) |
| BMI_for_age_start (%) |  |
| Thinness | 2 ( 1.4) |
| Normal | 90 (60.8) |
| Overweight | 36 (24.3) |
| Obese | 20 (13.5) |
| BMI_for_age_endorg (%) |  |
| Thinness | 2 ( 1.5) |
| Normal | 89 (67.4) |
| Overweight | 28 (21.2) |
| Obese | 13 ( 9.8) |
| BMI_for_age_endcon (%) |  |
| Normal | 19 (54.3) |
| Overweight | 9 (25.7) |
| Obese | 7 (20.0) |
| totsamples (%) |  |
| 2 | 3 ( 2.0) |
| 3 | 3 ( 2.0) |
| 4 | 8 ( 5.4) |
| 5 | 3 ( 2.0) |
| 6 | 132 (88.6) |
| daysorgcat (%) |  |
| 12-21 days | 12 ( 8.1) |
| 22-28 days | 4 ( 2.7) |
| 29-40 days | 133 (89.3) |
| Handinmouth1 = Yes (%) | 36 (24.3) |

Demographic & other characteristics of participants with >=12 days in the organic phase by group

|  | First organic | First conventional | p | test |
| --- | --- | --- | --- | --- |
| n | 43 | 106 |  |  |
| Group1 = First conventional (%) | 0 ( 0.0) | 106 (100.0) | <0.001 |  |
| medulev1 (%) |  |  | 0.871 |  |
| Secondary | 7 (16.7) | 19 ( 18.1) |  |  |
| University/college | 22 (52.4) | 58 ( 55.2) |  |  |
| Master/PhD | 13 (31.0) | 28 ( 26.7) |  |  |
| fedulev1 (%) |  |  | 0.447 |  |
| Primary | 0 ( 0.0) | 2 ( 2.0) |  |  |
| Secondary | 10 (24.4) | 33 ( 32.7) |  |  |
| University/college | 16 (39.0) | 40 ( 39.6) |  |  |
| Master/PhD | 15 (36.6) | 26 ( 25.7) |  |  |
| Sex = Female (%) | 14 (32.6) | 59 ( 55.7) | 0.018 |  |
| age_baseline (mean (SD)) | 11.03 (0.53) | 11.21 (0.61) | 0.101 |  |
| Waist_start (median [IQR]) | 69.00 [66.50, 81.50] | 69.00 [62.00, 76.00] | 0.153 | nonnorm |
| Waist_endorg (median [IQR]) | 69.00 [65.00, 74.00] | 68.00 [62.50, 73.50] | 0.427 | nonnorm |
| Waist_endconv (median [IQR]) | 69.00 [66.50, 75.00] | NA [NA, NA] | NA | nonnorm |
| BMI_start (mean (SD)) | 20.06 (4.08) | 18.91 (3.16) | 0.067 |  |
| BMI_endorg (mean (SD)) | 19.37 (3.29) | 18.37 (2.80) | 0.084 |  |
| BMI_endcon (mean (SD)) | 19.51 (3.35) | NaN (NA) | NA |  |
| BMI_for_age_start (%) |  |  | 0.114 |  |
| Thinness | 0 ( 0.0) | 2 ( 1.9) |  |  |
| Normal | 23 (54.8) | 67 ( 63.2) |  |  |
| Overweight | 9 (21.4) | 27 ( 25.5) |  |  |
| Obese | 10 (23.8) | 10 ( 9.4) |  |  |
| BMI_for_age_endorg (%) |  |  | 0.144 |  |
| Thinness | 0 ( 0.0) | 2 ( 2.1) |  |  |
| Normal | 23 (62.2) | 66 ( 69.5) |  |  |
| Overweight | 7 (18.9) | 21 ( 22.1) |  |  |
| Obese | 7 (18.9) | 6 ( 6.3) |  |  |
| BMI_for_age_endcon (%) |  |  | NaN |  |
| Normal | 19 (54.3) | 0 ( NaN) |  |  |
| Overweight | 9 (25.7) | 0 ( NaN) |  |  |
| Obese | 7 (20.0) | 0 ( NaN) |  |  |
| totsamples (%) |  |  | 0.001 |  |
| 2 | 3 ( 7.0) | 0 ( 0.0) |  |  |
| 3 | 3 ( 7.0) | 0 ( 0.0) |  |  |
| 4 | 0 ( 0.0) | 8 ( 7.5) |  |  |
| 5 | 1 ( 2.3) | 2 ( 1.9) |  |  |
| 6 | 36 (83.7) | 96 ( 90.6) |  |  |
| daysorgcat (%) |  |  | 0.589 |  |
| 12-21 days | 4 ( 9.3) | 8 ( 7.5) |  |  |
| 22-28 days | 2 ( 4.7) | 2 ( 1.9) |  |  |
| 29-40 days | 37 (86.0) | 96 ( 90.6) |  |  |
| Handinmouth1 = Yes (%) | 10 (23.8) | 26 ( 24.5) | 1.000 |  |

### Supplementary Table S1 A - Demographic characteristics - Dropouts comparisons

Demographic & other characteristics of participants with <12 days in the organic phase

|  | Overall |
| --- | --- |
| n | 42 |
| Group1 = First conventional (%) | 18 ( 42.9) |
| medulev1 (%) |  |
| Secondary | 6 ( 18.8) |
| University/college | 22 ( 68.8) |
| Master/PhD | 4 ( 12.5) |
| fedulev1 (%) |  |
| Primary | 1 ( 3.1) |
| Secondary | 7 ( 21.9) |
| University/college | 18 ( 56.2) |
| Master/PhD | 6 ( 18.8) |
| Sex = Female (%) | 15 ( 35.7) |
| age_baseline (mean (SD)) | 11.10 (0.87) |
| Waist_start (median [IQR]) | 70.00 [63.00, 74.50] |
| BMI_start (mean (SD)) | 19.06 (3.27) |
| BMI_for_age_start (%) |  |
| Thinness | 1 ( 4.2) |
| Normal | 13 ( 54.2) |
| Overweight | 6 ( 25.0) |
| Obese | 4 ( 16.7) |
| totsamples (%) |  |
| 1 | 23 ( 54.8) |
| 2 | 1 ( 2.4) |
| 3 | 16 ( 38.1) |
| 4 | 2 ( 4.8) |
| daysorgcat = 0-11 days (%) | 42 (100.0) |
| Handinmouth1 = Yes (%) | 10 ( 31.2) |

Demographic & other characteristics of participants with <12 days in the organic phase by group

|  | First organic | First conventional | p | test |
| --- | --- | --- | --- | --- |
| n | 24 | 18 |  |  |
| Group1 = First conventional (%) | 0 ( 0.0) | 18 (100.0) | <0.001 |  |
| medulev1 (%) |  |  | 0.382 |  |
| Secondary | 4 ( 28.6) | 2 ( 11.1) |  |  |
| University/college | 9 ( 64.3) | 13 ( 72.2) |  |  |
| Master/PhD | 1 ( 7.1) | 3 ( 16.7) |  |  |
| fedulev1 (%) |  |  | 0.612 |  |
| Primary | 0 ( 0.0) | 1 ( 5.6) |  |  |
| Secondary | 2 ( 14.3) | 5 ( 27.8) |  |  |
| University/college | 9 ( 64.3) | 9 ( 50.0) |  |  |
| Master/PhD | 3 ( 21.4) | 3 ( 16.7) |  |  |
| Sex = Female (%) | 9 ( 37.5) | 6 ( 33.3) | 1.000 |  |
| age_baseline (mean (SD)) | 10.81 (0.64) | 11.20 (0.92) | 0.354 |  |
| Waist_start (median [IQR]) | 74.00 [67.00, 77.00] | 68.50 [61.00, 72.75] | 0.105 | nonnorm |
| BMI_start (mean (SD)) | 19.57 (2.97) | 18.81 (3.47) | 0.580 |  |
| BMI_for_age_start (%) |  |  | 0.901 |  |
| Thinness | 0 ( 0.0) | 1 ( 5.6) |  |  |
| Normal | 3 ( 50.0) | 10 ( 55.6) |  |  |
| Overweight | 2 ( 33.3) | 4 ( 22.2) |  |  |
| Obese | 1 ( 16.7) | 3 ( 16.7) |  |  |
| totsamples (%) |  |  | <0.001 |  |
| 1 | 23 ( 95.8) | 0 ( 0.0) |  |  |
| 2 | 1 ( 4.2) | 0 ( 0.0) |  |  |
| 3 | 0 ( 0.0) | 16 ( 88.9) |  |  |
| 4 | 0 ( 0.0) | 2 ( 11.1) |  |  |
| daysorgcat = 0-11 days (%) | 24 (100.0) | 18 (100.0) | NA |  |
| Handinmouth1 = Yes (%) | 3 ( 21.4) | 7 ( 38.9) | 0.501 |  |

Demographic & other characteristics of participants included in the analysis and dropouts

|  | Dropouts | Active participants | p | test |
| --- | --- | --- | --- | --- |
| n | 42 | 149 |  |  |
| Group1 = First conventional (%) | 18 ( 42.9) | 106 (71.1) | 0.001 |  |
| medulev1 (%) |  |  | 0.179 |  |
| Secondary | 6 ( 18.8) | 26 (17.7) |  |  |
| University/college | 22 ( 68.8) | 80 (54.4) |  |  |
| Master/PhD | 4 ( 12.5) | 41 (27.9) |  |  |
| fedulev1 (%) |  |  | 0.282 |  |
| Primary | 1 ( 3.1) | 2 ( 1.4) |  |  |
| Secondary | 7 ( 21.9) | 43 (30.3) |  |  |
| University/college | 18 ( 56.2) | 56 (39.4) |  |  |
| Master/PhD | 6 ( 18.8) | 41 (28.9) |  |  |
| Sex = Female (%) | 15 ( 35.7) | 73 (49.0) | 0.177 |  |
| age_baseline (mean (SD)) | 11.10 (0.87) | 11.16 (0.59) | 0.685 |  |
| Waist_start (median [IQR]) | 70.00 [63.00, 74.50] | 69.00 [63.00, 77.00] | 0.719 | nonnorm |
| Waist_endorg (median [IQR]) | NA [NA, NA] | 68.00 [63.00, 74.00] | NA | nonnorm |
| Waist_endconv (median [IQR]) | NA [NA, NA] | 69.00 [66.50, 75.00] | NA | nonnorm |
| BMI_start (mean (SD)) | 19.06 (3.27) | 19.24 (3.48) | 0.808 |  |
| BMI_endorg (mean (SD)) | NaN (NA) | 18.65 (2.97) | NA |  |
| BMI_endcon (mean (SD)) | NaN (NA) | 19.51 (3.35) | NA |  |
| BMI_for_age_start (%) |  |  | 0.743 |  |
| Thinness | 1 ( 4.2) | 2 ( 1.4) |  |  |
| Normal | 13 ( 54.2) | 90 (60.8) |  |  |
| Overweight | 6 ( 25.0) | 36 (24.3) |  |  |
| Obese | 4 ( 16.7) | 20 (13.5) |  |  |
| BMI_for_age_endorg (%) |  |  | NaN |  |
| Thinness | 0 ( NaN) | 2 ( 1.5) |  |  |
| Normal | 0 ( NaN) | 89 (67.4) |  |  |
| Overweight | 0 ( NaN) | 28 (21.2) |  |  |
| Obese | 0 ( NaN) | 13 ( 9.8) |  |  |
| BMI_for_age_endcon (%) |  |  | NaN |  |
| Normal | 0 ( NaN) | 19 (54.3) |  |  |
| Overweight | 0 ( NaN) | 9 (25.7) |  |  |
| Obese | 0 ( NaN) | 7 (20.0) |  |  |
| totsamples (%) |  |  | <0.001 |  |
| 1 | 23 ( 54.8) | 0 ( 0.0) |  |  |
| 2 | 1 ( 2.4) | 3 ( 2.0) |  |  |
| 3 | 16 ( 38.1) | 3 ( 2.0) |  |  |
| 4 | 2 ( 4.8) | 8 ( 5.4) |  |  |
| 5 | 0 ( 0.0) | 3 ( 2.0) |  |  |
| 6 | 0 ( 0.0) | 132 (88.6) |  |  |
| daysorgcat (%) |  |  | <0.001 |  |
| 0-11 days | 42 (100.0) | 0 ( 0.0) |  |  |
| 12-21 days | 0 ( 0.0) | 12 ( 8.1) |  |  |
| 22-28 days | 0 ( 0.0) | 4 ( 2.7) |  |  |
| 29-40 days | 0 ( 0.0) | 133 (89.3) |  |  |
| Handinmouth1 = Yes (%) | 10 ( 31.2) | 36 (24.3) | 0.555 |  |

### Table 1 B - Children’s activities

Time spent by children outside & in physical and sedentary activities (hours per week) for 149 participants

|  | Overall |
| --- | --- |
| n | 149 |
| garden_week (mean (SD)) | 2.58 (3.95) |
| park_week (mean (SD)) | 0.62 (1.29) |
| run_week (mean (SD)) | 0.78 (2.66) |
| cyc_week (mean (SD)) | 0.55 (1.12) |
| bask_week (mean (SD)) | 0.26 (0.84) |
| foot_week (mean (SD)) | 1.20 (2.43) |
| voll_week (mean (SD)) | 0.13 (0.65) |
| swim_week (mean (SD)) | 0.51 (1.75) |
| danc_week (mean (SD)) | 0.80 (1.79) |
| tv_week (mean (SD)) | 9.83 (6.65) |
| comp_week (mean (SD)) | 2.43 (2.97) |
| tablet_week (mean (SD)) | 3.89 (5.22) |
| mobile_week (mean (SD)) | 3.08 (5.36) |
| outside_time (mean (SD)) | 3.19 (4.12) |
| physactiv_time (median [IQR]) | 4.00 [2.00, 6.00] |
| sedentactiv_time (median [IQR]) | 19.00 [13.00, 28.00] |

Time spent by children outside & in physical and sedentary activities (hours per week) for 149 participants by group

|  | First organic | First conventional | p | test |
| --- | --- | --- | --- | --- |
| n | 43 | 106 |  |  |
| garden_week (mean (SD)) | 2.30 (4.01) | 2.69 (3.93) | 0.592 |  |
| park_week (mean (SD)) | 0.80 (1.25) | 0.54 (1.30) | 0.266 |  |
| run_week (mean (SD)) | 0.26 (0.69) | 0.99 (3.10) | 0.127 |  |
| cyc_week (mean (SD)) | 0.55 (1.07) | 0.56 (1.15) | 0.960 |  |
| bask_week (mean (SD)) | 0.33 (0.80) | 0.24 (0.85) | 0.555 |  |
| foot_week (mean (SD)) | 1.21 (2.35) | 1.19 (2.47) | 0.954 |  |
| voll_week (mean (SD)) | 0.09 (0.43) | 0.15 (0.73) | 0.626 |  |
| swim_week (mean (SD)) | 0.78 (2.19) | 0.41 (1.54) | 0.240 |  |
| danc_week (mean (SD)) | 0.69 (1.46) | 0.84 (1.91) | 0.626 |  |
| tv_week (mean (SD)) | 8.59 (7.32) | 10.33 (6.33) | 0.149 |  |
| comp_week (mean (SD)) | 3.03 (2.59) | 2.18 (3.08) | 0.113 |  |
| tablet_week (mean (SD)) | 3.80 (4.28) | 3.92 (5.57) | 0.892 |  |
| mobile_week (mean (SD)) | 1.87 (3.18) | 3.57 (5.97) | 0.079 |  |
| outside_time (mean (SD)) | 3.10 (4.23) | 3.23 (4.10) | 0.868 |  |
| physactiv_time (median [IQR]) | 3.50 [1.75, 5.50] | 4.00 [2.00, 6.00] | 0.291 | nonnorm |
| sedentactiv_time (median [IQR]) | 20.50 [14.50, 26.50] | 16.40 [12.00, 28.00] | 0.258 | nonnorm |

### Supplementary Table S1 B - Children’s activities - Dropouts comparison

Time spent by children outside & in physical and sedentary activities (hours per week) for dropouts

|  | Overall |
| --- | --- |
| n | 42 |
| garden_week (mean (SD)) | 1.00 (1.97) |
| park_week (mean (SD)) | 0.45 (0.87) |
| run_week (mean (SD)) | 0.02 (0.15) |
| cyc_week (mean (SD)) | 0.26 (0.77) |
| bask_week (mean (SD)) | 0.19 (0.71) |
| foot_week (mean (SD)) | 2.06 (3.12) |
| voll_week (mean (SD)) | 0.21 (0.68) |
| swim_week (mean (SD)) | 0.02 (0.15) |
| danc_week (mean (SD)) | 0.76 (1.74) |
| tv_week (mean (SD)) | 9.37 (7.90) |
| comp_week (mean (SD)) | 2.21 (3.44) |
| tablet_week (mean (SD)) | 2.26 (3.54) |
| mobile_week (mean (SD)) | 2.72 (4.59) |
| outside_time (mean (SD)) | 1.45 (2.41) |
| physactiv_time (median [IQR]) | 2.00 [0.00, 3.00] |
| sedentactiv_time (median [IQR]) | 18.00 [9.93, 26.00] |

Time spent by children outside & in physical and sedentary activities (hours per week) for dropouts by group

|  | First organic | First conventional | p | test |
| --- | --- | --- | --- | --- |
| n | 24 | 18 |  |  |
| garden_week (mean (SD)) | 1.19 (2.34) | 0.75 (1.37) | 0.484 |  |
| park_week (mean (SD)) | 0.50 (0.99) | 0.39 (0.70) | 0.687 |  |
| run_week (mean (SD)) | 0.04 (0.20) | 0.00 (0.00) | 0.393 |  |
| cyc_week (mean (SD)) | 0.42 (0.97) | 0.06 (0.24) | 0.133 |  |
| bask_week (mean (SD)) | 0.21 (0.72) | 0.17 (0.71) | 0.853 |  |
| foot_week (mean (SD)) | 1.18 (2.40) | 2.75 (3.50) | 0.161 |  |
| voll_week (mean (SD)) | 0.29 (0.81) | 0.11 (0.47) | 0.403 |  |
| swim_week (mean (SD)) | 0.00 (0.00) | 0.06 (0.24) | 0.253 |  |
| danc_week (mean (SD)) | 0.83 (1.99) | 0.67 (1.37) | 0.762 |  |
| tv_week (mean (SD)) | 6.92 (6.89) | 12.64 (8.15) | 0.018 |  |
| comp_week (mean (SD)) | 1.99 (3.71) | 2.50 (3.11) | 0.641 |  |
| tablet_week (mean (SD)) | 1.88 (3.38) | 2.78 (3.77) | 0.422 |  |
| mobile_week (mean (SD)) | 1.94 (3.42) | 3.75 (5.75) | 0.211 |  |
| outside_time (mean (SD)) | 1.69 (2.83) | 1.14 (1.73) | 0.472 |  |
| physactiv_time (median [IQR]) | 0.00 [0.00, 3.25] | 2.00 [0.00, 3.00] | 0.626 | nonnorm |
| sedentactiv_time (median [IQR]) | 10.80 [0.00, 28.62] | 19.75 [16.62, 21.38] | 0.124 | nonnorm |

Time spent by children outside & in physical and sedentary activities (hours per week) of participants included in the analysis and dropouts

|  | Dropouts | Active participants | p | test |
| --- | --- | --- | --- | --- |
| n | 42 | 149 |  |  |
| garden_week (mean (SD)) | 1.00 (1.97) | 2.58 (3.95) | 0.013 |  |
| park_week (mean (SD)) | 0.45 (0.87) | 0.62 (1.29) | 0.436 |  |
| run_week (mean (SD)) | 0.02 (0.15) | 0.78 (2.66) | 0.068 |  |
| cyc_week (mean (SD)) | 0.26 (0.77) | 0.55 (1.12) | 0.114 |  |
| bask_week (mean (SD)) | 0.19 (0.71) | 0.26 (0.84) | 0.615 |  |
| foot_week (mean (SD)) | 2.06 (3.12) | 1.20 (2.43) | 0.085 |  |
| voll_week (mean (SD)) | 0.21 (0.68) | 0.13 (0.65) | 0.488 |  |
| swim_week (mean (SD)) | 0.02 (0.15) | 0.51 (1.75) | 0.073 |  |
| danc_week (mean (SD)) | 0.76 (1.74) | 0.80 (1.79) | 0.906 |  |
| tv_week (mean (SD)) | 9.37 (7.90) | 9.83 (6.65) | 0.707 |  |
| comp_week (mean (SD)) | 2.21 (3.44) | 2.43 (2.97) | 0.682 |  |
| tablet_week (mean (SD)) | 2.26 (3.54) | 3.89 (5.22) | 0.060 |  |
| mobile_week (mean (SD)) | 2.72 (4.59) | 3.08 (5.36) | 0.693 |  |
| outside_time (mean (SD)) | 1.45 (2.41) | 3.19 (4.12) | 0.010 |  |
| physactiv_time (median [IQR]) | 2.00 [0.00, 3.00] | 4.00 [2.00, 6.00] | <0.001 | nonnorm |
| sedentactiv_time (median [IQR]) | 18.00 [9.93, 26.00] | 19.00 [13.00, 28.00] | 0.332 | nonnorm |

#### Energy calculation from ffq

Energy (kcal) per week and per day

|  | Energy_Week | Energy_Day |
| --- | --- | --- |
|  | Min. : 6015 | Min. : 859.3 |
|  | 1st Qu.:12217 | 1st Qu.:1745.3 |
|  | Median :14924 | Median :2132.0 |
|  | Mean :15600 | Mean :2228.5 |
|  | 3rd Qu.:18325 | 3rd Qu.:2617.9 |
|  | Max. :31924 | Max. :4560.6 |

According to the Children’s Diet Pyramid for children aged 6-12 years Source: National Directions, Ministry of Health, Cyprus <http://bit.ly/2Lp51hH>

- Milk & milk products: 2-3 portions per day (17.5/week)
- Meat, Beans, Eggs, Nuts, Fish: 2-3 portions per day (150-180 gr) (17.5/week)
- Vegetables: 3-5 portions per day (28/week)
- Fruits: 2-4 portions per day (21/week)
- Cereals, Potato, Rice, Whole-grain products: 6-9 portions per day (52.5/week)
- Fats, Sweets, Oils: 1-3 portions per month (0.5/week)

### Table 1 C - Children’s food frequency

Frequency of food categories consumed by children during the conventional phase (portions per week)

|  | Overall |
| --- | --- |
| n | 135 |
| milkproducts (mean (SD)) | 15.85 (8.82) |
| meatfisheggsnutslegumes (mean (SD)) | 15.05 (7.31) |
| vegetables (mean (SD)) | 5.38 (3.84) |
| fruits (mean (SD)) | 9.70 (6.69) |
| cereals (mean (SD)) | 21.78 (9.52) |
| fats (mean (SD)) | 32.47 (17.70) |

Frequency of food categories consumed by children during the conventional phase by group (portions per week)

|  | First organic | First conventional | p | test |
| --- | --- | --- | --- | --- |
| n | 37 | 98 |  |  |
| milkproducts (mean (SD)) | 15.85 (8.64) | 15.85 (8.93) | 0.999 |  |
| meatfisheggsnutslegumes (mean (SD)) | 10.72 (4.46) | 16.69 (7.52) | <0.001 |  |
| vegetables (mean (SD)) | 3.78 (3.06) | 5.98 (3.95) | 0.003 |  |
| fruits (mean (SD)) | 8.32 (5.33) | 10.22 (7.09) | 0.143 |  |
| cereals (mean (SD)) | 19.45 (11.57) | 22.66 (8.52) | 0.081 |  |
| fats (mean (SD)) | 25.43 (11.27) | 35.13 (18.96) | 0.004 |  |

## Urinary data

Percentage of samples below the LOD and LOQ (Units MDA:umol/l, Creatinine:g/l, 6CN:ug/l, 3PBA:ug/l)

Below LOQ (only for the pesticides and only for the whole period)

6CN (<0.226), 3PBA (<0.146)

- 6CN: 81.85
- 3PBA: 25.76

Below LOD

Creatinine (<0.25), MDA (<0.28), 6CN (<0.075), 3PBA (<0.049)

- Overall
- Creatinine: 0.94
- MDA: 1.17
- 6CN: 71.55
- 3PBA: 23.07
- Organic phase
- 6CN: 76.85
- 3PBA: 31.26
- Conventional phase (including the baseline)
- 6CN: 66.44
- 3PBA: 15.17

## Biomarkers descriptives

### Table 1 D - Percentage of 6-CN values below and above LOD by sample by group

Percentage of 6-CN values below and above LOD for 149 participants by group and by sample

|  | 1:1 | 2:1 | 3:1 | 4:1 | 5:1 | 6:1 | 1:2 | 2:2 | 3:2 | 4:2 | 5:2 | 6:2 |
| --- | --- | --- | --- | --- | --- | --- | --- | --- | --- | --- | --- | --- |
| n | 43 | 42 | 40 | 37 | 37 | 37 | 106 | 106 | 106 | 106 | 98 | 96 |
| cnlod = Above LOD (%) | 16 (37.2) | 14 (33.3) | 8 (20.0) | 10 (27.0) | 9 (24.3) | 19 (51.4) | 40 (37.7) | 32 (30.2) | 30 (28.3) | 24 (22.6) | 22 (22.4) | 19 (19.8) |

### Table 2 - Percentiles of the pesticide metabolites and the biomarkers of oxidative stress/inflammation (overall)

- Note: the tables are based on the imputed data
- Units: 6CN:ng/L, 3PBA:ng/L, MDA:nmol/L, 8-OHdG:ug/L, 8-isopgf2a:ng/L
- This table is not included in the manuscript

Quantiles of the raw concentrations

|  | 0% | 5% | 25% | 50% | 75% | 95% | 100% |
| --- | --- | --- | --- | --- | --- | --- | --- |
| cn_q | 0.04 | 0.89 | 7.08 | 25.40 | 120.50 | 900.75 | 33016.0 |
| pb_q | 7.87 | 73.53 | 268.69 | 1319.00 | 3354.25 | 12763.55 | 71967.0 |
| md_q | 140.00 | 390.00 | 690.00 | 950.00 | 1290.00 | 1963.50 | 2980.0 |
| oh_q | 55.68 | 147.79 | 241.70 | 328.77 | 489.80 | 796.60 | 2039.2 |
| iso_q | 252.88 | 1392.01 | 2515.33 | 3350.47 | 4334.27 | 6466.20 | 13211.6 |

- Units: 6CN:ng/g Cr, 3PBA:ng/g Cr, MDA:nmol/g Cr, 8-OHdG:ug/g Cr, 8-isopgf2a:ng/g Cr
- This table is included in the Supplementary Appendix of the manuscript

Quantiles of the creatinine adjusted concentrations

|  | 0% | 5% | 25% | 50% | 75% | 95% | 100% |
| --- | --- | --- | --- | --- | --- | --- | --- |
| crcn_q | 0.02 | 0.78 | 5.92 | 21.94 | 131.61 | 1003.37 | 22158.39 |
| crpb_q | 4.77 | 51.60 | 292.63 | 1169.23 | 3282.50 | 12580.35 | 59515.28 |
| crmd_q | 189.19 | 526.34 | 708.33 | 851.56 | 1024.79 | 1410.03 | 3846.15 |
| croh_q | 69.39 | 116.67 | 212.25 | 308.89 | 455.89 | 800.99 | 2913.14 |
| criso_q | 260.70 | 1332.41 | 2228.89 | 2981.00 | 3912.45 | 6320.29 | 22507.62 |

### Table 2 A - Percentiles of the pesticide metabolites and the biomarkers of oxidative stress/inflammation during the organic treatment

- Note: the tables are based on the imputed data
- Units: 6CN:ng/L, 3PBA:ng/L, MDA:nmol/L, 8-OHdG:ug/L, 8-isopgf2a:ng/L
- This table is not included in the manuscript

Quantiles of the raw concentrations during the organic treatment

|  | 0% | 5% | 25% | 50% | 75% | 95% | 100% |
| --- | --- | --- | --- | --- | --- | --- | --- |
| cn_qo | 0.04 | 0.87 | 6.55 | 23.71 | 84.46 | 572.80 | 17477.00 |
| pb_qo | 7.87 | 64.07 | 189.17 | 659.00 | 1758.50 | 6055.30 | 71967.00 |
| md_qo | 140.00 | 419.00 | 740.00 | 1020.00 | 1350.00 | 2010.00 | 2980.00 |
| oh_qo | 58.29 | 144.97 | 236.88 | 326.32 | 488.74 | 779.09 | 1369.63 |
| iso_qo | 335.41 | 1524.28 | 2630.26 | 3602.35 | 4597.76 | 6663.58 | 10376.71 |

- Units: 6CN:ng/g Cr, 3PBA:ng/g Cr, MDA:nmol/g Cr, 8-OHdG:ug/g Cr, 8-isopgf2a:ng/g Cr
- This table is included in the Supplementary Appendix of the manuscript

Quantiles of the creatinine adjusted concentrations during the organic treatment

|  | 0% | 5% | 25% | 50% | 75% | 95% | 100% |
| --- | --- | --- | --- | --- | --- | --- | --- |
| crcn_qo | 0.02 | 0.66 | 5.45 | 19.50 | 79.46 | 680.35 | 13761.42 |
| crpb_qo | 4.77 | 45.03 | 164.14 | 575.76 | 1681.08 | 7527.35 | 42484.04 |
| crmd_qo | 237.29 | 552.88 | 724.58 | 846.15 | 1029.20 | 1484.52 | 3846.15 |
| croh_qo | 69.39 | 107.66 | 202.04 | 295.74 | 446.74 | 799.51 | 1575.87 |
| criso_qo | 447.21 | 1321.18 | 2299.91 | 3028.44 | 3955.47 | 6247.93 | 22507.62 |

### Table 2 B - Percentiles of the pesticide metabolites and the biomarkers of oxidative stress/inflammation during the conventional treatment

- Note: the tables are based on the imputed data
- Units: 6CN:ng/L, 3PBA:ng/L, MDA:nmol/L, 8-OHdG:ug/L, 8-isopgf2a:ng/L
- This table is not included in the manuscript

Quantiles of the raw concentrations during the conventional treatment

|  | 0% | 5% | 25% | 50% | 75% | 95% | 100% |
| --- | --- | --- | --- | --- | --- | --- | --- |
| cn_qc | 0.08 | 0.93 | 7.47 | 29.44 | 189.50 | 1377.40 | 33016.0 |
| pb_qc | 16.19 | 106.40 | 714.50 | 2151.00 | 5575.00 | 16068.20 | 42851.0 |
| md_qc | 140.00 | 370.00 | 680.00 | 920.00 | 1190.00 | 1756.00 | 2840.0 |
| oh_qc | 55.68 | 152.25 | 249.97 | 333.60 | 491.13 | 805.10 | 2039.2 |
| iso_qc | 252.88 | 1351.79 | 2455.16 | 3147.20 | 4078.84 | 6188.74 | 13211.6 |

- Units: 6CN:ng/g Cr, 3PBA:ng/g Cr, MDA:nmol/g Cr, 8-OHdG:ug/g Cr, 8-isopgf2a:ng/g Cr
- This table is included in the Supplementary Appendix of the manuscript

Quantiles of the creatinine adjusted concentrations during the conventional treatment

|  | 0% | 5% | 25% | 50% | 75% | 95% | 100% |
| --- | --- | --- | --- | --- | --- | --- | --- |
| crcn_qc | 0.09 | 0.87 | 6.47 | 26.32 | 180.38 | 1407.98 | 22158.39 |
| crpb_qc | 11.47 | 78.93 | 688.88 | 2088.35 | 5273.25 | 15223.72 | 59515.28 |
| crmd_qc | 189.19 | 507.76 | 691.40 | 858.21 | 1016.46 | 1354.57 | 2461.54 |
| croh_qc | 83.69 | 127.22 | 222.02 | 322.60 | 471.06 | 796.15 | 2913.14 |
| criso_qc | 260.70 | 1339.19 | 2112.35 | 2963.74 | 3871.45 | 6319.93 | 21641.70 |

## Percent change - Baseline and after 40 days of organic diet

- Note: 6-CN is not included in the below statistical analyses (>70% of samples below LOD)

### Tables 2 C - Percent change for last conventional sample to last organic sample for all variables except 6-CN

- Outcome: %(last of conventional-end of organic)/last of conventional
- Note: the tables are based on the imputed data
- Units: 3PBA:ng/L, MDA:nmol/L, 8-OHdG:ug/L, 8-isopgf2a:ng/L
- This table is not included in the manuscript

| - est | - imate low | - erCI upp | - erCI pva | - lue |
| --- | --- | --- | --- | --- |
| - logPBA | - 10.0 | - 5.1 | - 14.9 | - 0.000 |
| - logMDA | - -2.7 | - -4.3 | - -1.1 | - 0.001 |
| - logOH | - -1.2 | - -3.4 | - 1.0 | - 0.276 |
| - logISO | - -0.6 | - -2.0 | - 0.8 | - 0.407 |

- Units: 3PBA:ng/g Cr, MDA:nmol/g Cr, 8-OHdG:ug/g Cr, 8-isopgf2a:ng/g Cr
- This table is included in the Supplementary Appendix of the manuscript

| - est | - imate low | - erCI upp | - erCI pva | - lue |
| --- | --- | --- | --- | --- |
| - log-cradjPBA | - 11.4 | - 5.7 | - 17.2 | - 0.000 |
| - log-cradjMDA | - 0.1 | - -1.1 | - 1.2 | - 0.913 |
| - log-cradjOH | - 1.7 | - -0.7 | - 4.0 | - 0.167 |
| - log-cradjISO | - 1.6 | - 0.2 | - 2.9 | - 0.023 |

### Table 3 - Summary tables of pesticide metabolites and biomarkers of oxidative stress/inflammation (overall)

- Note: the tables are based on the imputed data
- Units: 6CN:ng/L, 3PBA:ng/L, MDA:nmol/L, 8-OHdG:ug/L, 8-isopgf2a:ng/L
- This table is not included in the manuscript

Summary table of measurements - non-creatinine adjusted

|  | Overall |
| --- | --- |
| n | 854 |
| cn1000 (median [IQR]) | 25.40 [7.08, 120.50] |
| pb1000 (median [IQR]) | 1319.00 [268.69, 3354.25] |
| md1000 (median [IQR]) | 950.00 [690.00, 1290.00] |
| ohdg (median [IQR]) | 328.77 [241.70, 489.80] |
| isopgf2a1000 (median [IQR]) | 3350.47 [2515.33, 4334.27] |

- Units: 6CN:ng/g Cr, 3PBA:ng/g Cr, MDA:nmol/g Cr, 8-OHdG:ug/g Cr, 8-isopgf2a:ng/g Cr
- This table is not included in the manuscript

Summary table of measurements - Creatinine adjusted

|  | Overall |
| --- | --- |
| n | 854 |
| adj_cn1000 (mean (SD)) | 278.17 (1228.28) |
| adj_pb1000 (mean (SD)) | 3104.21 (5431.39) |
| adj_md1000 (mean (SD)) | 896.30 (302.92) |
| adj_ohdg (mean (SD)) | 366.75 (243.08) |
| adj_isopgf2a1000 (mean (SD)) | 3360.68 (2048.77) |

### Table 3 A - Summary tables of pesticide metabolites and biomarkers of oxidative stress/inflammation by treatment

- Note: the tables are based on the imputed data
- Units: 6CN:ng/L, 3PBA:ng/L, MDA:nmol/L, 8-OHdG:ug/L, 8-isopgf2a:ng/L
- This table is not included in the manuscript

Summary table of measurements per phase - non-creatinine adjusted

|  | Conventional | Organic | p | test |
| --- | --- | --- | --- | --- |
| n | 435 | 419 |  |  |
| cn1000 (median [IQR]) | 29.44 [7.47, 189.50] | 23.71 [6.55, 84.46] | 0.041 | nonnorm |
| pb1000 (median [IQR]) | 2151.00 [714.50, 5575.00] | 659.00 [189.17, 1758.50] | <0.001 | nonnorm |
| md1000 (median [IQR]) | 920.00 [680.00, 1190.00] | 1020.00 [740.00, 1350.00] | 0.001 | nonnorm |
| ohdg (median [IQR]) | 333.60 [249.97, 491.13] | 326.32 [236.88, 488.74] | 0.516 | nonnorm |
| isopgf2a1000 (median [IQR]) | 3147.20 [2455.16, 4078.84] | 3602.35 [2630.26, 4597.76] | <0.001 | nonnorm |

- Units: 6CN:ng/g Cr, 3PBA:ng/g Cr, MDA:nmol/g Cr, 8-OHdG:ug/g Cr, 8-isopgf2a:ng/g Cr
- This table is included in the Supplementary Appendix of the manuscript

Summary table of measurements per phase - Creatinine adjusted

|  | Conventional | Organic | p | test |
| --- | --- | --- | --- | --- |
| n | 435 | 419 |  |  |
| adj_cn1000 (median [IQR]) | 26.32 [6.47, 180.38] | 19.50 [5.45, 79.46] | 0.017 | nonnorm |
| adj_pb1000 (median [IQR]) | 2088.35 [688.88, 5273.25] | 575.76 [164.14, 1681.08] | <0.001 | nonnorm |
| adj_md1000 (median [IQR]) | 858.21 [691.40, 1016.46] | 846.15 [724.58, 1029.20] | 0.338 | nonnorm |
| adj_ohdg (median [IQR]) | 322.60 [222.02, 471.06] | 295.74 [202.04, 446.74] | 0.070 | nonnorm |
| adj_isopgf2a1000 (median [IQR]) | 2963.74 [2112.35, 3871.45] | 3028.44 [2299.91, 3955.47] | 0.259 | nonnorm |

## Boxplots

The boxplots are included in the script as additional material to what is included in the manuscript. The boxplots are not included in the manuscript. In the boxplots of 6-CN and 3-PBA the upper 5% of values have been excluded to make the plots easier to read. Imputed data are included.


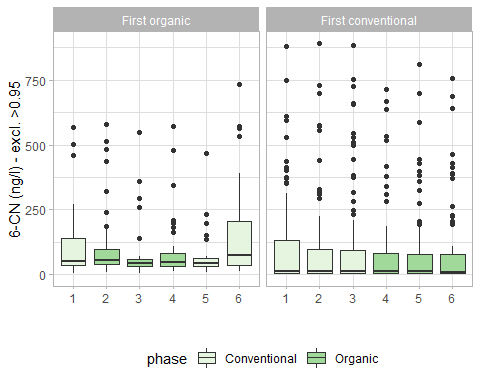

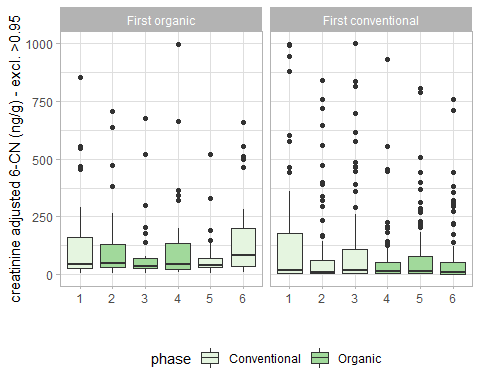

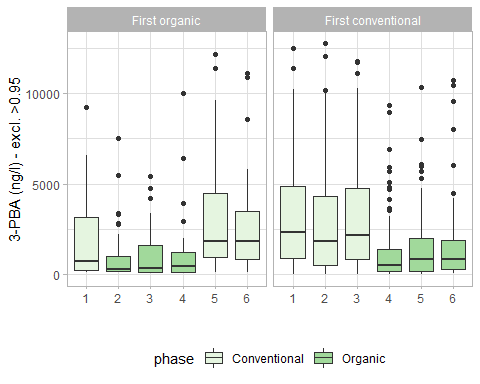

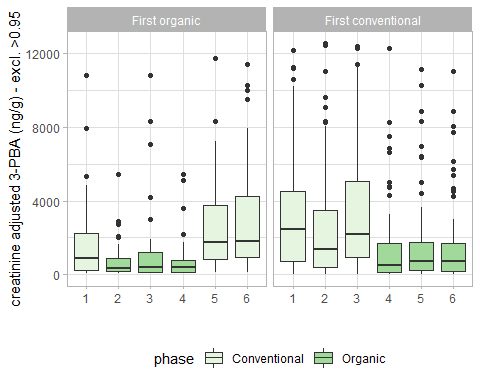

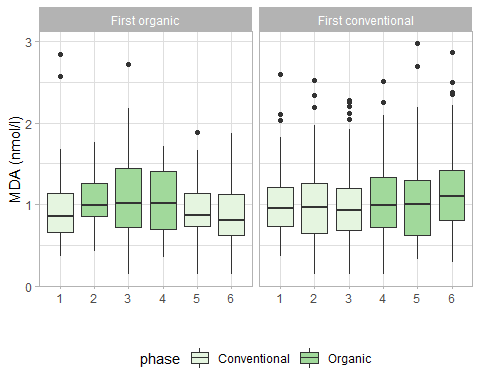

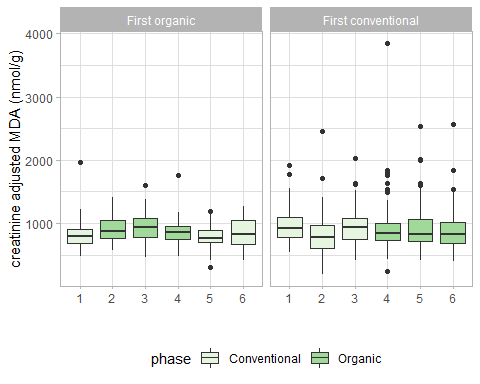


## Warning: Removed 39 rows containing non-finite values (stat_boxplot).


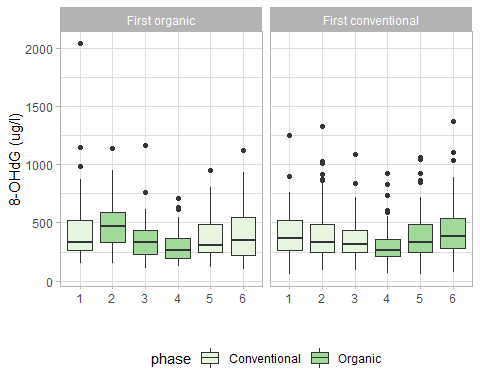


## Warning: Removed 39 rows containing non-finite values (stat_boxplot).


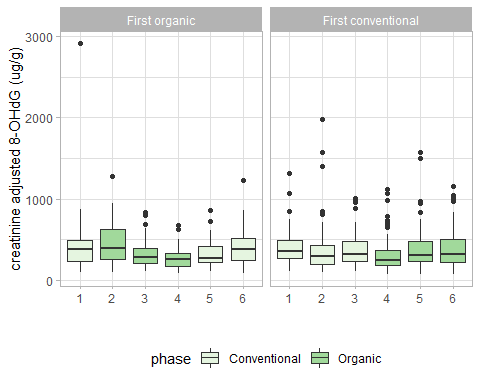


## Warning: Removed 41 rows containing non-finite values (stat_boxplot).


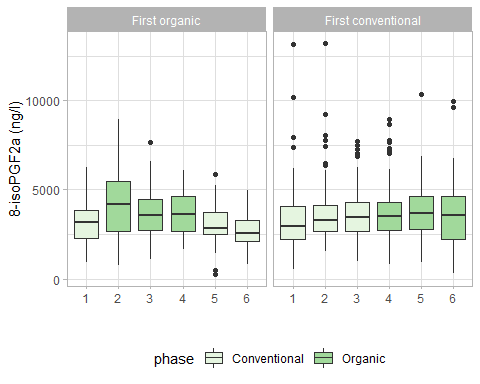


## Warning: Removed 41 rows containing non-finite values (stat_boxplot).


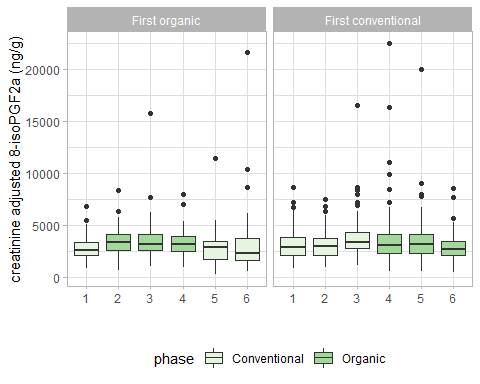


dir_path<-getwd() # working dir to be used in making the new file bel
analysis_rds_dir<-paste0(dir_path, "/produced_data", sep="")
if(!dir.exists(analysis_rds_dir)) dir.create(analysis_rds_dir)

saveRDS(meas1, file.path(analysis_rds_dir, "meas1.rds"))

# Session information

sessionInfo()

## R version 3.5.3 (2019-03-11)
## Platform: x86_64-w64-mingw32/x64 (64-bit)
## Running under: Windows 10 x64 (build 17134)
##
## Matrix products: default
##
## locale:
## [1] LC_COLLATE=English_United States.1252
## [2] LC_CTYPE=English_United States.1252
## [3] LC_MONETARY=English_United States.1252
## [4] LC_NUMERIC=C
## [5] LC_TIME=English_United States.1252
##
## attached base packages:
## [1] stats graphics grDevices utils datasets methods base
##
## other attached packages:
## [1] childsds_0.7.4 NADA_1.6-1 FSA_0.8.22
## [4] scales_1.0.0 reshape_0.8.8 texreg_1.36.23
## [7] lmerTest_3.1-0 lme4_1.1-21 Matrix_1.2-17
## [10] nlme_3.1-137 Rcpp_1.0.1 eeptools_1.2.2
## [13] lubridate_1.7.4 forcats_0.4.0 stringr_1.4.0
## [16] dplyr_0.8.0.1 purrr_0.3.2 tidyverse_1.2.1
## [19] sjPlot_2.6.2 readr_1.3.1 stargazer_5.2.2
## [22] compare_0.2-6 tableone_0.10.0 Hmisc_4.2-0
## [25] ggplot2_3.1.0 Formula_1.2-3 survival_2.43-3
## [28] lattice_0.20-38 tibble_2.1.1 knitr_1.22
## [31] tidyr_0.8.3 data.table_1.12.0 dtplyr_0.0.3
## [34] plyr_1.8.4 readxl_1.3.1
##
## loaded via a namespace (and not attached):
## [1] TH.data_1.0-10 minqa_1.2.4 colorspace_1.4-1
## [4] class_7.3-15 sjlabelled_1.0.17 estimability_1.3
## [7] htmlTable_1.13.1 base64enc_0.1-3 rstudioapi_0.10
## [10] glmmTMB_0.2.3 mvtnorm_1.0-10 xml2_1.2.0
## [13] codetools_0.2-16 splines_3.5.3 mnormt_1.5-5
## [16] sjmisc_2.7.9 jsonlite_1.6 nloptr_1.2.1
## [19] ggeffects_0.9.0 broom_0.5.1 cluster_2.0.7-1
## [22] gamlss.dist_5.1-3 compiler_3.5.3 httr_1.4.0
## [25] sjstats_0.17.4 emmeans_1.3.3 backports_1.1.3
## [28] assertthat_0.2.1 lazyeval_0.2.2 survey_3.35-1
## [31] cli_1.1.0 acepack_1.4.1 htmltools_0.3.6
## [34] tools_3.5.3 coda_0.19-2 gtable_0.3.0
## [37] glue_1.3.1 reshape2_1.4.3 cellranger_1.1.0
## [40] psych_1.8.12 lmtest_0.9-36 insight_0.1.2
## [43] xfun_0.5 rvest_0.3.2 MASS_7.3-51.1
## [46] zoo_1.8-5 hms_0.4.2 parallel_3.5.3
## [49] sandwich_2.5-0 TMB_1.7.15 RColorBrewer_1.1-2
## [52] yaml_2.2.0 gridExtra_2.3 labelled_2.1.0
## [55] rpart_4.1-13 latticeExtra_0.6-28 stringi_1.4.3
## [58] highr_0.8 maptools_0.9-5 e1071_1.7-1
## [61] checkmate_1.9.1 boot_1.3-20 rlang_0.3.2
## [64] pkgconfig_2.0.2 arm_1.10-1 evaluate_0.13
## [67] labeling_0.3 htmlwidgets_1.3 tidyselect_0.2.5
## [70] magrittr_1.5 R6_2.4.0 generics_0.0.2
## [73] multcomp_1.4-10 pillar_1.3.1 haven_2.1.0
## [76] foreign_0.8-71 withr_2.1.2 abind_1.4-5
## [79] sp_1.3-1 nnet_7.3-12 modelr_0.1.4
## [82] crayon_1.3.4 rmarkdown_1.12 grid_3.5.3
## [85] vcd_1.4-4 digest_0.6.18 xtable_1.8-3
## [88] numDeriv_2016.8-1 munsell_0.5.0
