# Supplementary material for: A cluster-randomized crossover trial of organic diet impact on biomarkers of exposure to pesticides and biomarkers of oxidative stress/inflammation in primary school children
Source: PLoS One. 2019 Sep 4;14(9):e0219420. doi: 10.1371/journal.pone.0219420 (PMC6726134; doi:10.1371/journal.pone.0219420)
Supplement: S6 Text — (ZIP) [file pone.0219420.s008.zip › S6 Scripts and input_deidentif/READ ME update Jul 2019.docx]

**R input files**

| **A/A** | **File name** | **Description** |
| --- | --- | --- |
| 1 | Baseline_Ques(Final) 250517-EA.csv | Baseline questionnaire data file 1 |
| 2 | Baseline_Ques(Final) 250517-SI.csv | Baseline questionnaire data file 2 |
| 3 | Baseline_Ques(Final) 250517-IC.csv | Baseline questionnaire data file 3 |
| 4 | Samples and days per participant.csv | Samples per participant and days in organic period |
| 5 | ffq_digitization-main study_KN_250517.csv | Food frequency questionnaire data file 1 |
| 6 | ffq_digitization-main study_EA_250517.csv | Food frequency questionnaire data file 2 |
| 7 | FFQ_calories.csv | Food products and energy/portion file |
| 8 | mda_cr_meas.csv | MDA, creatinine measurements |
| 9 | Pesticides_meas.csv | Pesticides metabolites measurements |
| 10 | OHdG final concentr.csv | 8-OHdG measurements |
| 11 | Final concentrations_8-isoPGF2a.csv | 8-iso-PGF2a measurements |
| 12 | sample_dates.xlsx | Conventional period dates (used in the Part 2 script) |
| 13 | org_start_dates.xlsx | Organic period start dates (used in the Part 2 script) |

The output of the ORGANIKO_manuscript_final_180621_part1 script (produced_data/meas1) is used as the input of the ORGANIKO_manuscript_final_180621_part2 script.

Update: January 2019

The script ORGANIKO_manuscript_final_180621_part2 was revised and it has been renamed: ORGANIKO_manuscript_final_1901_part2_rev. More details on the updates have been included in the script.

Update: April 2019

The script ORGANIKO_manuscript_final_180621_part1 was revised and it has been renamed: ORGANIKO_manuscript_final_1904_part1_rev. The update was the creation of a csv file with the BMI data of the participants (bmidata.csv), as per the request of the reviewer. Below you can see the explanation for the column names.

- Code: Participant ID
- BMI_start: BMI at the beginning of the trial. Calculated using weight/height^2 formula
- BMI_endorg: BMI at the end of the organic treatment. Calculated using weight/height^2 formula
- BMI_start_sds: Standardized BMI for children at the beginning of the trial. Calculated based on WHO 2007 growth references (based on age and sex)
- BMI_endorg_sds: Standardized BMI for children at the end of the organic treatment. Calculated based on WHO 2007 growth references (based on age and sex)
- BMI_for_age_start: BMI categories based on age and sex at the beginning of the trial. Categories based on WHO cut-off points for thinness, normal, overweight and obese (<-2: Thinness, 1>-2: Normal, >1: Overweight, >2: Obese)
- BMI_for_age_endorg: BMI categories based on age and sex at the end of the organic treatment. Categories based on WHO cut-off points for thinness, normal, overweight and obese (<-2: Thinness, 1>-2: Normal, >1: Overweight, >2: Obese)

Update: July 2019

For removal of sensitive info and to ensure anonymization, the following have been done:

- Variables that contain info about the parents’ occupation were removed from the files Baseline_Ques(Final) 250517-EA.csv, Baseline_Ques(Final) 250517-SI.csv, Baseline_Ques(Final) 250517-IC.csv and from the script ORGANIKO_manuscript_final_1904_part1_rev.
- Birthdates and age variables have been removed.
